# Synthesis and Application of the Transition Metal Complexes of α-Pyridinyl Alcohols, α-Bipyridinyl Alcohols, α,α’-Pyridinyl Diols and α,α’-Bipyridinyl Diols in Homogeneous Catalysis

**DOI:** 10.3390/molecules23040896

**Published:** 2018-04-12

**Authors:** Tegene Tole, Johannes Jordaan, Hermanus Vosloo

**Affiliations:** 1 Research Focus Area for Chemical Resource Beneficiation, Catalysis and Synthesis Research Group, North-West University, Hoffmann Street, 2531 Potchefstroom, South Africa; tegenetesfaye@yahoo.ca (T.T.); johan.jordaan@nwu.ac.za (J.J.); 2 Department of Chemistry, College of Natural and Computational Sciences, Hawassa University, Hawassa 1530, Ethiopia

**Keywords:** bipyridinyl diol, catalyst, enantioselective, pyridinyl alcohol, synthesis, transition metal complex

## Abstract

The paper presents a comprehensive survey on the synthetic procedures of transition metal complexes of *α*-pyridinyl alcoholato, *α*-bipyridinyl alcoholato, α,α’-pyridinyl dialcoholato and α,α’-bipyridinyl dialcoholato ligands and their coordination chemistry. Greater emphasis is, however, given to the catalytic activity of the complexes in homogeneous and asymmetric chemical reactions. The multidentate character of the pyridinyl alcohols and/or bipyridinyl diols is of great importance in the complexation with a large number and type of transition metals. The transition metal complexes of pyridinyl alcoholato or bipyridinyl dialcoholato ligands in most cases, and a few pyridinyl alcohols alone, were used as catalysts in homogeneous and chemical asymmetric reactions. In most of the homogeneously catalysed enantioselective chemical reactions, limited numbers and types of pyridinyl alcohols and or bipyridinyl diols were used in the preparation of chiral catalysts that led to a few investigations on the catalytic importance of the pyridinyl alcohols.

## 1. Introduction

Transition metal complexes have played a remarkable role in the development of homogeneous catalysis [1,2]. The existence of many possibilities of combining a catalytically active metal with various chiral ligands cause metal catalysts to continue playing a dominant role in asymmetric catalysis [3].

An important property of pyridinyl alcoholato ligands is their strong basicity, which is mainly due to the lack of resonance stabilisation of the corresponding anion. This strongly basic anionic nature gives them a great ability to create bridges between metal centres rather than to bind to only one metal centre in a terminal fashion [4]. The ability to interact with transition metals both covalently (with oxygen) and through hemilabile coordination (through nitrogen) to form chiral complexes caused the pyridinyl alcohols differentiate the enantioface of aldehydes and ketones [5]. The bidentate, tridentate and also tetradentate character of these ligands enabled them to complex with various transition metals. Apart from their remarkable synthetic utilities in enantioselective homogeneous catalysis, polydentate *N,O*-ligands are used to synthesise ^99^Tc complexes that are potential radiopharmaceutical applications to the heart and brain [6]. Polynuclear alcoholato-bridged iron and manganese complexes received a great deal of attention in the fields of bio-inorganic chemistry and molecular magnetic clusters [7,8,9,10,11,12,13].

The continued efforts of synthetic organic chemists in the development of new chiral reagents and enantioselective methodologies so as to prepare many chiral organic molecules made the preparation of chiral receptor molecules relatively simple [14]. Chiral metal catalysts were found to be important tools for the synthesis of enantiomerically pure organic molecules [15]. The development of a novel chiral catalytic system became the driving force that led many researchers to synthesise chiral pyridinyl alcohols and chiral bipyridinyl diols and utilise them in the various enantioselective synthetic reactions [16].

Although these alcohols have such a remarkable importance and diversified utility, no comprehensive overview of the synthesis and applications of the transition metal complexes of the alcohols have been made to date. Therefore, we compiled an overview of the synthesis of transition metal catalysts of chiral as well as achiral pyridinyl alcoholato and bipyridinyl diol ligands and their applications in the homogeneous and asymmetric catalysis reactions.

## 2. Synthesis and Application of Transition Metal Complexes of Pyridinyl Alcoholato and Bipyridinyl Diol Ligands in Homogeneous Catalysis

The catalysts known today can be classified based on one of the following criteria: structure, composition, area of application, or state of aggregation. According to the state of aggregation, we can classify catalysts into heterogeneous and homogeneous [1]. The topic of this review is homogeneous catalysis and therefore we shall pay attention to the synthesis of pyridinyl alcoholato or bipyridinyl diol ligand-containing transition metal catalysts and their catalytic applications.

### 2.1. Olefin Metathesis

**Scheme 1:**
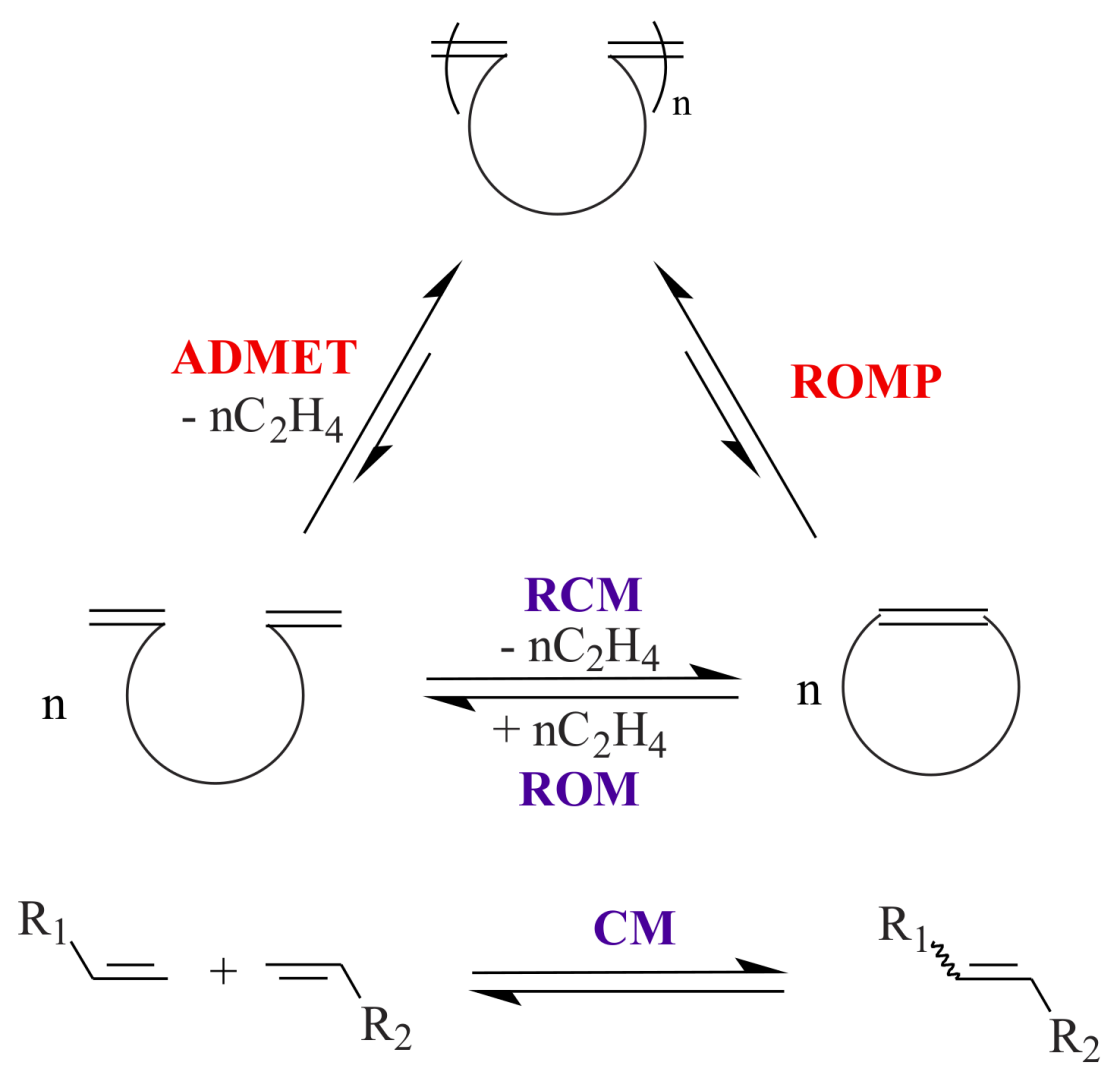
Olefin metathesis reactions.

Olefin metathesis, a carbon-carbon double bond breaking and reforming sequence, became an important method to the synthetic organic and polymer chemist. Olefin metathesis can broadly be classified (See Scheme 1) into ring-closing metathesis (RCM), ring-opening metathesis (ROM), ring-opening cross metathesis (ROCM), ring-opening metathesis polymerization (ROMP), acyclic diene metathesis polymerization (ADMET), and cross-metathesis (CM) [17]. In this section, we shall present the preparation of transition metal-pyridinyl alcohol complexes and their catalytic activity in the homogeneous metathesis reactions.

**Figure 1:**
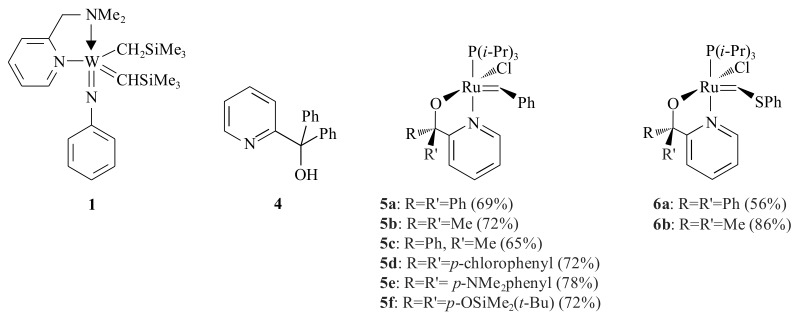
*N*,*N*- and *N*,*O*-bidentate metathesis catalysts.

The ROMP of norbornenes and 2,3-disubstituted norbornadienes has been catalysed by tungsten(VI) alkylidene complexes [18]. The reactivity of these complexes is highly associated with the anciliary ligands present [19]. Complex **1**, a five-coordinate tungsten(VI) alkylidene complex, was synthesized by Van der Schaaf et al. (Figure 1) [20] The complex catalyses the ROMP of norbornene, but it is inert for simple linear alkenes.

**Scheme 2:**
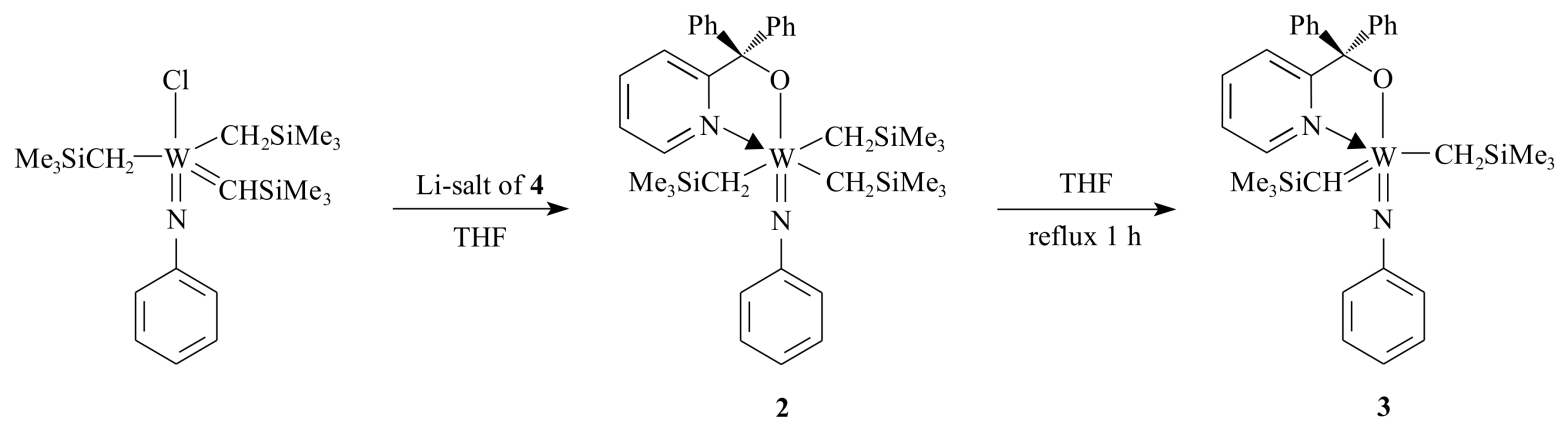
Synthetic procedures of complexes **2** and **3.**

Van der Schaaf and co-workers [21] were interested in improving the catalytic activity of **1** for the metathesis of olefins by manipulating the ligands. They synthesized complex **2,** which is an intermediate in the synthetic procedure of complex **3,** using the pyridinyl alcoholato ligand **4** (see Scheme 2).

The electronic advantage obtained from attaching two alkyl groups on the alkylidene ligand (in complex **2**) is that the electron deficiency of W decreases, and therefore, the donative bond of the imido nitrogen to the W centre weakens [21].

For the purpose of catalysing the RCM of diolefins, synthesis of polymers, isomerisation of olefins and CM of olefins, six Grubbs 1 (**Gr1**) type complexes **5a**–**f** that contained different types of pyridinyl alcoholato ligands were synthesized by Van der Schaaf et al. [22] The complexes were synthesized in such a way that the formation of the lithium salts of the corresponding pyridinyl alcohols was followed by a reaction with the ruthenium compound [RuCl_2_(=CHC_6_H_5_)(P(*i*-Pr_3_))_2_]. With the same procedure, the sulphur analogues of the first two alcoholato-ligands (**6a,b**) were also synthesized in good yields [22].

The ruthenium-complexes **5a** and **5b** were used in the cyclisation of diethyldiallyl malonate into 3-cyclopentene-1,1-dicarboxylic acid diethyl ester (100%, at 60 °C, in Cl_3_CCH_3_) and cyclisation of *N,N*′-di-2-propenylcarbamic acid 1,1-dimethylethyl ester into 2,5-dihydro-1H-pyrrole-1-carboxylic acid 1,1-dimethylethyl ester (100%, at 60 °C, in CHCl_3_). Complex **5a** was also used in the cyclisation of the 5-hexenyl ester of 10-undecenoic acid into oxacyclohexadec-11-en-2-one (50% at 60 °C in toluene) and polymerization of dichloropentadiene.

**Figure 2:**
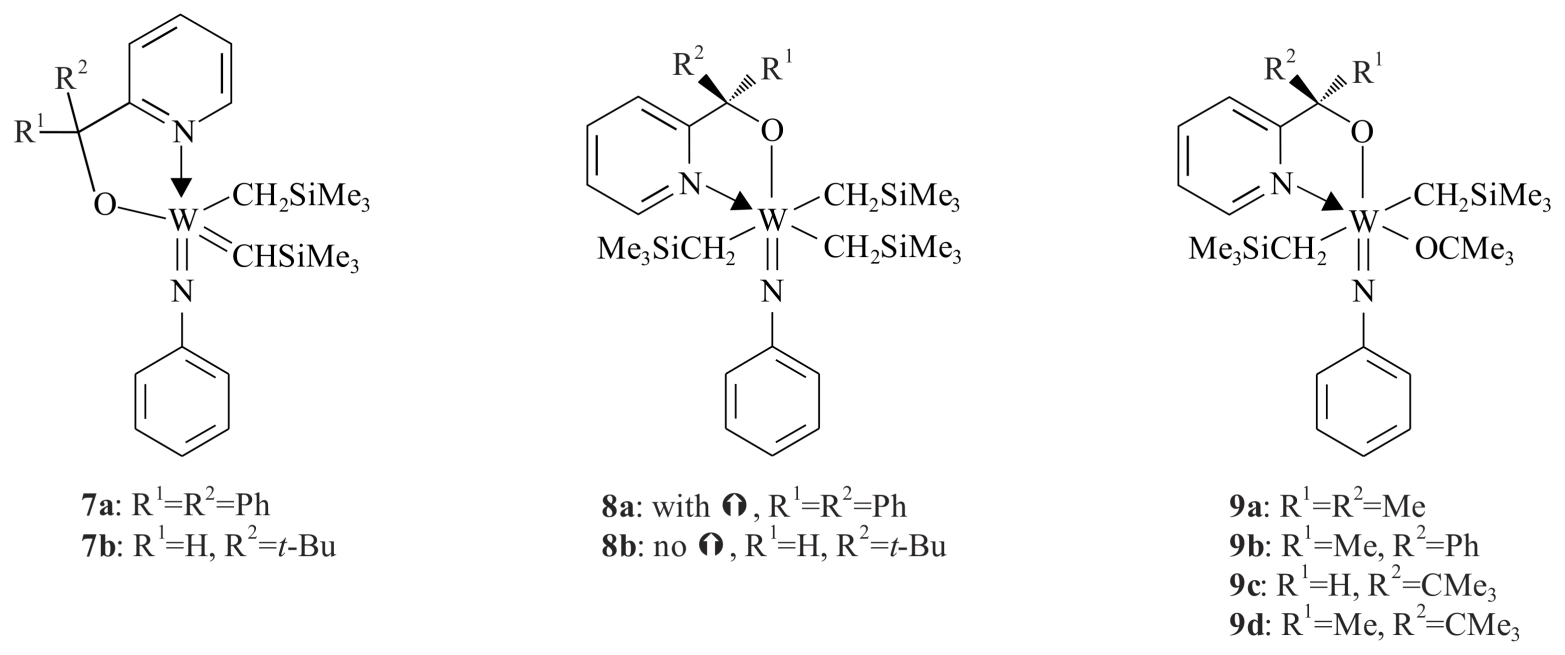
Tungsten(VI) phenylimido complexes.

The tungsten(VI) phenylimido alkylidene complexes **7a,b**, **8a,b** and **9a**–**d** (Figure 2) containing a monoanionic *O*,*N*-chelating ligand (pyridinyl alcoholato) were also synthesized by Van der Schaaf et al. [23] Complexes **7a** and **8a** were synthesized by refluxing the corresponding pyridinyl alcohol lithium salt via transmetallation to the trialkyl tungsten phenylimido chloride [W(CH_2_SiMe_3_)_3_Cl(=NPh)] in THF for one hour. Complex **7a** was produced as a mixture of *anti*:*syn* (1:10) rotamers.

The geometry of **7a** (*syn* isomer) is a distorted square pyramidal in which the alkylidene function occupies the apical position. The metal atom is slightly above the basal plane defined by the *N*-bonded phenyl imido group, the CH_2_SiMe_3_ group and the alkoxy oxygen with the 2-pyridinyl nitrogen of the chelating ligand.

The six coordinated complex **8a** is slightly distorted octahedron in which the phenylimido *N* and the alkoxy groups occupy *trans* positions. The three CH_2_SiMe_3_ groups and the pyridinyl nitrogen are bonded in the equatorial plane. The five coordinated complex **8b**, on the other hand, was synthesized by reacting the lithium salt of the pyridinyl alcohol with the trialkyl tungsten phenylimido chloride at room temperature. Refluxing complex **8b** for four hours resulted in complex **7b** (*anti*:*syn*, 4:6). The six coordinated complex **9** was also synthesized at room temperature via reacting the lithium salt of the corresponding pyridinyl alcohol with dialkyl-*tert*-butoxytungsten phenylimido chloride [W(CH_2_SiMe_3_)_2_Cl(=NPh)(OCMe_3_)]. The substituents in complex **9** play a less important role in W-complexes.

Complexes **7a,b** catalysed the ROMP of norbornene to give polymeric cyclopentenes at 70 °C (≥90% *cis*-vinylene bonds). The alkyl substituents (R^1^ and R^2^) on the α-C of the pyridinyl alcoholato ligand can be easily varied, making these ligands readily tuneable. This tuning has enabled the isolation and characterisation of alkylidenes **7a,b** and their precursor complexes.

The decreasing order of the relative basicity of the methoxide ligands in the complexes **7a,b** is assumed to be OCH(CMe_3_)(2-py) > OCPh_2_-(2-py). The strong basicity of the ligand in **8b** decreased the acidity of W and therefore the pyridine nitrogen does not coordinate to W. In **8a,** the pyridine nitrogen coordinates due to less basicity of the ligand, which makes the W more acidic. Complex **9** is thermally more stable than **8a,b**. None of complexes **9a**–**d** gave an alkylidene complex, even after refluxing for 24 h. Generally, metal atoms in a high valent early-transition-metal complex decrease in electron deficiency when an alkylidene functionality is formed from two alkyl groups via an H_α_-abstraction reaction. These electronic advantages for a d^0^-metal complex are illustrated by the two molecular structures of **7a** and **8a**. The *syn* rotamers result in a five coordinate complex and the potentially bidentate ligands in the *anti* rotamers monodentate bond due to steric reasons. Complexes **7a**,**b** showed low activity in the ROMP of norbornene at low temperature, but significantly increased the rate of ROMP when increasing the temperature due to the high Lewis acidity of W to interact readily with the olefinic double bond of norbornene. The complexes can polymerise the ROM reaction of strained cyclic olefins other than the ROMP of norbornene; however, they are inert towards linear olefins.

**Figure 3:**
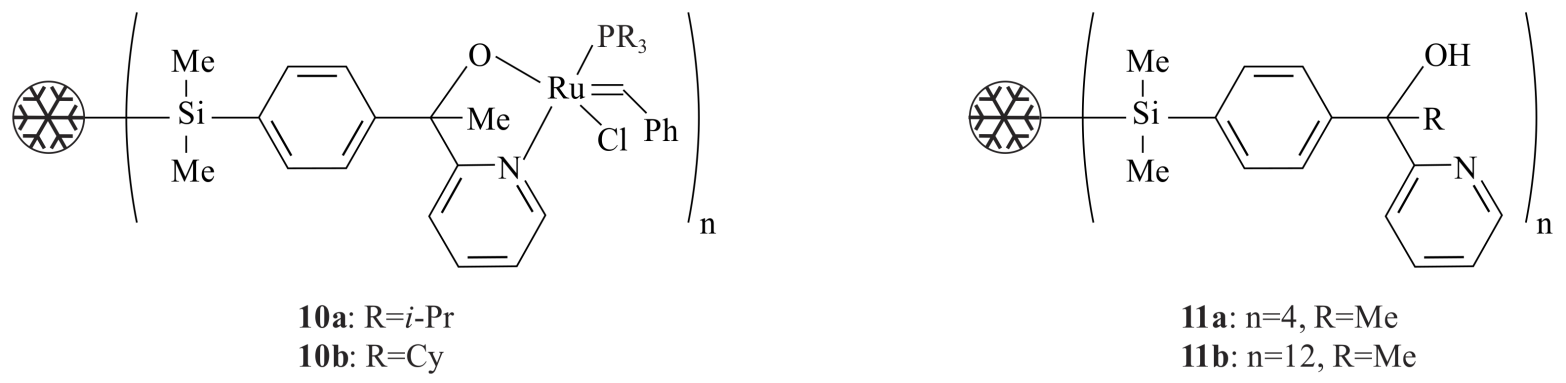
Dendritic pyridinyl alcohols and Ru complexes.

The dendritic ruthenium complexes **10a**,**b** (Figure 3) were synthesized from the dendritic pyridinyl alcohols **11a**,**b** (Figure 3) via treatment with *n*-BuLi and PhC(H)=RuCl_2_(PR_3_)_2_ [24]. Complexes **10a,b** catalysed the RCM reaction (at 80 °C) of diethyl diallyl malonate with 100% conversion after 30 min. The result obtained is comparable to the unimolecular catalyst. The conversion to diethyl-3-cyclopentene dicarboxylate stopped after about 20% conversion in an experiment that was carried out in a solution where the catalyst is separated from the substrate and product by a nanofiltration membrane MPS-60. The use of a dendritic pyridinyl alcoholato ligand is very important to easily remove the catalyst from the product.

**Figure 4:**
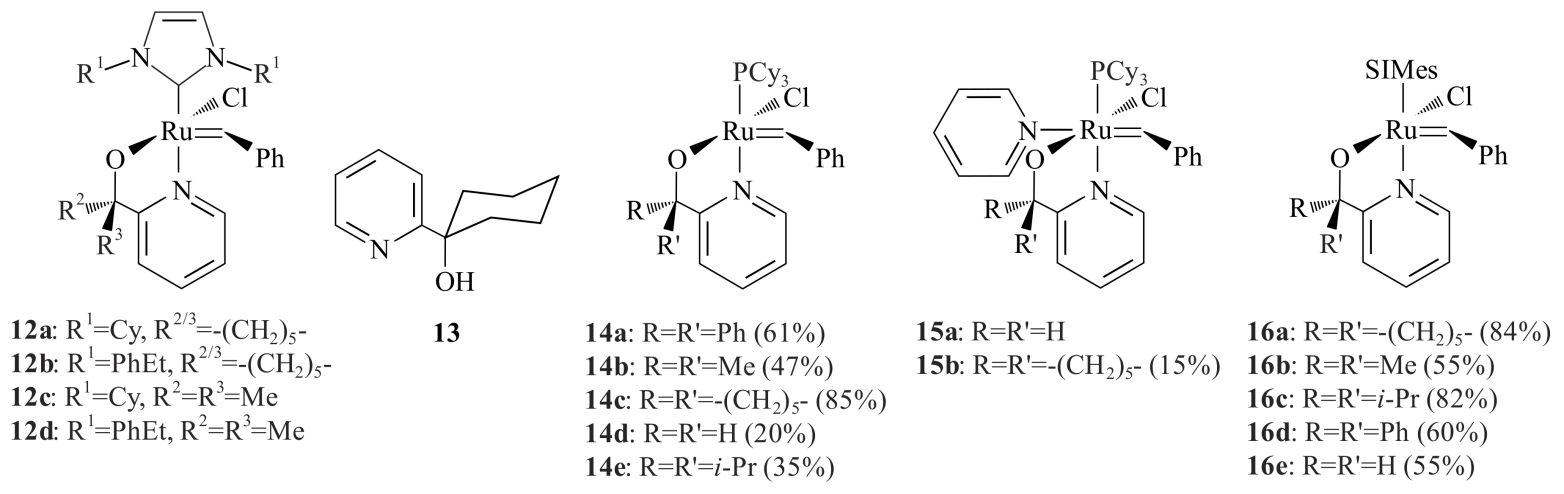
Pyridinyl alcoholato ruthenium carbene complexes.

Ruthenium alkylidene complexes bearing one N-heterocyclic carbene (NHC) and one pyridinyl alcoholato ligand, **12a**–**d** (Figure 4) were synthesized by two different procedures by Denk et al. [25] The first procedure involves treatment of the **Gr1** complex with the lithium salt of the pyridinyl alcoholato ligand **13** followed by the addition of the NHC ligand. In the second procedure, the **Gr1** complex was treated with the NHC ligand, followed by a reaction with the lithium salt of the pyridinyl alcoholato ligand **13**. Complexes **12a,b** were synthesized via the first procedure with 73% **12a** (the yield of **12b** was not reported), while complexes **12c,d** were synthesized through the second procedure and resulted in 73% and 46% yield, respectively [25].

The complexes were tested for the ROMP of norbornene and cyclooctene at room temperature and at 60 °C. Complexes **12a** and **12d** resulted in oligomers at room temperature, while **12b** and **12c** yielded 57% and 5% (norbornene) and 18% and 14% (cyclooctene), respectively. At 60 °C, norbornene underwent ROMP with **12a** (100%), **12b** (98%), **12c** (99%) and **12d** (100%) and cyclooctene **12a** (75%), **12b** (78%), **12c** (72%) and **12d** (80%) [17,25]. The W and Ru complexes of pyridinyl alcoholato ligands in complexes **7** and **12** share similarities in yielding less at low temperatures and high at high temperature in the ROMP of norbornene, which was comparable to **Gr2**.

Jordaan and Vosloo [26,27] synthesized four different types of pyridinyl alcoholato ligands using Herrmann et al.’s [28] procedure for the purpose of synthesising the Grubbs-type catalysts for the metathesis of 1-octene. They prepared the lithium salts of the four pyridinyl alcoholato ligands in a similar procedure to Van der Schaaf et al. [22], which were then reacted with the **Gr1**, **Gr1**-**py** and **Gr2** to produce the ruthenium alkylidene complexes **14**–**16** (Figure 4) bearing the stable NHC and chelating pyridinyl alcoholato ligands. The incorporation of pyridinyl alcoholato ligands with the Grubbs-type catalysts has shown an increase in the thermal stability, activity (in the metathesis of 1-octene) and lifetime of the catalyst [26,27,29].

**Figure 5:**
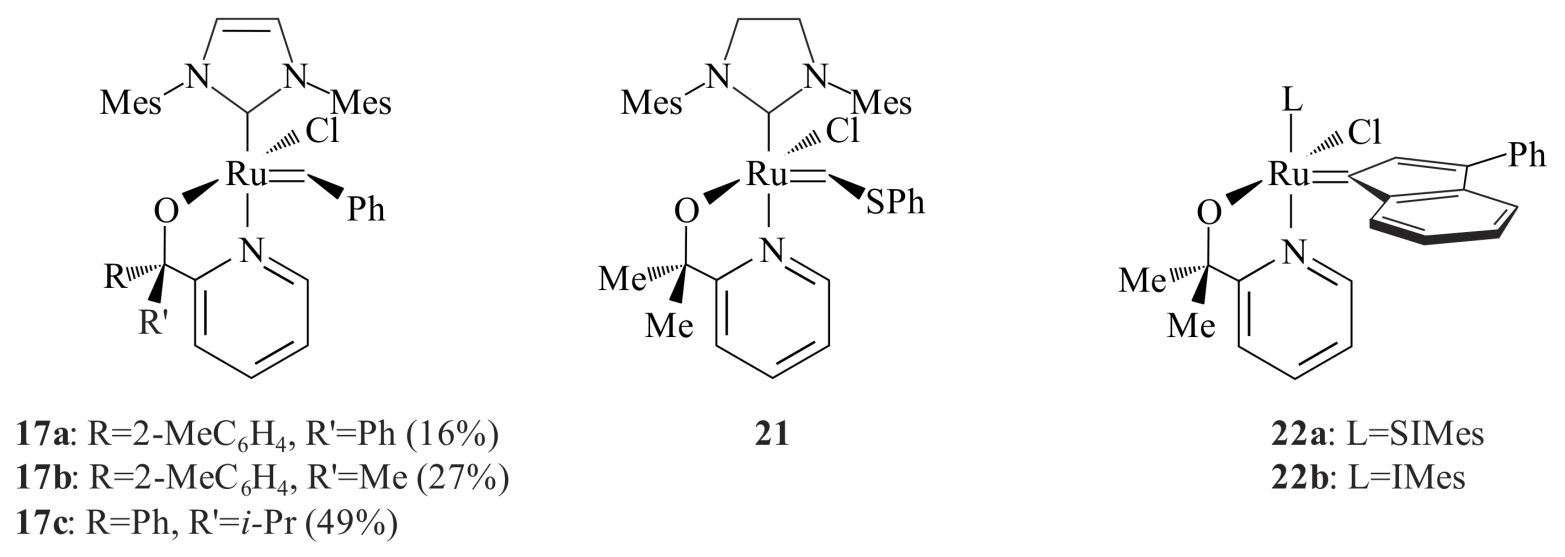
NHC pyridinyl alcoholato ruthenium carbene complexes.

Vosloo and co-workers [30,31] further investigated the activity, stability and selectivity of **Gr2**-type catalysts for the metathesis of 1-octene. With this aim, five different types of pyridinyl alcoholato ligands and the corresponding **Gr2**-type complexes **17a**–**c** (Figure 5) were synthesized. The same procedure as Jordaan [26,27] was followed, however, the synthesis of complexes using pyridinyl alcohols made up of adamantanone and camphor was not successful [30]. The complexes **17a**–**c** showed low activities compared to **16d** for the metathesis reaction of 1-octene. They showed, however, high stability and turnover numbers (TONs) compared to **Gr2**. At high temperature, the activity of the complexes increased with decreasing selectivity. The combination of the NHC ligand and the chelating ability of the pyridinyl alcoholato ligands are responsible for the stability and activity of the catalyst at high temperatures. Incorporating other electron-donating (OMe) and electron-withdrawing (Cl) at the 2- or 4-position on one of the α-phenyl groups of **16d** showed improved catalytic performance for 1-octene metathesis at temperatures ranging from 80–110 °C [32]. The 4-methoxy-substituted complex outperformed the rest at 110 °C (96% conversion with 95% selectivity towards the major products 7-tetradecene and ethene). A modelling study suggested that the improved catalytic performance can be attributed to steric repulsion between the substituted phenyl group and the NHC ligand resulting in strengthening the Ru-N bond [32]. Slugovc and Wattel [33] patented a number of 8-quinolinolate **Gr2** derivatives for use in ROMP reactions. The latter was found to be quite inactive (<1% conversion) for 1-octene metathesis at 60 °C [31].

**Scheme 3:**
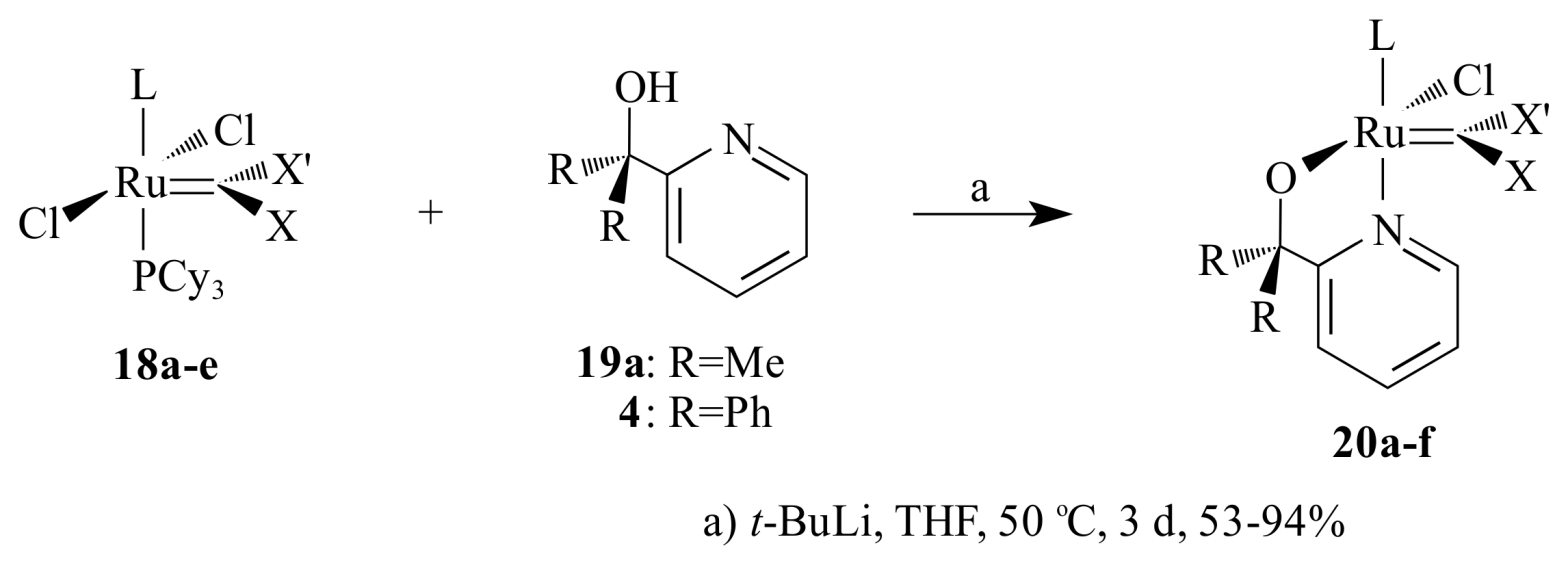
Synthesis of pyridinyl alcoholato ligands from **Gr1** and **Gr2** ruthenium carbenes.

The removal of trace amounts of residual ruthenium catalyst and/or catalyst degradation products in the area of pharmaceutical application is one of the challenging aspects in olefin metathesis reactions. One important method of ruthenium removal is adsorption onto surfaces [34]. The minimum possible amount of ruthenium catalyst residue for end products in the pharmaceutical industries should be less than 10 ppm. However, the adsorption techniques used could not do so. To fill this gap, Schachner et al. [35] synthesized six new ruthenium complexes that contained pyridinyl alcoholato ligands from Grubbs’ 1st and 2nd generation ruthenium carbenes, as shown in Scheme 3 and Table 1.

The catalytic activities of the precatalysts were evaluated with the ROMP of cyclooctene, CM of 5-decene with 5-hexenylacetate and the RCM of 5-hexen-1-yl-10-undecenoate and most of them showed superior activity compared to the commercially available precatalysts in CM and RCM reactions. Even though they showed moderate activity in ROMP, it could be considered as an important aspect from an industrial application point of view. All complexes showed a very high affinity to untreated, unmodified and commercially available chromatography-grade silica [35]. This led the residual ruthenium concentration in the unprocessed reaction mixture to below 10 ppm, which is a prerequisite for fine-chemical application [13].

With the aim of developing heterogeneous catalysts for metathesis, Cabera et al. [36,37] made a systematic evaluation of the activity and stability of five Grubbs-type precatalysts **16b**, **20a**, **21** and **22a,b** that contained pyridinyl alcoholato ligands for a given metathesis (RO-RCM and CM) within the context of a biphasic solid/liquid system. The reactions were done by adsorbing the substrate and catalyst on a thin layer silica plate and developing the TLC in EtOAc/hexane (1:7 *v*/*v*) for CM and hexane for RO-RCM. The substrate for self-CM was methyl 9-dodecene and for RO-RCM *cis*-cyclooctene. None of them gave a product spot at room temperature for the CM. Only **22a** showed activity for the RO-RCM of *cis*-cyclooctene forming 1,8-cyclohexadecadiene among many oligomeric multiples of cyclooctene.

Manipulation of the ligands around the transition metal (W and Ru) complexes showed increment both in the activity and stability on the metathesis reactions of olefins. The chelation ability of the pyridinyl alcohol nitrogen combined with the stable NHC ligand is responsible for the increment in the activity and stability of the catalysts. The investigations of Vosloo and co-workers [26,27,29,30,31,32] were focused on the steric and electronic effects of the groups on the carbon atom to which the alcohol function was attached in the pyridinyl alcohol. Even though the electronic effect of the substituents did not show significant influence on the catalyst activity, the steric effect is clearly seen on the **16d** precatalyst stability.

We did not find any literature on the investigation of the electronic and steric effects of substituents (on the pyridine ring) on the chelation efficiency of pyridinyl alcoholato ligands. Therefore, the chelation ability of the pyridinyl alcohols needs further investigation by synthesising catalysts with electron-withdrawing and/or electron-donating groups on the pyridine ring. The hemilabile nature of the pyridinyl alcoholato ligands could possibly be manipulated by putting either electron-donating or electron-withdrawing groups on the pyridine ring.

### 2.2. Olefin Polymerization

**Figure 6:**
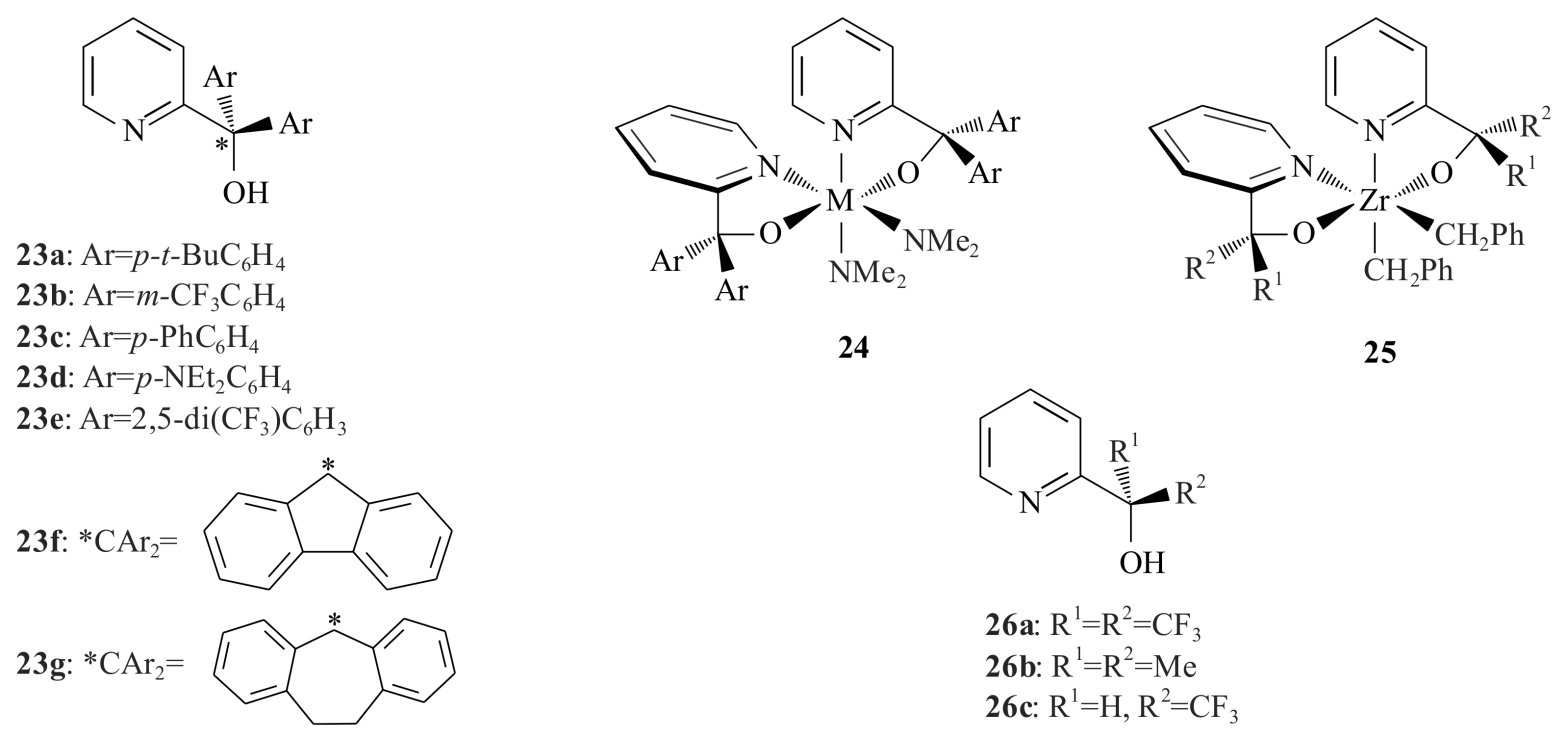
Ligands and complexes used in polymerization reactions.

The pyridinyl alcohols **23a**–**g** (Figure 6) were used by Kim et al. [38] in order to synthesize group four metal (pyCAr_2_O)_2_M(NR_2_)_2_ complexes containing bidentate pyridine. They reacted M(NMe_2_)_4_ (where M = Ti, Zr, and/or Hf) with the pyridinyl alcohols **23a**–**g** to obtain complexes **24a**–**g** in very good yields.

All ligands **23a**–**g** reacted with Ti(NMe_2_)_4_ to yield the complexes **24a**–**g** with 98%, 88%, quantitative, 82%, 95%, 55% and 59% respectively. With Zr ligands **23a**, **23b** and **23e** resulted complex **24a**–**c** with 92%, 67% and 62% yields using the same procedure, respectively. Ligands **23c,d** resulted in the complex (pyCAr_2_O)_x_Zr(NMe_2_)_4-x_ (x = 1–3) with 65% yield. Using three- and four-equivalents of **23c** and **23e** can result in the *tris* and *tetrakis* ligand species of Zr.

The complexes (pyCAr_2_O)_2_Hf(NMe_2_)_2_ of **23a** (92%) and **23e** (58%) were synthesized and have similar structures as that of Ti and Zr complexes, while **23b** and **23c** yielded mixtures of products. Three equivalents of **23b,c** resulted in the *tris*(ligand)species (py(Ar_2_O)_3_Hf(NMe_2_) in 54% and 82%, respectively. The formation of the product mixtures is due to comparable rates of reaction of pyridinyl alcohol with (pyCAr_2_O)M(NMe_2_)_2_, M(NMe_2_)_4_ and/or (pyCAr_2_O)M(NMe_2_)_3_. More acidic pyridinyl alcohols are less selective for the desired (pyCAr_2_O)M(NMe_2_)_2_ species.

X-ray crystallography revealed that these complexes have *C_2_*-symmetric structures in the solid state and in solution, but undergo facile inversion of configuration at the metal, with racemisation of configuration of barriers in the range of 12–14 kcal/mol. The Ti and Zr complexes of the type (pyCAr_2_O)_2_M(NMe_2_)_2_ of pyridinyl ligands **23a**, **23b**, **23d**, and **23e** (**23d,e** only for Ti) were tested in the polymerization reaction of ethylene in the presence of the Lewis acid Al(*i*-Bu)_3_ and methylalumoxane (MAO) as cocatalysts and resulted in a 0.17–1.5 g yield in toluene at 1 atm ethylene and 43 °C with Zr catalysts more highly active than Ti complexes [38]. The polymerization of ethylene has been investigated using the in situ alkylation/activation protocol developed for Cp_2_Zr(NMe_2_)_2_ metallocene amide compounds [39]. The AlR_3_ reagent and activator would generate (pyCAr_2_O)_2_M(R)^+^ or (pyCAr_2_O)_2_M(H)^+^ species in situ. The Ti and Zr complexes are activated in situ for the polymerization of ethylene.

A Zr complex **25** (Figure 6) having distorted octahedral structure with *trans*-O, *cis*-N, *cis*-C ligand arrangement, but that undergoes inversion of configuration at the metal centre at elevated temperature resulted in a reaction with pyridinyl alcoholato ligands **26a**,**b** and **27** with Zr(CH_2_Ph)_4_ in toluene or benzene [40]. Alkyl abstraction from **25** by treatment with the benzene solution of B(C_6_F_5_)_3_ or Zr-R protonolysis reactions resulted in cationic five-coordinate species (pyCR_2_O)_2_Zr(CH_2_)Ph^+^. These species adopted distorted square pyramidal structures in which the ligand arrangement is strongly influenced by the π-donor properties of the alkoxide ligand. The cationic complexes obtained via the aforementioned reaction of **25** (of ligands **26a** and **27**) with the Lewis acid B(C_6_F_5_)_3_ catalyse ethylene polymerization with very low activity resulting in much lower molecular weight polymers, while the cationic complex resulted from **25** with ligand **26b** did not polymerise ethylene at all. This clearly showed that the electron withdrawing CF_3_ is beneficial and also the presence of relatively electron-donating pyCMe_2_O^-^ ligand in the cationic complex made of **25** (with ligand **26b**). The detection of the benzyl end groups by ^1^H NMR suggested that the polymerization is initiated via insertion into the Zr-CH_2_Ph bond. The cationic complex of **25** that is prepared with ligand **26a** undergoes polymerization of 1-hexene to result in polyhexene in moderate activity under mild conditions (CD_2_Cl_2_, 23 °C). This trend is opposite to that reported for metallocene catalysts, in which incorporation of electron-donating substituents on the cyclopentadiene (Cp) ligands generally increases activity in the absence of overriding steric factors [41].

**Figure 7:**
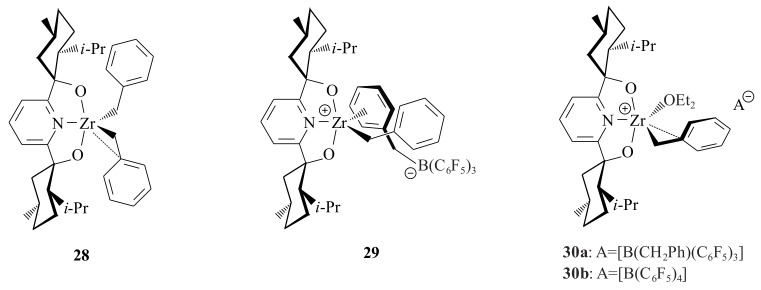
Neutral and cationic pyridinyl dialcoholato complexes of Zr.

The Zr complex **28** [Zr(BMP)(CH_2_Ph)_2_] (Figure 7) was synthesized from 2,6-*bis*[(1*S*,2*S*,5*R*)-(−)-menthoxo]pyridinyl diol, (BMP)H_2_, via treatment with Zr(CH_2_Ph)_4_ [42]. Treatment of **28** by one equivalent of B(C_6_F_5_)_3_ in C_6_D_6_ or C_6_D_5_Br resulted in complex **29**. The reaction of **29** with Et_2_O in C_6_D_5_Br yielded complex **30a**. Complex **30b** was prepared by abstraction of one of the benzyl ligands with [CPh_3_]^+^[B(C_6_F_5_)_4_]^-^ from **28**. Complex **29** undergoes single insertion of ethylene into its Zr-carbon bond to yield [Zr(BMP)(CH_2_CH_2_CH_2_Ph)][(η^6^-PhCH_2_)B(C_6_F_5_)_3_]. α-Olefins and cyclooctadiene, on the other hand, afforded diastereomeric mixtures of the general formula [Zr(BMP)(η^1^:η^6^-CHRCHR’CH_2_Ph)]^+^ [B(CH_2_Ph)(C_6_F_5_)_3_]^-^. It was reported that the complexes might lead to higher insertion stereoselectivities of olefins and actual polymerization [42].

**Scheme 4:**
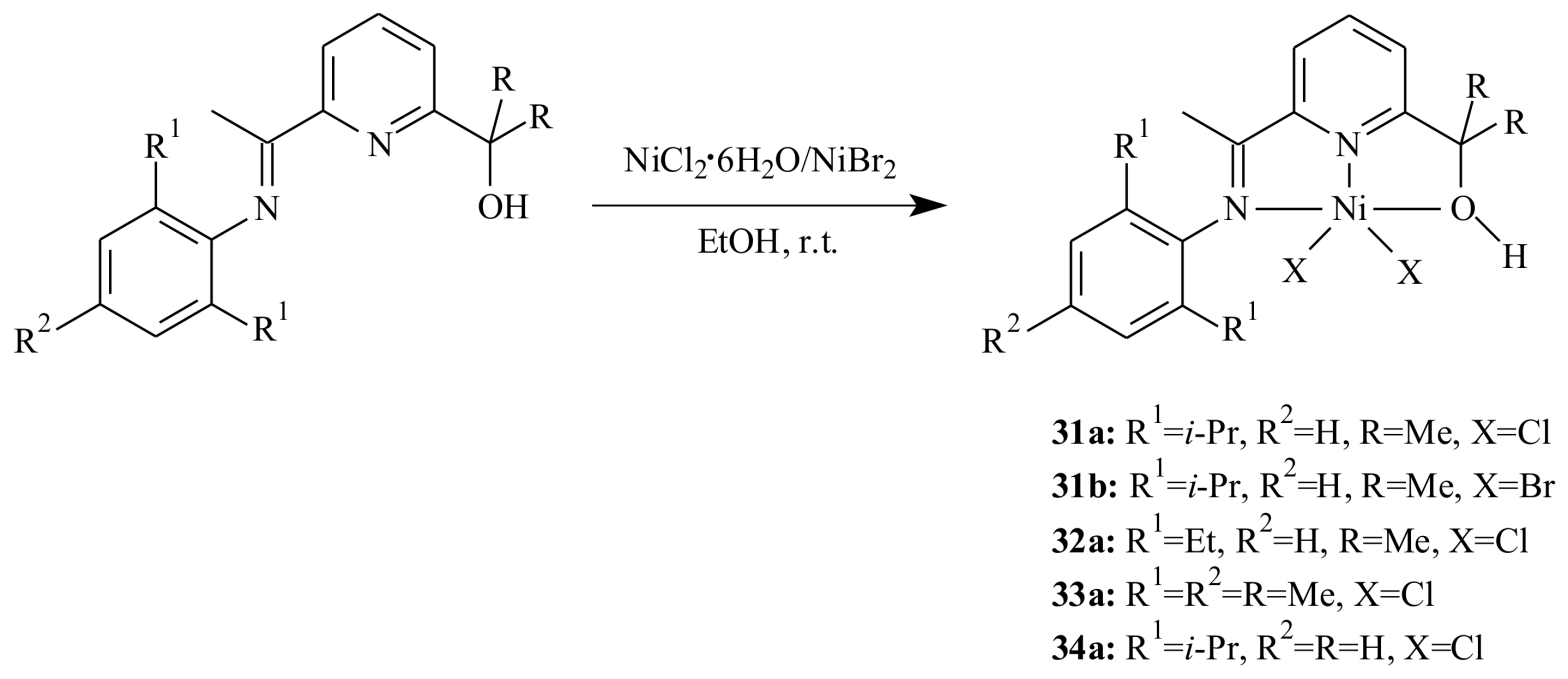
Synthesis of (imino)pyridinyl alcoholato complexes of Ni.

Vinyl-type norbornene polymers (PNBs) with high molecular weight and relatively narrow molecular weight distributions resulted from the polymerization of norbornene with Ni and Pd complexes bearing (imino)pyridinyl alcoholato tridentate ligands [43]. The five- and six-coordinated Ni complexes, and cationic Pd complexes formed with the [PdCl_4_]^2−^ counter ion were activated with excess methylaluminoxane (MAO). The polymerization reaction was performed at an Al/catalyst ratio of 1000:1 at 30 °C in toluene. The complexes are synthesized by mixing the corresponding pyridinyl alcohol with one equivalent of NiCl_2_·6H_2_O, NiBr_2_ or Ni(OAc)_2_·4H_2_O (for **31c**) in ethanol at room temperature (Scheme 4). The reaction of pyridinyl alcohols and PdCl_2_ in ethanol at room temperature proceeded relatively slowly; therefore the preparation of palladium complexes **31d** and **32d** was requisite to be carried out at 60 °C for 3 h.

**Figure 8:**
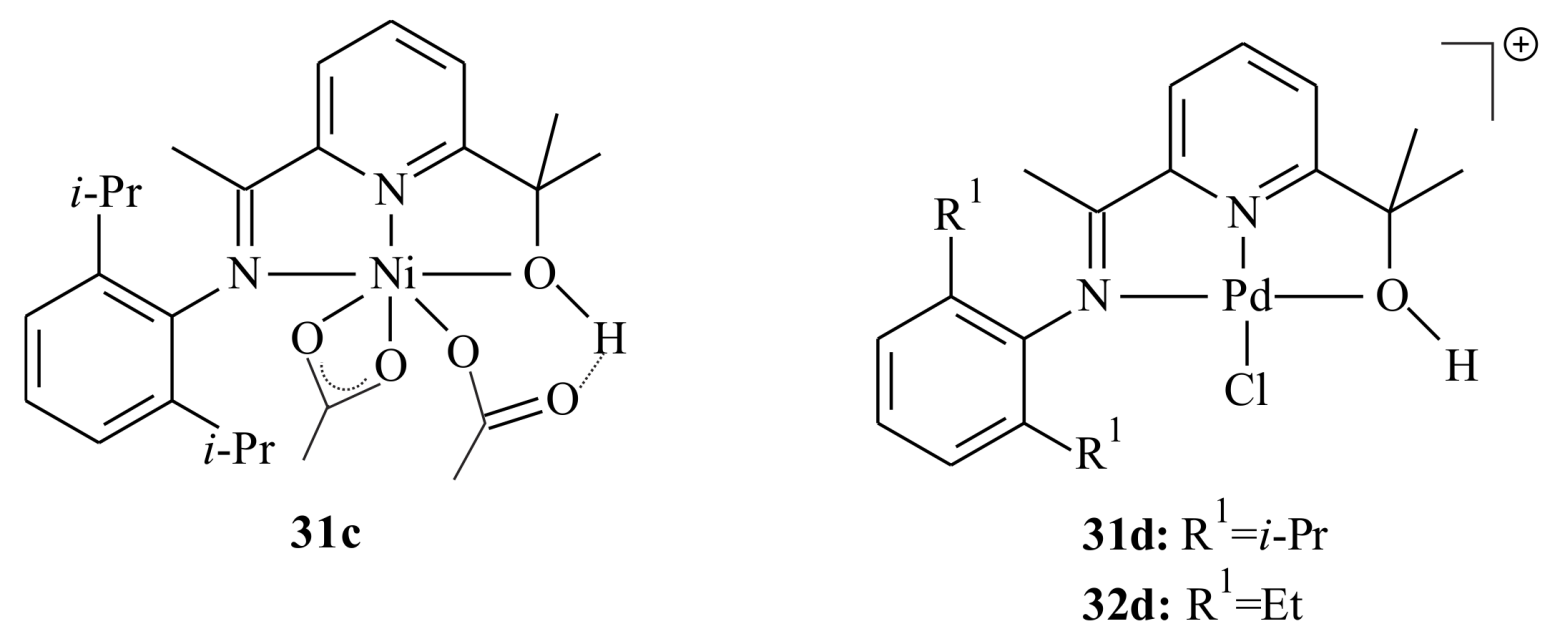
Structural differences between (imino)pyridinyl alcoholato Ni and Pd complexes.

The *N* and *O* atoms of the (imino)pyridinyl alcoholato ligand are coordinated to the metal centre with a distorted trigonal bipyramid for the nickel chloride complex **31a**, a distorted octahedron for the nickel acetate complex **31c** and a square planar arrangement for the cation moiety of palladium complex **32d** (see Figure 8).

**Scheme 5:**
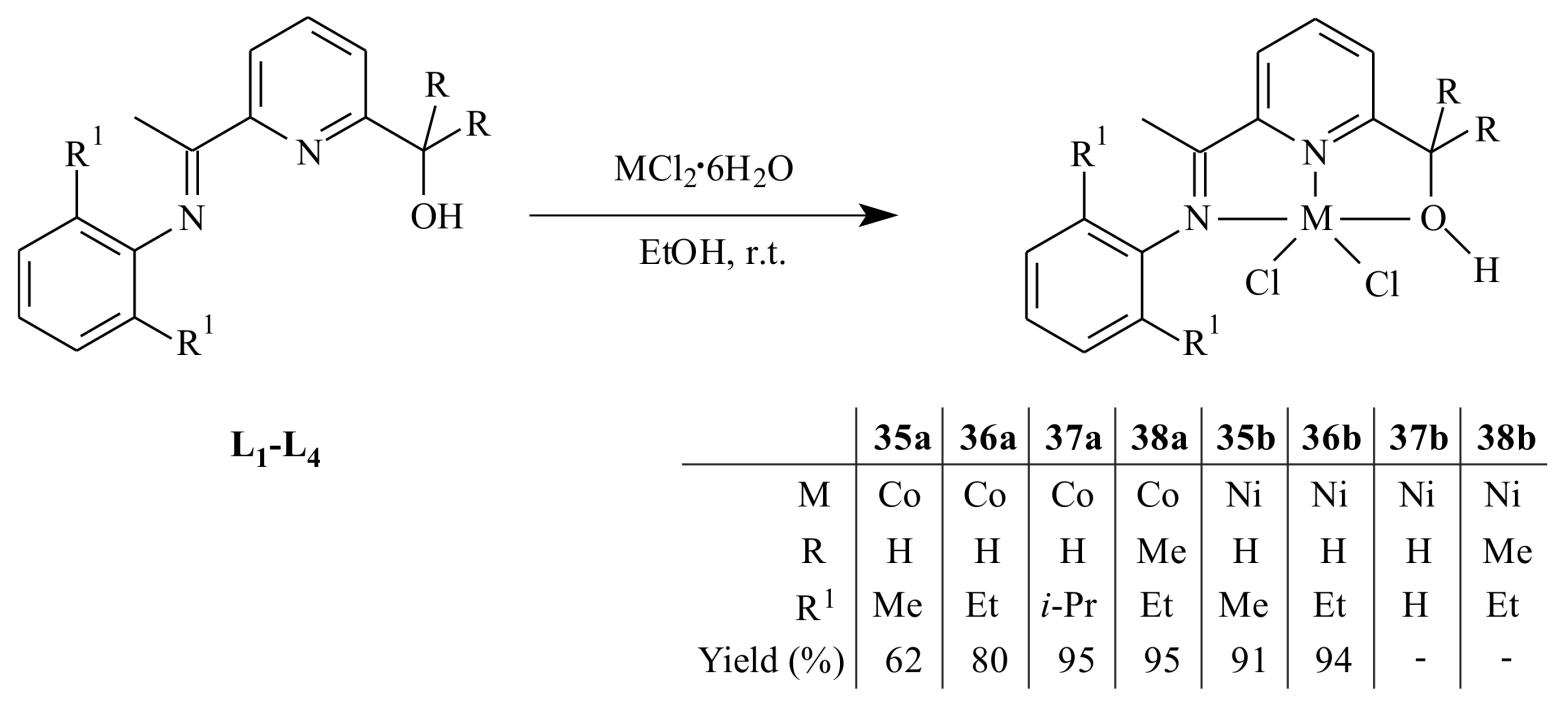
Synthesis of (imino)pyridinyl alcoholato complexes of Co and Ni.

**Scheme 6:**
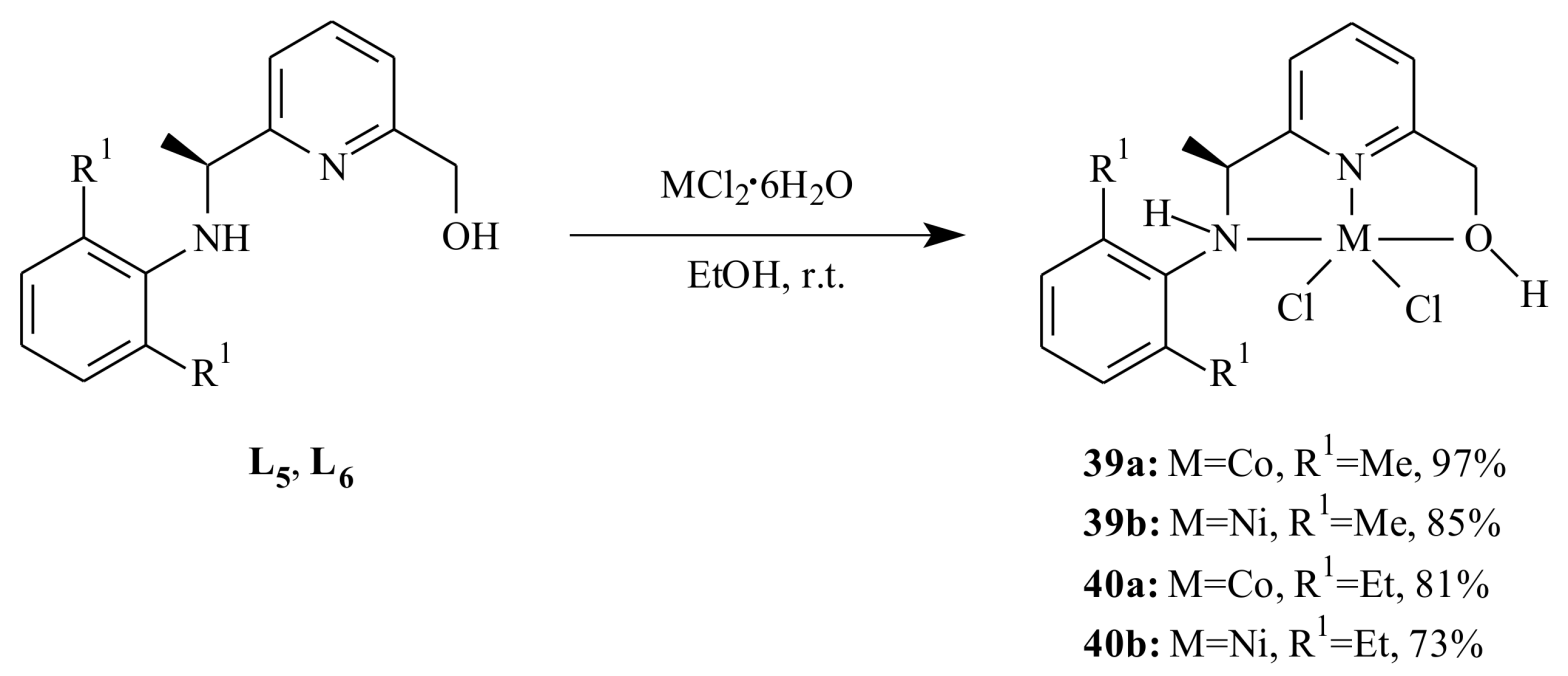
Synthesis of (amino)pyridinyl alcoholato complexes of Co and Ni.

A series of cobalt(II) and nickel(II) complexes **35**–**40** supported by (imino)- and (amino)pyridinyl alcoholato ligands were synthesized by Ai et al. [44] as it is shown in Scheme 5 and Scheme 6 and tested for 1,3-butadiene polymerization reaction. The 2-arylimino-6-(alcohol) pyridines (**L_1_**–**L_4_**) or 2-arylamino-6-(alcohol) pyridines (**L_5_** and **L_6_**) reacted with one equivalent of CoCl_2_·6H_2_O or NiCl_2_·6H_2_O in absolute ethanol at room temperature.

All the complexes adopted a distorted trigonal bipyramidal configuration with the equatorial plane formed by the pyridinyl nitrogen atom and two chlorine atoms. On activation with ethylaluminum sesquichloride (EASC), the cobalt complexes displayed high catalytic activity and selectivity (>96%) under Al/Co molar ratio of 40:1 at 25 °C. In comparison with the Co-complexes, the Ni-complexes resulted in relatively lower catalytic activity, *cis*-1,4 content and lower molecular weight under similar reaction conditions. The conversion of butadiene, microstructure and molecular weight of the resulting polymers were affected by the reaction parameters and size of substituents on the aromatic ring.

**Scheme 7:**
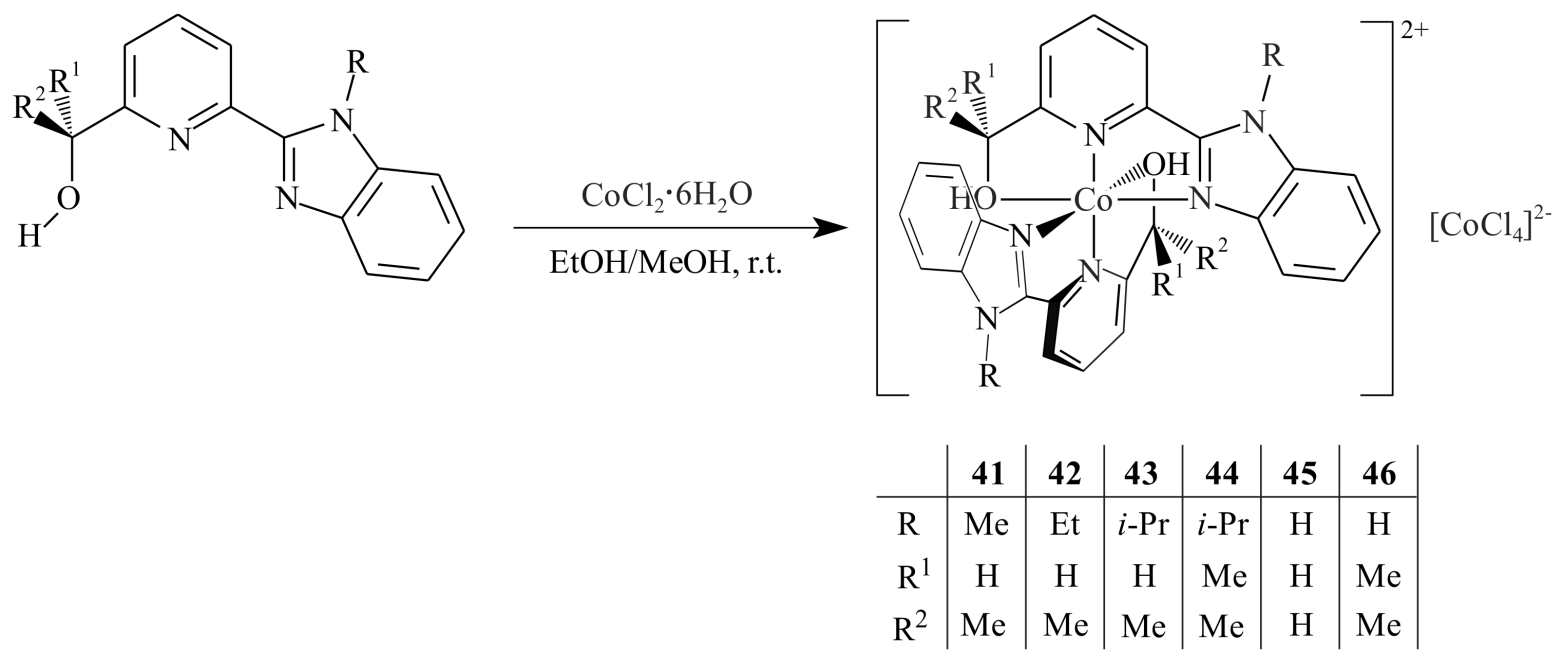
Synthesis of benzimidazolyl-pyridinyl alcoholato complexes of Co.

Ai et al. [45] synthesized a series of ion-pair cobalt complexes **41**–**46** supported by benzimidazolyl-pyridinyl alcoholato ligands and tested them in the polymerization of 1,3-butadiene (BD). The polymerization of BD by a pyridinyl alcoholato ligand supported cobalt catalyst resulted in *cis*-1,4-polybutadiene with high selectivity (>95%) under Al/catalyst molar ration of 50:1 at 30 °C upon activation with the cocatalyst EASC. In 1 h, 91–100% conversion of BD/catalyst ratio of 2500:1 and toluene as solvent is reported. Increasing bulkiness of substituents in the ligand structure led to lower catalytic activity. The ion-pair cobalt complexes were synthesized by reacting the corresponding pyridinyl alcohols with one equivalent of CoCl_2_·6H_2_O at room temperature in anhydrous ethanol or methanol (Scheme 7). All complexes possessed cationic octahedral geometry bearing two pyridinyl alcoholato ligands with [CoCl_4_]^2−^ as the counter ion.

The only pyridinyl alcohols involved in the preparation of Zr, Ti, Hf, Co, Ni and Pd complexes for olefin polymerization catalysis are the ones we have presented. Compared to the large number and varieties of pyridinyl alcohols, a very limited investigation has been done so far. It is therefore worthwhile to consider more pyridinyl alcohols with different electronic and steric effects and further investigate this area.

Enantioselective reactions have been given a great deal of attention in the past few decades. Biologically important organic compounds have chiral centres that became a challenge for the synthetic organic chemist. The synthesis of chiral organic compounds therefore needs chiral catalysts at large. Many research groups invested their time in looking for the best type of chiral catalyst that can result in excellent chemical yield (c.y.) and enantiomeric excess (ee).

Chiral pyridinyl alcohols have a great deal of attention in the construction of chiral transition metal catalysts. Significant progress has been made in utilising pyridinyl alcohols as chiral ligands in the various asymmetric reactions. Hereunder, we present the use of pyridinyl alcohols as chiral ligands in the various homogeneous asymmetric transition metal catalysed reactions.

### 2.3. Olefin Epoxidation

There are many methods for the preparation of epoxides. The most extensive industrial method is the oxidation of alkenes in liquid phase using organic hydroperoxides in the presence of a catalyst [46]. Indeed, hydrocarbon-soluble organometallic compounds of the transition metals can be employed as homogeneous catalysts.

**Figure 9:**
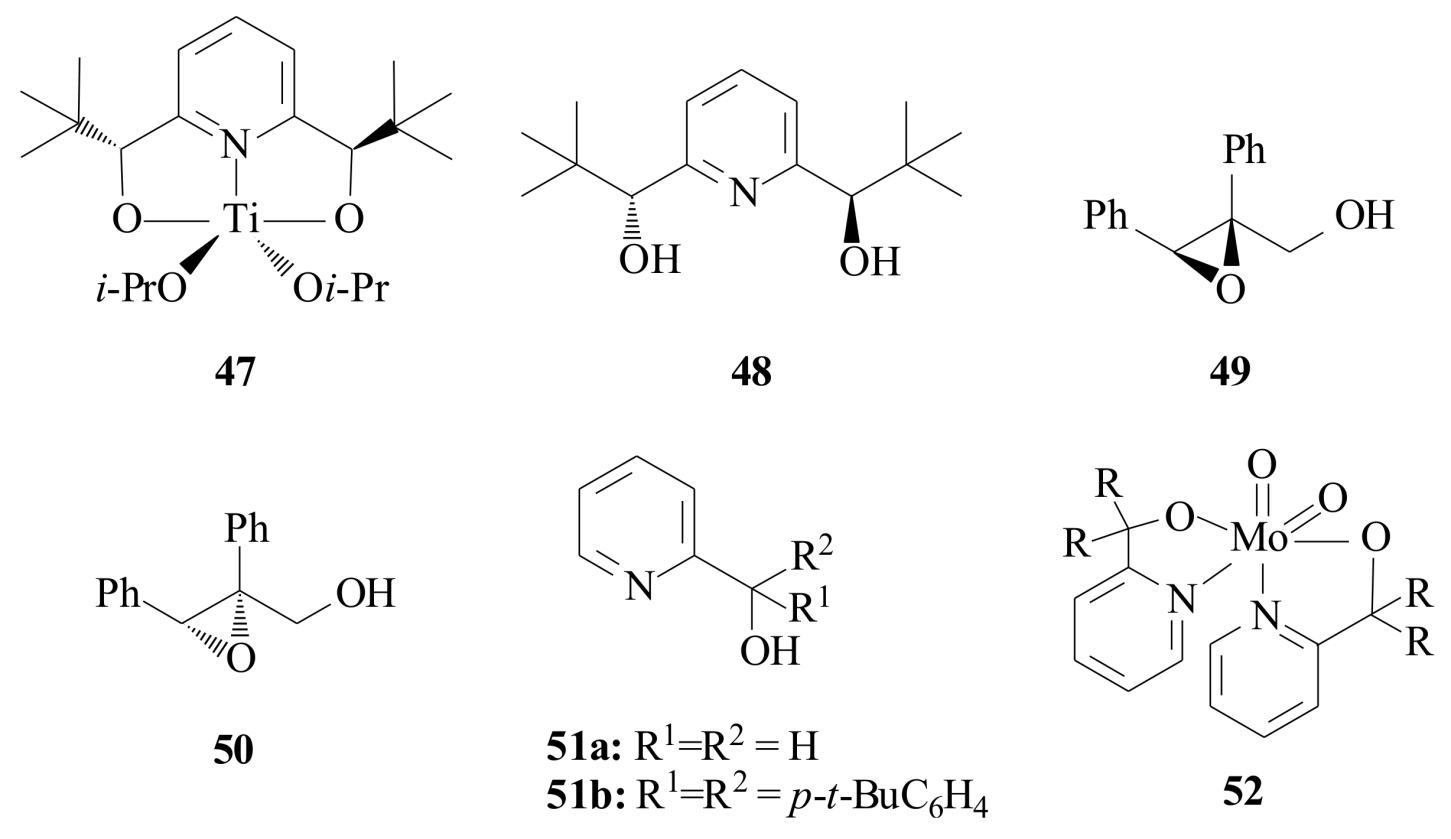
Ligands, complexes and epoxide products.

Hawkins and Sharpless [47] synthesized the pyridinyl diol **48** (Figure 9) for the purpose of testing the titanium(IV)-pyridine diol complex **47** as a model for titanium-tartrate asymmetric epoxidation catalyst. The model complex was synthesized by reacting **48** with Ti(O*i*-Pr)_4_ in CH_2_Cl_2_. The asymmetric epoxidation of (*E*)-*α*-phenylcinnamyl alcohol was catalysed by adding 3 moles of tertiary butylhydroperoxide (TBHP) to a mixture of X (X = 1.25, 1.50, 2.00) equivalents of **48** and Ti(O*i*-Pr)_4_, in CH_2_Cl_2_ at −5 °C. The epoxide **49** was obtained in 41% ee, 40% ee, and 41% ee, respectively (increasing the concentration of **48**). With the same procedure, 3 moles of tritylhydroperoxide (Ph_3_COOH) were added keeping the concentration of **48**, 1.5 equivalents and varying the concentration of Ti(O*i*-Pr)_4_, 3 mM, 13 mM, and 52 mM. The epoxide **50** was obtained in 64% ee, 64% ee and 52% ee, respectively.

The yield obtained with Ph_3_COOH, 64% ee agrees with the model complex **47** based on the titanium-tartrate system, while the 41% ee obtained with TBHP disagrees. This switch in face selectivity suggested an alkyl hydroperoxide dependent change in mechanism [47].

Tungsten(VI) compounds are well known for their catalytic activity in olefin oxidation reactions since Milas reported the metal-catalysed dihydroxylation of olefins with H_2_O_2_ and WO_3_ in the 1930s [48]. Herrmann et al. [28] came up with a result that revealed the nature of the ligand has an important influence on the product selectivity in addition to the metal oxidation state of the catalyst in terminal olefin epoxidation. During the investigation that was made to resolve the great challenges in the catalytic oxidation of terminal olefins to epoxides, by means of molecular oxygen, they synthesized pyridinyl alcoholato ligands **4** and **51a**,**b**. The dioxomolybdenum(VI) complex **52** was synthesized by reacting MoO_2_(acac)_2_ with two moles of the pyridinyl alcohol in MeOH, within 30 min at room temperature [28]. The geometry of complex **52** is distorted octahedral coordination geometry with the oxo-ligands forming a *cis*-dioxo unit that is common to all [MoO_2_]^2+^ complexes. While the nitrogen atoms of the pyridine are positioned *trans* to the oxo-ligand each, the alkoxy functions of the ligands are placed perpendicular to the plane defined by the dioxomolybdenum core. The solubility in non-polar aprotic solvents such as alkenes or alkanes and its stability during the course of the reaction make the complex valuable in catalytic applications. In the catalytic autoxidation reaction of 1-octene, they [28] obtained significant selectivity improvements (38 to 55%) with elemental oxygen. They also synthesized WOCl_3_L-type complexes via the treatment of WOCl_4_ with the corresponding pyridinyl alcohol in CH_3_Cl under mild conditions.

Two years later, Herrmann et al. [49] prepared dioxotungsten(VI) complexes of the type WO_2_L_2_ (**52**) with pyridinyl alcoholate ligands from WO_2_(acac)_2_, by ligand exchange, with two equivalents of pyridinyl alcohol in methanol. They synthesized the complexes dioxo *bis*[2-(2′-pyridinyl)propan-2-olato-*N*,*O*]tungsten(VI), dioxo *bis*{di[4″,4″-di(methoxy)phenyl]-(2′-pyridinyl) methanolato-*N,O*}tungsten(VI), dioxo *bis*[9-(2′-pyridinyl)fluoren-9-olato-*N*,*O*]tungsten(VI) and dioxo *bis*[5-(2′-pyridinyl)-10,11-dihydrodibenzo[a,d]cyclopentan-5-olato-*N,O*]tungsten(VI). The complexes were tested as catalysts (1 mol%) in olefin epoxidation with *tert*-butyl hydroperoxide (*t*-BOOH) (oxidant) and *cis*-cyclooctene (substrate) at 70 °C without solvent being in good (50–65%) conversion with excellent (100%) selectivity towards the epoxide was achieved.

**Figure 10:**
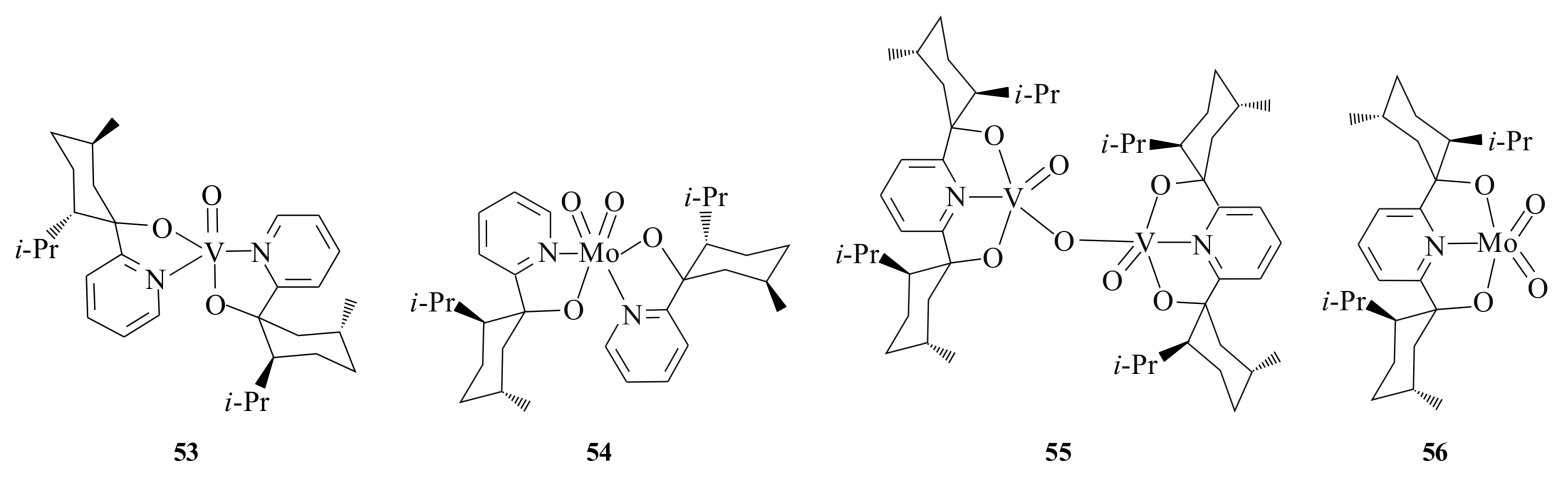
Multidentate catalysts for regioselective epoxidation.

Oxovanadium(IV) compounds as oxidation catalysts were first discovered by Katsuki and Sharpless [50] where they applied it in the regioselective epoxidation of allylic alcohols. The 2-[(−)-menthyl]-pyridine oxo-complexes of vanadium(IV), **53** and molybdenum(VI), **54** (Figure 10) were synthesized from VO(acac)_2_ and/or MoO_2_(acac)_2_ and 2-[(−)-menthyl]pyridine in 50% and 73% yield, respectively [51]. The vanadium(IV) complex is a square pyramidal and the Mo(VI) has a distorted octahedral arrangement. Both complexes were found to be active in the epoxidation of terminal olefins (e.g., 1-hexene 20% conversion and 25% ee) with *t*-BOOH as an oxidant. They also oxidised the sulphides (PhSMe) similarly to the corresponding sulphoxide (90% selectivity) with an ee of up to 18% using H_2_O_2_ as the oxidant.

Complexes **55** and **56** were prepared from the 2,6-*bis*[(1*S*,2*S*,5*R*)-(−)-menthyl]pyridine by treat ment with VO(O*i*-Pr)_3_ and/or MoO_2_(acac)_2_ respectively by Bellemin-Laponnaz et al. [52]. The complexes demonstrated the ability to catalyse the asymmetric oxidation of prochiral olefins with *t*-BOOH as the oxidant.

Chiral dioxomolybdenum(VI) and chiral dioxotungsten(VI) complexes of type **52** were also synthesized by Herrmann et al. [53] to investigate their catalytic potential in the asymmetric epoxidation of unfunctionalised olefins. The pyridinyl alcoholato ligands **57a**,**b** and **58** were used as ligands for the complex formation. Herrmann et al. [53] tested three different metal precursors bearing the *cis*-dioxo metal fragment for the formation of the complexes; (i) MoO_2_Cl_2_, 2-equivalents of lithium salt of ligand, THF, and refluxing for 30 min, (ii) MO_2_(acac)_2_, 2-equivalents of ligand, MeOH, 30 min, at room temperature, and (iii) Na_2_(MO_4_), 0.07-equivalent of ligand, H_2_O, pH 4, 12 h at room temperature. Although MoO_2_(acac)_2_ and WO_2_(acac)_2_ are much easier to handle than the dioxodichlorides (because of their high resistance to air and moisture), the metal salts Na_2_[MoO_4_] and Na_2_[WO_4_] are more advantageous as they are economical, easy to be handled, and ecologically friendly. In both complexes, a distorted octahedral coordination geometry was formed around the metal centres by the two anionic *N,O*-chelating ligands and two oxo-ligands [53].

Upon epoxidising *trans*-methyl styrene with the complexes **54**, the catalytic activity for molybdenum complexes resulted in good yields (71–81%) of the (*S*,*S*)-epoxide. Though the yields of tungsten complexes were only 31 to 36%, it is worthwhile to know the fact that tungsten systems were used for the first time in the asymmetric epoxidation of olefins. The bulkier the ligand, the higher the ee and complexes bearing the same type of ligand resulted in similar ee. Therefore, it is worthwhile to conclude that the enantioselectivity depends more strongly on the ligand than on the metal [53].

**Figure 11:**
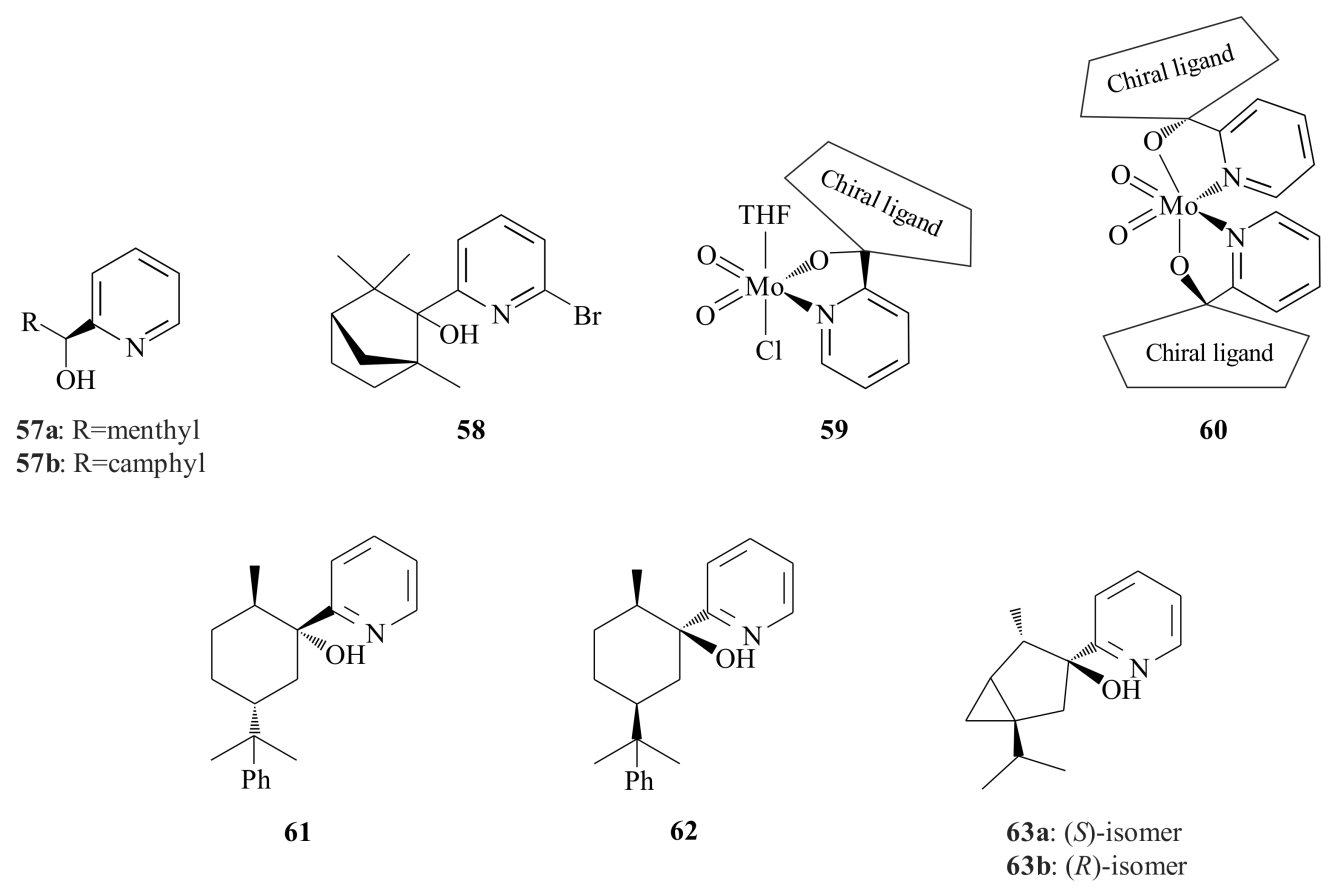
Chiral pyridinyl alcoholato ligands and Mo complexes.

Kuhn et al. [54] prepared chiral bis(oxazoline) and pyridinyl alcoholate dioxo-molybdenum(VI) complexes **59** (MoO_2_Cl(THF)L type) and **60** (MoO_2_L_2_ type) via treatment of MoO_2_Cl_2_(THF)_2_ with the corresponding 2-pyridinyl alcohol ligands (**57a**,**b**, **61**, **62**, and **63a**,**b**) in CH_2_Cl_2_ and/or with 2-equivalents of chiral pyridinyl ligands in TlOEt (method 1) or CH_2_Cl_2_ (method 2) respectively, in good yield (Figure 11). The catalytic potential of the complexes was evaluated in the asymmetric epoxidation of *trans*-*β*-methyl styrene using *t*-BOOH as oxidant. Conversions between 23–81% and ee 1–11% of the (*S*,*S*) configuration were observed in all cases except the complexes of the type MoO_2_Cl (THF)L, where L is pyridinyl alcohol made up of (+)-8-phenylisomenthone (**62**) and (−)-thujone (**63b**), which resulted in the (*R*,*R*)-configuration. Low substrate conversion (23–47%) and ee (1–5%) were observed for complexes of the type MoO_2_L_2_, **60** where the ligand is **57a**, **61** and **62**. Where L = **57b** has shown a substrate conversion of 51% and 23% ee (*S*,*S*)-configuration, which is the highest ee value.

The MoO_2_Cl_2_L (where L = 2-pyridinyl alcohol ligand) type complex **64** was synthesized via the reaction of MoO_2_Cl_2_(THF)_2_ with 2-pyridinyl alcohol in CH_2_Cl_2_. Using 2 moles of 2-pyridinyl alcohol in CH_2_Cl_2_ and refluxing resulted in **65** (MoO_2_L_2_ type) [55]. The (1*R*,2*S*,5*S*)-8-trimethyl silyloxy-1-(2-pyridinyl)menthol was prepared according to Herrmann et al. {28,53] and Kuhn et al. [54]. Complex **65** was tested for epoxidation of a variety of olefins using *t*-BOOH as oxidant at 55 °C under air atmosphere (1 atm) without solvent. The epoxidation results obtained were: cyclooctene (100% conversion and 89.8% ee), styrene (49.8% conversion and 54.0% ee), *α*-pinene (63.0% conversion and 11.7% ee) and (*R*)-(+)-limonene (89.5% conversion and 82.6% ee). The catalytic activity of **65** was slightly lower than **64**. Ring-opening activity was also observed for *α*-pinene oxide-producing campholenic aldehyde and epoxy campholenic aldehyde.

**Figure 12:**
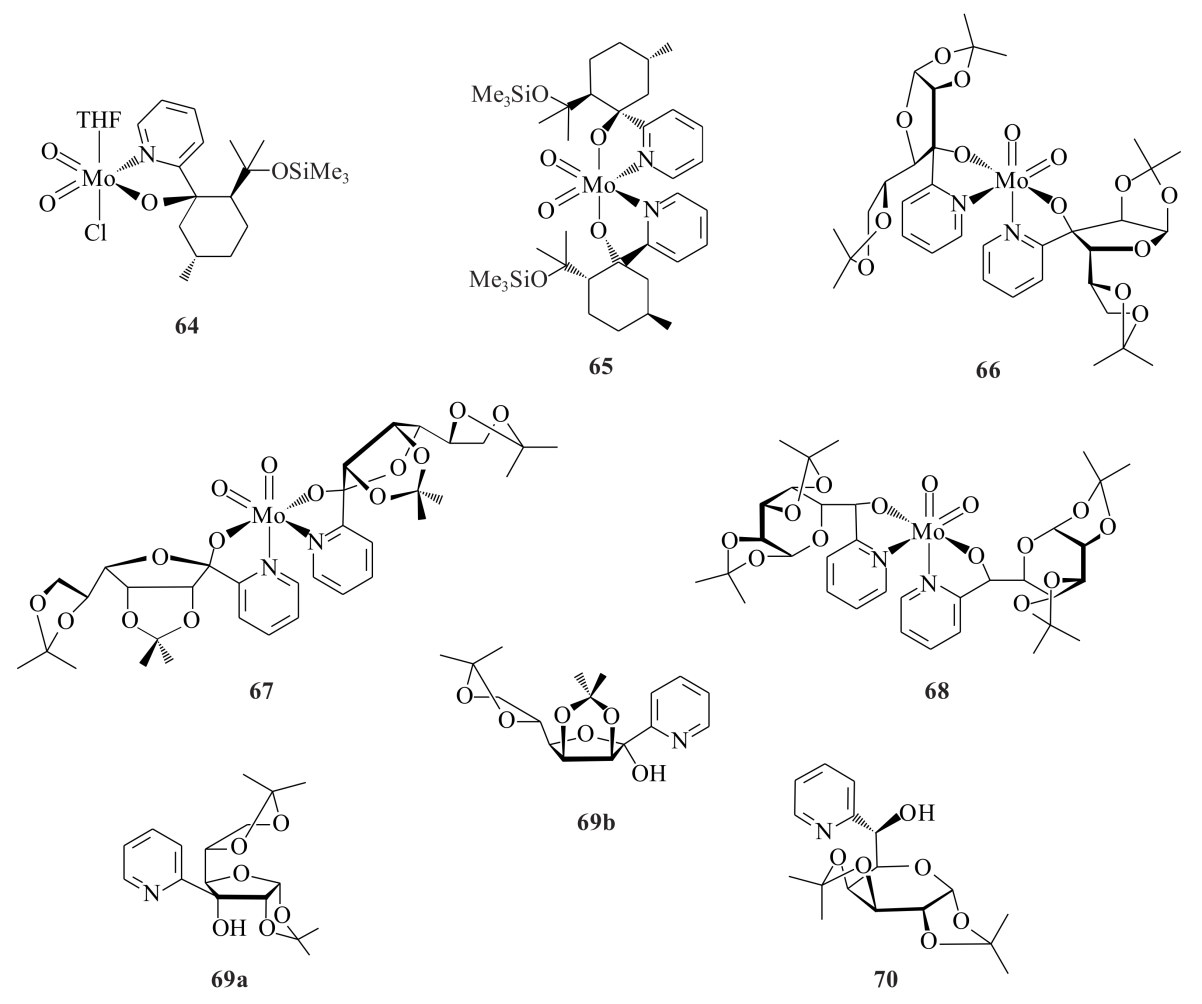
Mo complexes with chiral *N*,*O*-ligands.

Fridgen et al. [56] synthesized molybdenum(VI) complexes, **66**–**68** (Figure 12) with chiral *N*,*O*-ligands **69a**,**b** and **70**, which are derived from carbohydrate via the *n*-BuLi-mediated preparation method from 2-bromopyridine. Complex **66** was synthesized with its diastereomeric pair. The catalytic epoxidation of *trans*-methylstyrene with *t*-BuOOH or cumolhydroperoxide (CHP) in 1.0 mol% of catalysts **66**–**68** after 6 h at 50 or 70 °C was investigated. The results are shown in Table 2.

The influence of the oxidant can be seen at the enantiomeric excesses. With cumyl hydroperoxide, higher product selectivities were obtained, which might be a consequence of π-interactions between substrate and the oxidant. This type of interaction has also been taken into account for the Jacobsen and Cavallo system [57]. Similar ee values have been observed with a variety of *cis*-MoO_2_^2+^ epoxidation catalysts bearing chiral ligands, such as *bis*-oxazoline, *cis*-diol, *cis*-8-phenylthiomenthol, and others [54,58]. The complex *bis*[*N,O*-{2′-pyridinylmethanolate}]-dioxomolybdenum (VI) was synthesized and used as catalyst in the epoxidation of 1-octene with ethylbenzenehydroperoxide in liquid phase. Besides giving 1,2-epoxyoctane as the only product, the catalyst resulted in a better yield than its acetylacetonate complex [46].

**Figure 13:**
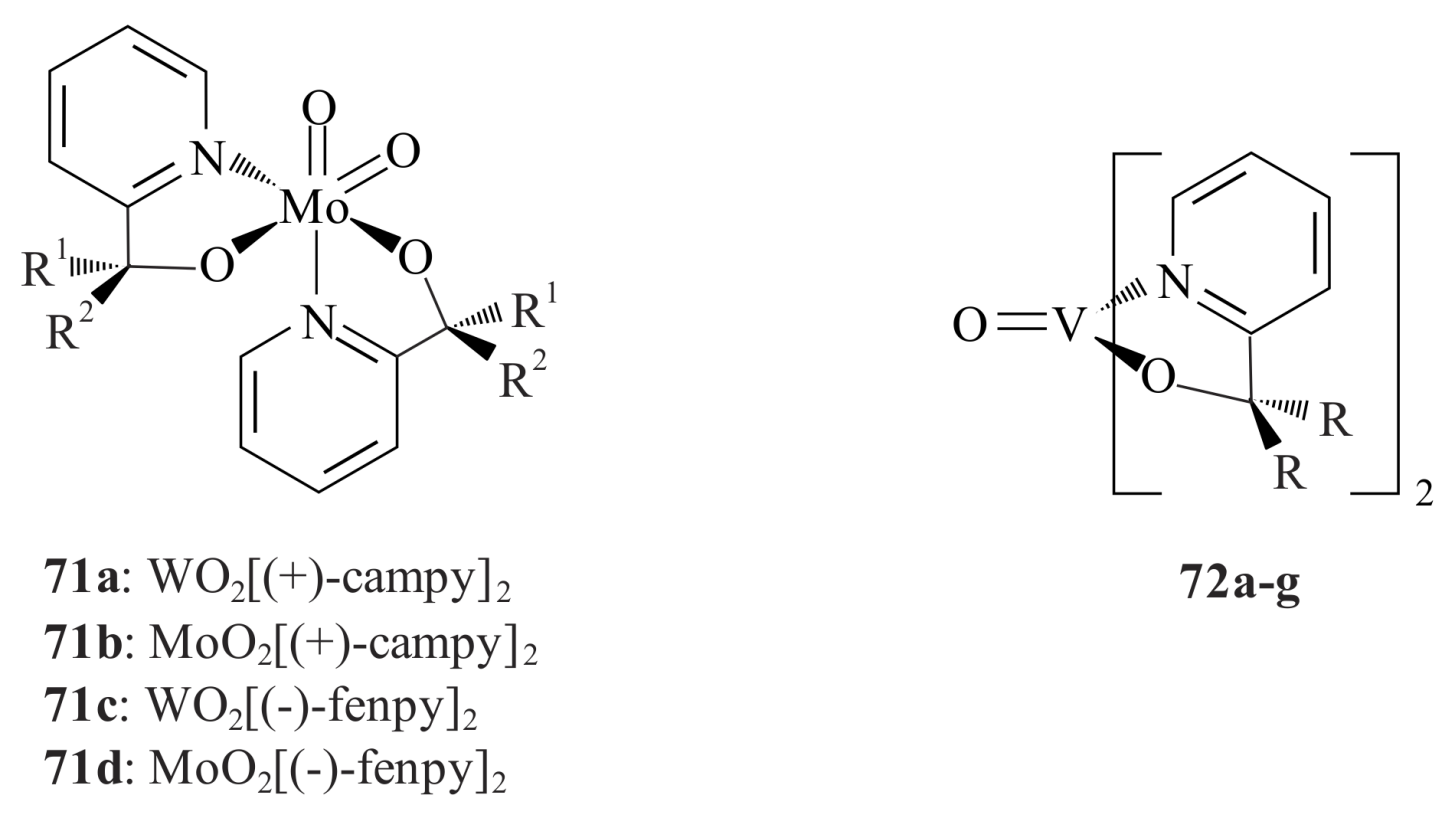
Chiral Mo and achiral V complexes.

Asymmetric epoxidation of *cis*-1-propenylphosphonic acid (CPPA) was catalysed by chiral tungsten(VI) and molybdenum(VI) complexes **71a**–**d** (Figure 13) [59]. The complexes were synthesized according to the methods of Herrmann et al. [53] and Kuhn et al. [54] The CPPA was converted to 100% of the corresponding epoxides with ee’s varying from 52 to 80% in CH_2_Cl_2_, which is the better solvent for this specific epoxidation, at reaction temperatures of 0, 25 or 50 °C. Generally, complexes with ligand (+)-campy (which is the pyridinyl alcohol from (+)-camphor) resulted in slightly higher ee’s than (−)-fenpy (the pyridinyl alcohol from (−)-fenchone). Similar product ee’s were observed in the presence of catalysts with similar ligands but different metals. At 0 °C in CH_2_Cl_2_, complex **71b** catalysed the epoxidation reaction to give the product with the highest ee value of 80%. At 50 °C, the ee of the epoxide was reduced to 68% with the same catalyst. The epoxidation mechanism was described as direct oxygen transfer on the interface of the biphasic H_2_O-nonprotic system.

Except for VO(acac)_2_, oxovanadium compounds have never been employed in oxidation catalysis with molecular oxygen. Oxovanadium(IV) complexes of *bis*(aryl)-2-pyridinyl alcohols **72a**–**g** (Figure 13) where the ‘R’ is phenyl **72a** (73%), *p*-(*t*-Bu)-C_6_H_4_ **72b** (77%), *p*-F-C_6_H_4_ **72c** (71%), C_12_H_8_(cyclic) **72d** (76%), *m*-CF_3_-C_6_H_4_ **72e** (35%), *p*-Cl-C_6_H_4_ **72f** (78%) and *p*-MeO-C_6_H_4_ **72g** (68%) were synthesized by Lobmaier et al. [60] The ligands were prepared by a nucleophilic attack of 2-lithiopyridine on the corresponding aromatic ketones according to reported procedures [61,62,63,64]. The catalysts were synthesized via refluxing VO(SO_4_)·5H_2_O with 2 moles of the pyridinyl alcohol in the presence of CH_3_CO_2_Na in EtOH. The complex is a square planar pyramid coordination sphere of vanadium with the nitrogens in the pyridine rings *trans* to each other. Autoxidation reaction with 1-octene and molecular oxygen resulted in selectivity of approximately 30%, which showed slight improvement from the reaction without any catalyst (23%).

**Figure 14:**
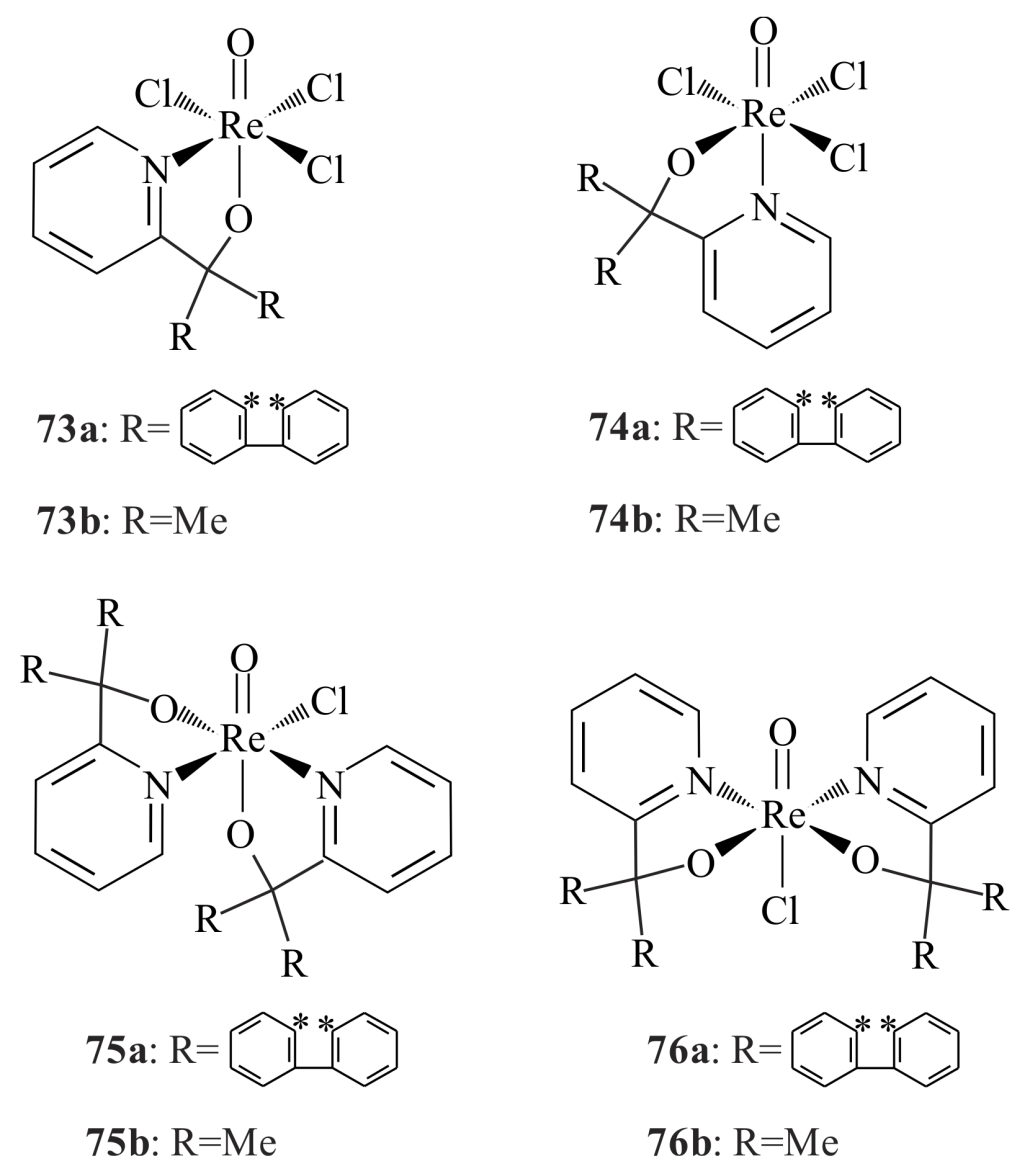
Pyridinyl alcoholato complexes of Re.

The rhenium pyridinyl alkoxide complexes of the type [ReOCl_3_(L)(NBu_4_) and [ReOCl(L)_2_] (where L = pyridinyl alcohol ligand), **73**–**76** (Figure 14) were synthesized by Lobmaier et al. [65] The complexes **73a**,**b** and **74a**,**b** were synthesized via reacting ReOCl_4_^−^[NBu_4_]^+^ with the corresponding pyridinyl alcohol in THF at room temperature for one hour. Heating **73a**,**b** also resulted in **74a**,**b**, which therefore tells us that complexes **73a**,**b** are the kinetic, whereas **74a**,**b** are the thermodynamic products. They also used another method to synthesise complexes **74a**,**b**, i.e., by reacting ReOCl_4_^-^[NBu_4_]^+^ with 2 moles of the pyridinyl alcohols (with R = CH_2_CH_3_, (CH_2_)_2_CH_3_ and Ph) in EtOH at room temperature for three hours. This method is more convenient than the previous one because there is no need for LiCl extraction as the second mole of the pyridinyl alcohol forms a pyridinium chloride salt. It was possible to see the octahedral coordination environment of rhenium with substantial distortion from the single crystal X-ray crystallography of **73**. The co-planarity of all chlorines and the *trans* coordination of the pyridine ligand to the oxo-ligand of the rhenium are also vividly seen in the X-ray crystallography. Complexes **75a**,**b** were prepared via reaction of ReOCl_4_^−^[NBu_4_]^+^ with 4 moles of the pyridinyl alcohol in EtOH at 80 °C for one hour. Complexes **76a**,**b** were obtained while preparing complex **75a**,**b**. Palladium (II) and copper (II) complexes of the various pyridinyl alcohols were also synthesized by Lobmaier et al. [65]; however, as they are inactive towards the epoxidation reaction, we have left them without consideration. The complexes **73a**,**b** and **74a**,**b** partially decomposed during testing epoxidation of cyclooctene in TBHP. The complexes **75a**,**b**, on the other hand, have shown significant catalytic activity in the formation of cyclooctene oxide during the first eight hours.

The epoxidative reactions of both terminal and internal, open chain and cyclic olefins using the common oxidants *t*-BOOH, H_2_O_2_ and TBPH were well investigated by several research groups; the results in both conversion and selectivity were being promising. Herrmann et al.’s [29] and Lobmaier et al.’s [60] investigation into the alleviation of the problem pertaining to the oxidation of terminal olefins by molecular oxygen is a good beginning. However, the results obtained are much less both in conversion and selectivity compared to that of epoxidation with oxidants. Therefore, it needs further investigation in order to come up with a better conversion and selectivity.

### 2.4. Cyclopropanation

**Figure 15:**
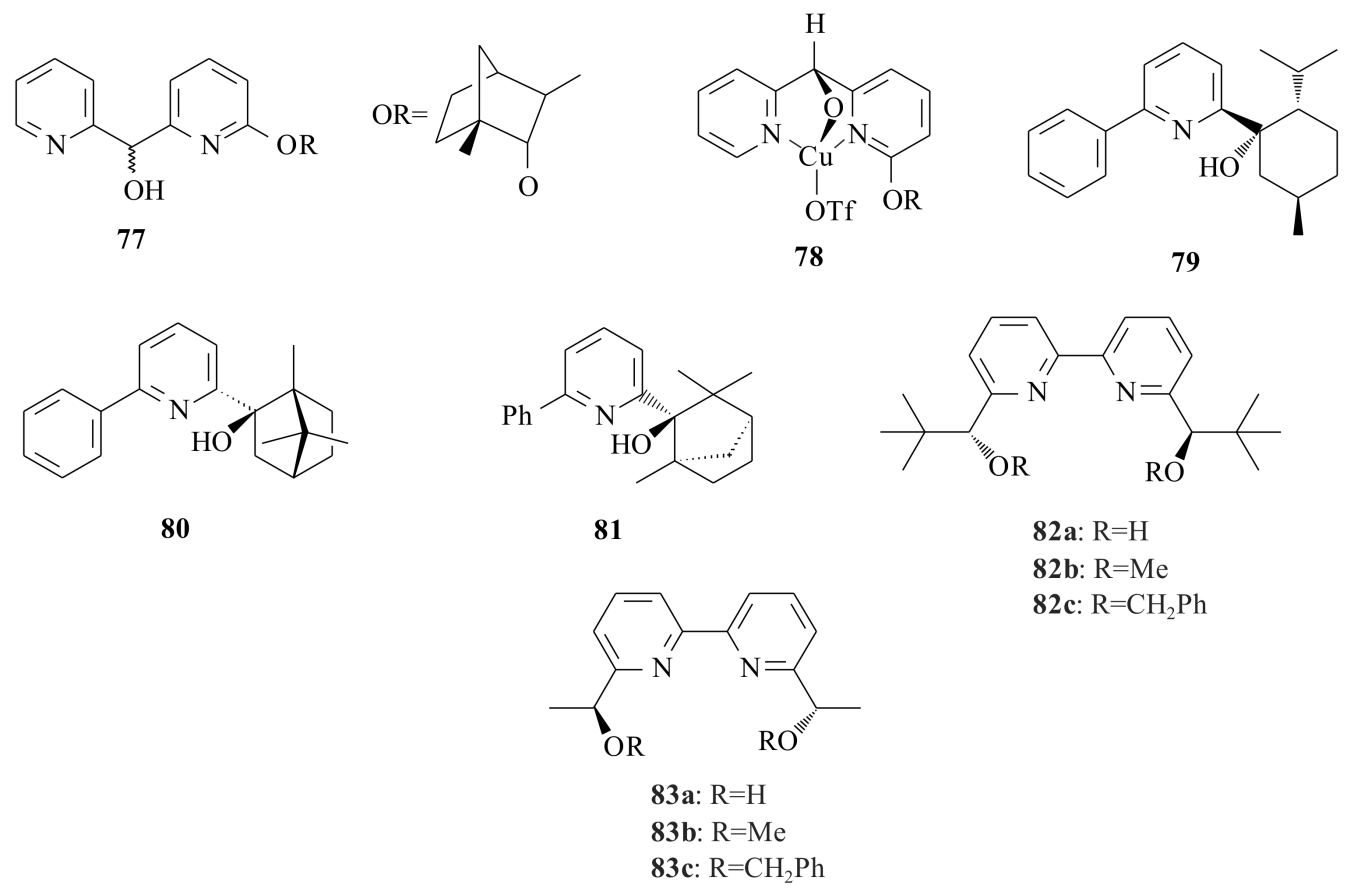
Ligands and typical Cu complex used for cyclopropanation.

The least number of naturally occurring organic molecules contain the cyclopropane ring as part of their structure. The cyclopropane ring is reactive toward ring opening and rearrangements due to special bonding properties. Cyclopropanation is therefore a very valuable synthetic reaction. According to Charette et al. [66], cyclopropanation reactions can be categorised into (i) halomethyl metal-mediated cyclopropanation reactions, (ii) transition metal catalysed decomposition of diazo compounds, and (iii) nucleophilic addition-ring closure sequence. In this section, we present the synthesis of transition metal catalysts that contain pyridinyl alcoholato ligands and their catalytic activities in the cyclopropanation reactions. The racemic mixture of ligand **77** was treated first with BuLi at −78 °C in THF and then with Cu(CF_3_SO_3_)_2_ to result in the copper(II) catalyst (**78**) (see Figure 15). Treatment of 2,5-dimethyl-2,4-hexadiene with ethyl diazoacetate (EDA) in the presence of **78** resulted in 8.5–10% ee of the cyclopropanated product. The configuration for the bridging carbon atom in ligand **77** controls the chirality of the product and this suggests that, in the cyclopropanation reaction, a lack of *C*_2_ symmetry for the ligand is a desirable feature [67].

The copper(II) complex prepared from the methoxide of the bipyridinyl alcohol **48** was found to be active for the asymmetric cyclopropanation of alkenes resulting in ee up to 92% with good c.y. (78%) [68]. Synthesis of Cu(II) and Cu(I) complexes of pyridinyl alcoholate ligands (**57a**,**b**, **58**, **79**, **80**, and **81**) and their catalytic application in asymmetric cyclopropanation of styrene with EDA were reported by Lee et al. [69] (see Figure 15). Complexes of Cu(L)_2_ (where L = pyridinyl alcohols **57a** and **58**) were synthesized via reacting Cu(OAc)_2_ with 2 moles of the pyridinyl ligand in MeOH/EtOH (1:1) resulting in 61% (L = **57a**) and 76% (L = **58**). The Cu(II) complex with the pyridinyl ligand **57b** was prepared upon reacting the pyridinyl alcohol with NaH in THF followed by Cu(OAc)_2_ treatment, which resulted in 73% yield.

The X-ray crystallographic data shows that the copper(II) centres for both Cu(**57a**)_2_ and Cu(**58**)_2_ complexes are four-coordinate and have a square planar geometry surrounded by two nitrogen and two oxygen atoms of the two ligands. The *N* and *O* atoms of the two ligands are *trans* to each other. The sum of the bond angles around the Cu(I) centre for the two complexes equals 360.1° and 360°, respectively, and suggested that the complexes are planar. Cyclopropanation of styrene with ethyl diazoacetate in the presence of Cu(**57b**)_2_ resulted in 65% c.y. with 6% (1*S*,2*S*)-*trans* and 22% (1*S*,2*R*)-*cis,* which is a reasonable yield. However, addition of triflic acid in a 1:1 ratio to the complex led to an active copper catalyst in 98% c.y. with 31% (1*S*,2*S*) and 40% (1*S*,2*R*). The dissociation of the coordinated ligand and creation of a vacant site in the coordination environment is believed to be the reason for the activity improvement upon addition of the triflic acid. The complex [Cu(L)]^+^ can also be generated in situ by reacting Cu(II) triflate with ligands **57a**,**b**, and **58** in CH_2_Cl_2_. The yields of the isolated cyclopropane esters obtained from in situ generated complexes were good and ranged from 67 to 87% with enantioselectivities between 3 and 53%. The pyridinyl alcohol **80** gave 51% *trans* and 53% *cis* product.

Chiral copper-bipyridinyl complexes of the type Cu(L)Cl_2_ (where L = pyridinyl alcohol ligand) were synthesized from the bipyridinyl alcohols **82a** and **83a** [70] and their corresponding alkoxy ethers **82b**,**c**, **83b**,**c** [70] by Lee et al. [71] (see Figure 15) Kinetic and mechanistic studies suggested that 14-electron species are the active catalyst in the carbene transfer reaction. The TfO^−^, PF_6_^−^ BF_4_^−^ and SbF_6_^-^ forms of the complex function as catalysts in the asymmetric cyclopropanation of styrene with EDA. Complexes with sterically bulky group ligands are more easily reduced than those ligands with less bulky groups.

Little investigation with a limited type and number of pyridinyl alcohols has been reported in the cyclopropanation catalysis development. It is therefore important to increase the scope of the pyridinyl alcohols and further investigate the carbene transfer reaction in detail in order to come up with a better chemical yield and excellent enantioselectivity.

### 2.5. Addition of Organozinc Compounds to Chalcones

Addition reactions of organozinc compounds to *α,β*-unsaturated carbonyls are one of the crucial reactions through which C-C bond formation occurs. Upon using chiral catalysts, it is possible to obtain a *β*-substituted chiral carbonyl compound. The chiral pyridinyl and bipyridinyl alcohols are used as chiral auxiliaries in this reaction for better selectivities on the desired configuration of the product. Therefore, we present the results of various investigations that have been done so far.

**Scheme 8:**
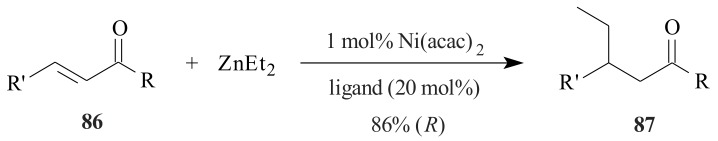
Nickel-catalysed conjugate addition of Et_2_Zn to chalcones.

Pyridinyl alcoholato ligands **84** and **85a** were used by Bolm [72] in the asymmetric amplification of nickel-catalysed conjugate addition to chalcone **86** (R and R’ = Ph). The asymmetric amplification factor (quotient of product ee/ligand ee) increases with an increase in the concentration of ligand **84** keeping the metal concentration constant for the reaction shown in Scheme 8. The use of the pyridinyl alcoholato-ligand improved the enantioselectivity of the catalyst [72].

**Figure 16:**
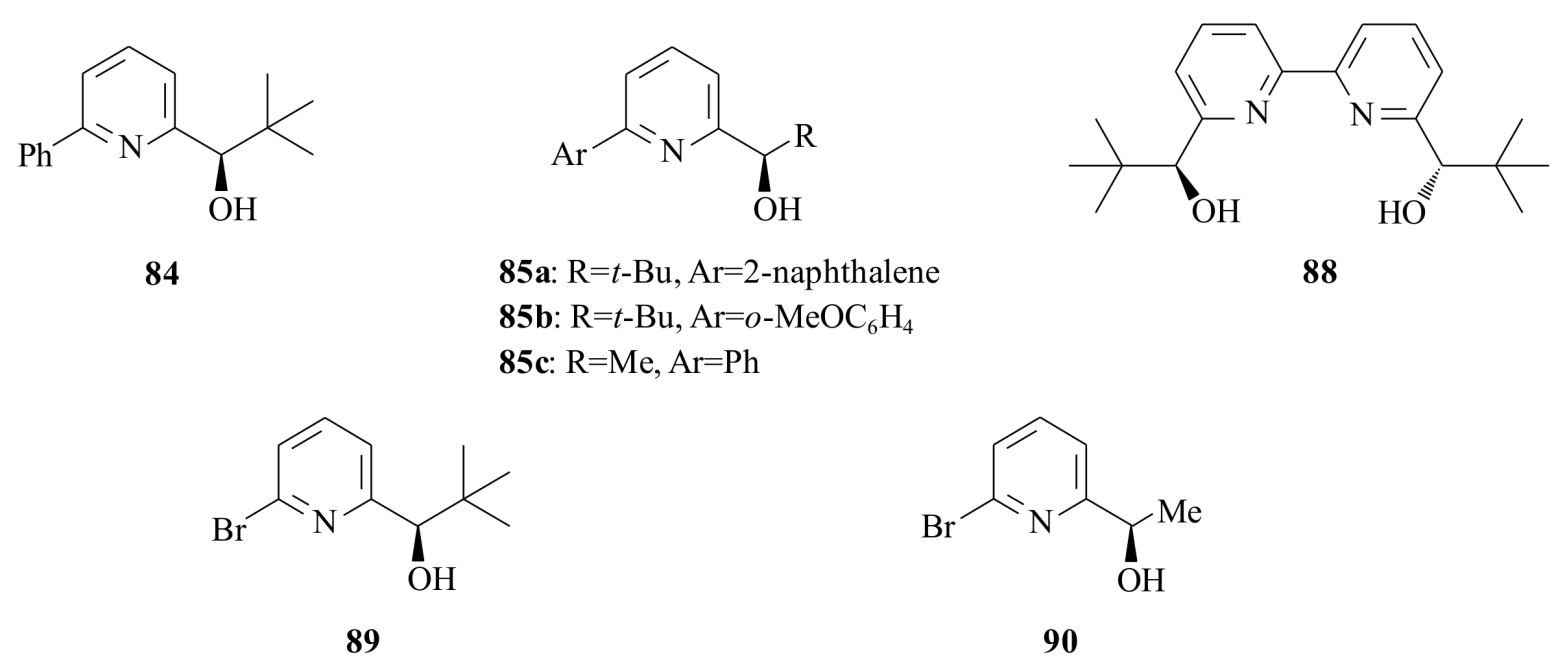
Pyridinyl alcoholato ligands used for asymmetric additions to chalcones.

The bipyridinyl diol **88** was used as an efficient catalyst by Bolm and Ewald [73] for the same reaction as the above. They applied the catalytic reaction for; a) R and R’ = Ph, b) R = *p*-CH_3_OC_6_H_4_, R’ = Ph, c) R = CH_3_, R’ = Ph. The product yield and ee depended on the ratio of Ni:**88**. Best yield (75%) and ee (72%) were obtained for 1:20 mol% of Ni:**88** (in a). However, increasing the amount of **88** to 1:30 mol% decreased the yield to 55%, while the ee% remained 72% (in a). In the opposite sense, decreasing the amount of **88** to 1:10 mol% increased the yield to 82%, but the ee decreased to 54% (in a). 1:10 mol% of Ni:**88** resulted in 86% yield and 74% ee (in b). In the case of ‘c’ (1:5 mol%), a racemic mixture of 76% yield was obtained. The recovery of **88** by column chromatography, without loss of optical purity, was the other advantage of the ligand. Bolm and co-workers [74,75] utilised pyridinyl alcohol ligands **84**, **85a**–**c**, **89**, and **90** (Figure 16) to investigate the effect of variation of ligand structure on the product yield and ee. They also used ligands **84** and **88** to study the effect of concentration of Ni and ligand on the enantiomeric excess of the product for the same catalytic enantioselective reaction as the above (see Table 3 and Table 4). In Table 3, entries 2 and 10’s highest yield and ee were obtained by using a ligand concentration of 20 mol% in both cases. A ligand concentration of 30 mol%, on the other hand, resulted in high ee, but with relatively low yield (entries 1 and 9).

The impact on the ee of the absence of bulky groups in the chiral position of the pyridinyl alcohols **85c** and **90** was seen clearly in Table 4. Varieties of solvents were investigated for the above reaction and acetonitrile was found to be the one that resulted with best yields. An investigation of the impact of reaction time on the ee of the product revealed that increasing the time (from 0.25 h to 18 h) has shown a decrease in ee from 88% to 51% using ligand **84**.

**Scheme 9:**
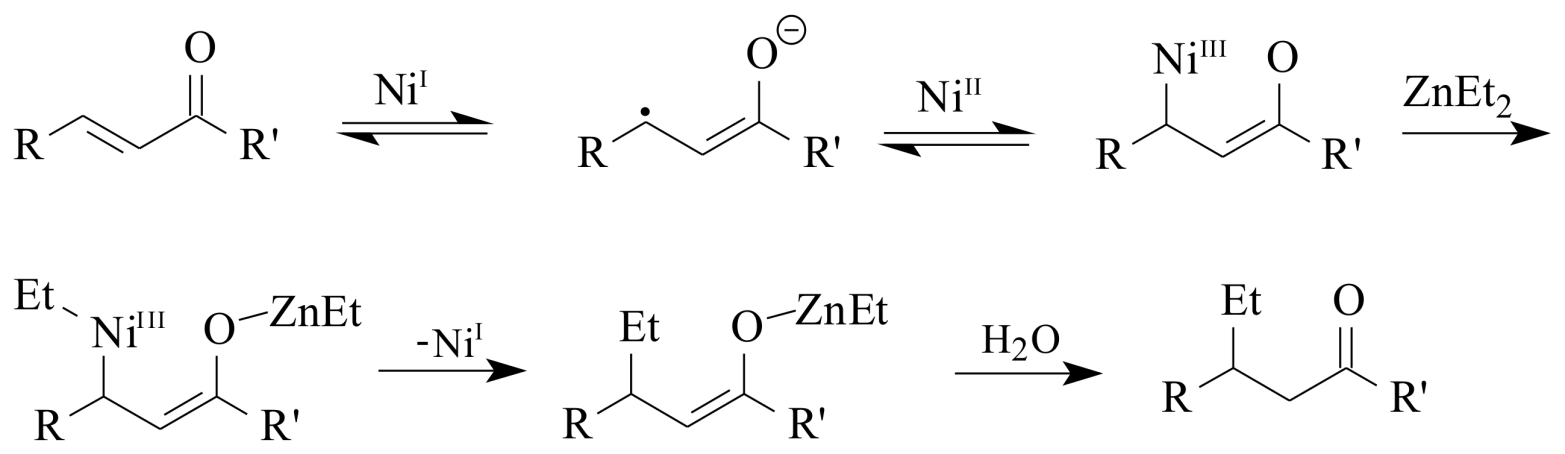
Mechanism of Ni-catalysed conjugate addition of Et_2_Zn to chalcones.

The suggested mechanism for the above reaction by Bolm and Ewald [74] involves electron transfer changes in the oxidation state from Ni^II^ to Ni^I^ and Ni^0^. This happens in such a way that the organozinc reagent is used to reduce Ni(acac)_2_ to a catalytically-active nickel(I) species. Electron transfer from Ni^I^ to the substrate generates a ketyl radical, which reacts with the resulting Ni^II^ species. Transmetallation followed by reductive elimination gives the zinc enolate and regenerates the chirally-active nickel(I) species (see Scheme 9).

**Figure 17:**
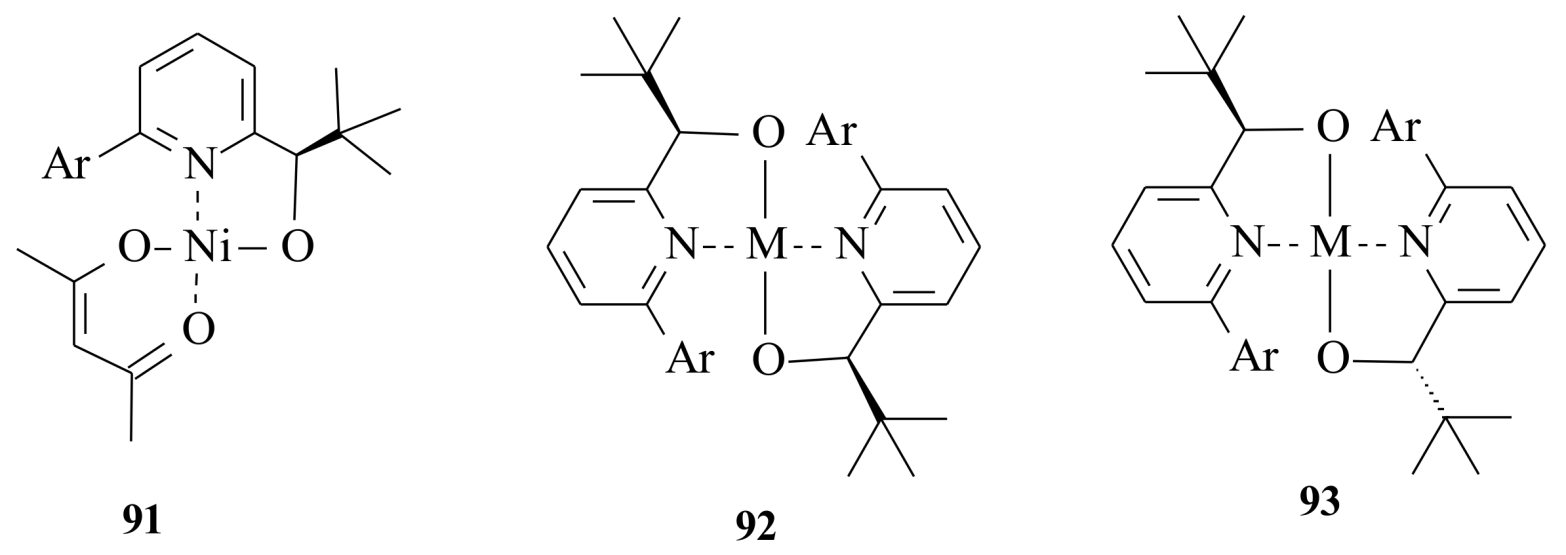
Intermediates proposed for the Ni-catalyzed addition reactions [74,75].

According to the above mechanism, the asymmetric induction is dictated by an enantioselective formation of the Ni^III^ intermediate followed by a stereoselective reductive elimination. Based on the above mechanism, they [74,75] proposed the intermediates **91**–**93** (not synthesized) in Figure 17.

The enantioselectivity of the ligand revealed the fact that bulky groups on the chiral position of the pyridinyl alcohol result in high enantioselectivities. However, there still remains further investigations to be done before reaching this conclusion as there are many pyridinyl alcohols that vary structurally. It is necessary to investigate pyridinyl alcohols that are substituted other than the positions C-6 in the pyridinyl alcohol. It would be more comprehensive if electronic effects are also considered in the pyridinyl alcohols.

### 2.6. Enantioselective Addition of Organozinc Compounds to Aldehydes

Reactions of organozinc compounds to aldehydes follow a nucleophilic addition pathway. The reaction served as one of the very important C-C bond formation reactions that results in chiral secondary alcohols of synthetic importance. The ability to control the enantioselectivity of the product is a value-adding reaction to the synthetic organic chemist. The literature survey of the various investigations that are done so far with special emphasis on the use of pyridinyl and bipyridinyl alcoholato ligands is presented hereunder.

Although dialkylzinc is not often utilised for chiral addition to aldehydes in modern organic synthesis, due to the rise of other fast reacting reagents such as alkyllithium and Grignard, it has served a great deal since its discovery by Frankland [76] in 1849. The use of a catalytic amount of (*S*)-leucinol with diethylzinc, which resulted in 49% ee, is the beginning of research on asymmetric organozinc additions to carbonyl compounds [77]. According to the reports in literature, coordination of ligands to dimethylzinc converts its linear structure into an approximate tetrahedral structure [78]. This reduces the bond order of the Zn-C bond and increases the nucleophilicity of the zinc alkyl groups. Consequently, chiral ligands not only control the stereochemistry of the organozinc addition, but also activate the zinc reagents.

In organozinc addition to carbonyls, the stereochemical outcome of the reaction was significantly influenced by the aggregation behaviour of the various zinc-containing species that are involved in the transformation [79]. The pyridinyl alcohols react with dialkylzinc to produce zinc-based chiral Lewis acid complexes that can further coordinate with both aldehyde and substrates and the dialkylzinc reagent to conduct the catalytic addition. In addition to its action as a Lewis acid to activate carbonyls and also as Lewis base to activate the organozinc reagents, the chiral environment of the ligand controls the stereochemistry of the reaction [77].

**Figure 18:**
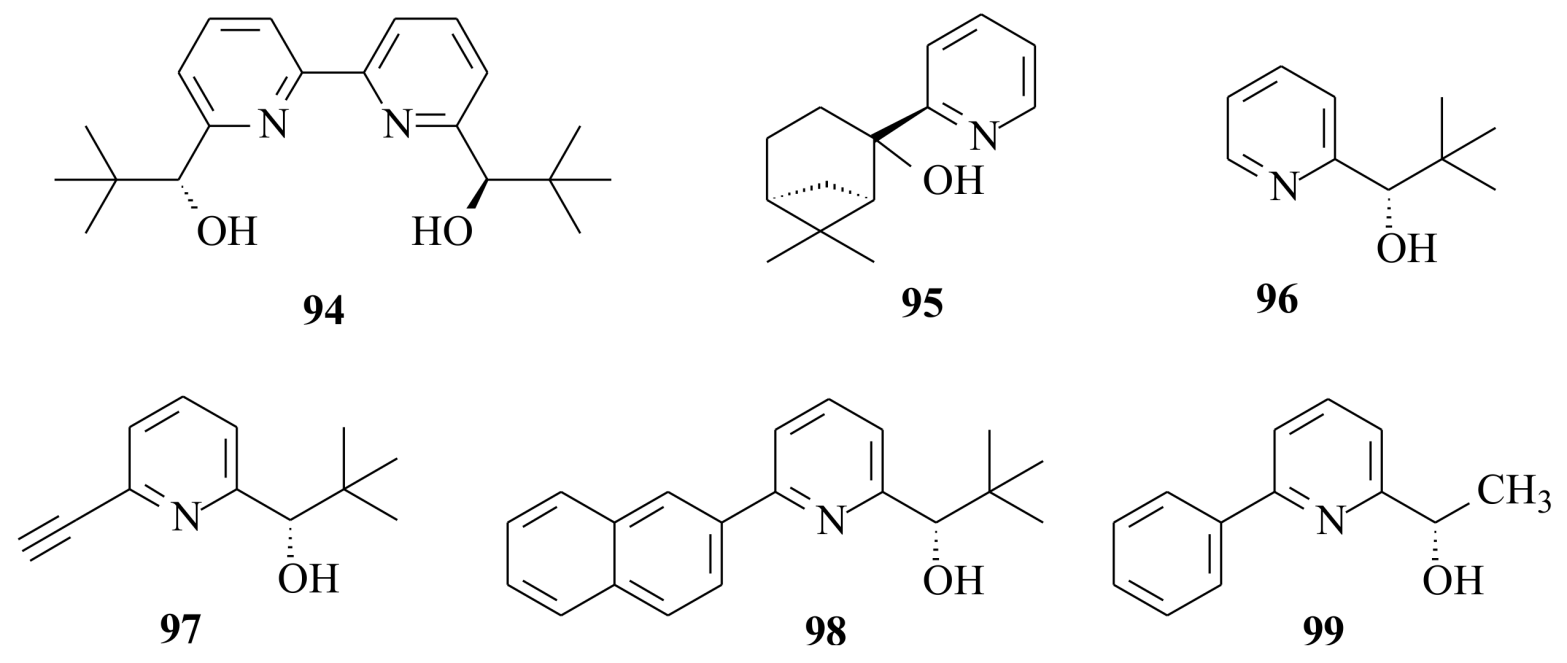
Pyridinyl alcohol compounds used as ligands in enantioselective additions.

Major methods of enantioselective synthesis of optically-active secondary alcohols are; (i) enantioselective alkylation of aldehydes and (ii) enantioselective reduction of ketones [5]. Optically-active secondary alcohols are components of many naturally occurring compounds, biologically active compounds, and materials such as liquid crystals [5]. A number of investigations have been made with the aim of enhancing the nucleophilicity of dialkylzinc (ligands shown in Figure 18).

The bipyridinyl diol **94** is an efficient enantioselective catalyst for the addition of Et_2_Zn to aldehydes (*p*-methoxyphenyl, *p*-chlorophenyl, *n*-hexyl, and benzaldehyde) in the presence of cobalt. Five mol% of the bipyridinyl diol **94** resulted in good c.y.s (65–96%) with high ee (up to 97%) [5,80].

The X-ray structure analysis of the Co-complex formed from the ester of the bipyridinyl diol and CoCl_2_·6H_2_O confirmed the C2-symmetry of the metal complex; the Co is surrounded tetrahedrally by the two nitrogen atoms of the bipyridinyl diol ligand and the two chlorine atoms. No coordinative bonding of the ether oxygens with the Co is observed.

Chelucci and Soccoloni [81] synthesized chiral pyridinyl alcohols **57a**,**b**, **95** and **96** for the catalytic addition of diethylzinc to benzaldehyde, 3-phenylpropanal, and 3-phenylpropynal. They used ligand **96** in order to define whether the enantioselective ability of 2-pyridinyl alcohols could be affected by the presence of another substituent on position 6 of the pyridine ring. The enantioselective addition of ZnEt_2_ to the aldehydes was done in the presence of catalytic amount (3 mol%) of **57a**,**b**, **95** and **96** in hexane/ether at 20 °C and resulted in very high conversion, but moderate to low enantioselectivity (see Table 5). The lower ee% of **96** in 3-phenylpropynal compared to benzaldehyde and 3-phenylpropanal once again indicated that a predictable improvement of the stereo-differentiating ability of 2-pyridinyl alcohols could be obtained through the introduction of a suitable substituent on the 6-position of the pyridine ring.

Bolm and co-workers [75,82] used the pyridinyl alcohols **48** [70], **79**, **84**, **85a**–**c**, **89**, **90**, **96**, and **97** in the enantioselective catalytic addition of diethylzinc to both aromatic and aliphatic aldehydes. All pyridinyl alcohols were used as chiral catalysts and the results obtained are presented in Table 6 and Table 7. All products in Table 6 are the (*R*)-enantiomers. For the reduction of benzaldehyde with the pyridinyl alcohols as chiral catalysts the following results were obtained (see Table 7).

Pyridinyl alcohols **84** and **98** with aryl substituents in 6-position were found to be the most active catalysts. Slow ethylation was observed for pyridines with non-aromatic substituents in 6-position. Catalysts **48** & **89** that bear chelating substituents at 6-position gave good yield with slightly lower ee. The importance of sterically bulky substituent at the chiral centre was shown by the weak enantioselectivity with catalysts derived from **90** and **99** that bear methyl instead of *tert*-butyl substituent.

**Figure 19:**
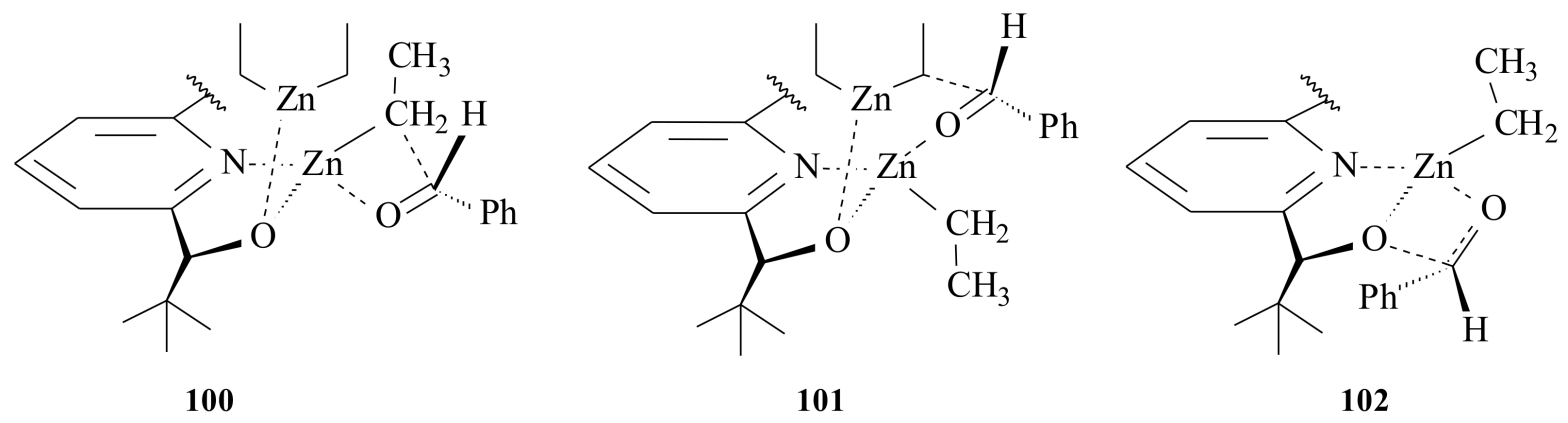
Intermediates proposed for the Zn-catalyzed addition reactions.

A possible mechanistic explanation was given by Bolm et al. [82] The three possible intermediates that might be present in solution are **100**–**102** (Figure 19). Ethyl transfer might have occurred via a 5/4-bridged intermediate **100** or as a result of six-membered cyclic transition **101** or else through the acetal-like intermediate **102**. In all cases, the aldehyde is activated by the formerly coordinated unsaturated zinc alkoxide. The stereochemical outcome of the alkylation is determined by the steric interactions between the aldehyde substituents. The catalyst will be liberated forming stable tetramers of the product zinc alkoxide. The mechanistic approach by Bolm et al. [82] gave no consideration to the C-6 substituted groups that clearly showed differences in the ee of the product as shown in Table 7. It is therefore very crucial to further investigate how the C-6-substituted bulky and chelating groups affect the enantioselectivity of the product.

Addition of diethylzinc to benzaldehyde by using bipyridinyl diols (*R,R*)-**48** and (*S,S*)-**88** as catalysts was also performed by Ishizaki et al. [83], with the intention to see the effect of the chiral alcohols in 6-position of the bipyridinyl diols. They used 5 mol% of **48** in hexane solvent at room temperature with a reaction time of 16 h and obtained (*R*)-1-phenyl-1-propanol (70% c.y. and 68% ee) and the same product with 77% c.y. and 65% ee by using **88** (5 mol%). They also used (*S*)-**96** in 10 mol% and four equivalents of ZnEt_2_ to obtain (*S*)-1-phenyl-1-propanol 70% c.y. and 66% ee. Although this was not the case with Bolm and co-workers’ [73,74,82] results, it is possible to conclude that the chiral alcohol in 6-position of bipyridinyl diol did not play an important role in this reaction.

**Figure 20:**
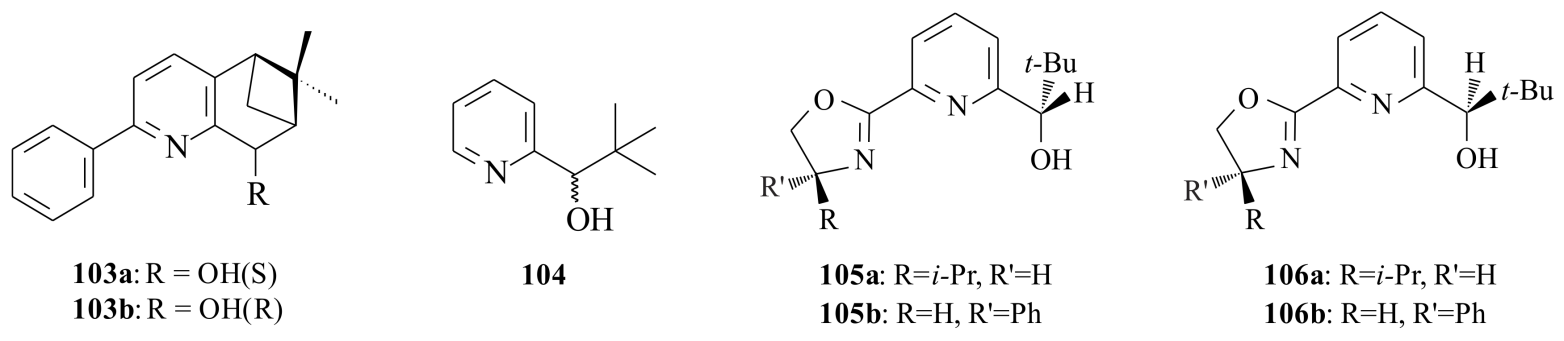
Substituted pyridinyl complexes used as ligands for addition reactions.

After synthesising substituted 2-phenyl-5,6,7,8-tetrahydro-6,6-dimethyl-5,7-methanoquinolines **103a,b,** Collomb and Von Zelewsky [84] tested the catalytic activities of the alcohols in the addition reactions of Et_2_Zn to benzaldehyde (see Figure 20). **103a** resulted in 75% c.y. and 28% ee, while **103b** resulted in 78% c.y. and 91% ee of the tertiary alcohol. The difference in the isomeric alcohols (*R*,*R*)-**48** and (*S*,*S*)-**88** did not show a significant change in the ee; however, the change in the pyridinyl alcohol isomers in **103a** and **103b** has shown an unexpected difference in the enantioselectivity of the product. This leads us to further investigate the effect of the structures of the groups in the chiral alcohol.

Mecedo and Moberg [15] received similar results to Bolm and co-workers [73,74,82] with respect to the amplification of the enantioselectivity by the chiral alcohol possessing a substituent in 6-position of the pyridine alcohol. They used the pyridinyl alcoholato ligands **104**, **105a**,**b** and **106a**,**b** (Figure 20), which they synthesized for this purpose, in the asymmetric addition of diethylzinc to benzaldehyde and *p*-chlorobenzaldehyde. The pyridinyl alcohol **104**, which does not possess a substituent in 6-position of the alcohol, resulted in low chemical and ee yield. The results are presented in Table 8.

The influence of the group in the 6-position of the pyridinyl alcohol is seen clearly as all of the 6-substituted pyridinyl alcohols resulted in better c.y.’s and ee’s than those without substituents. Looking once again into the differences only in the isomers of the alcohols **105a** versus **106a** and **105b** versus **106b**, it is clearly seen that **105a** and **105b** resulted in higher c.y.’s and ee’s. Therefore, this needs to be addressed to come up with a complete explanation as to why there existed a significant difference between isomeric alcohols.

**Figure 21:**
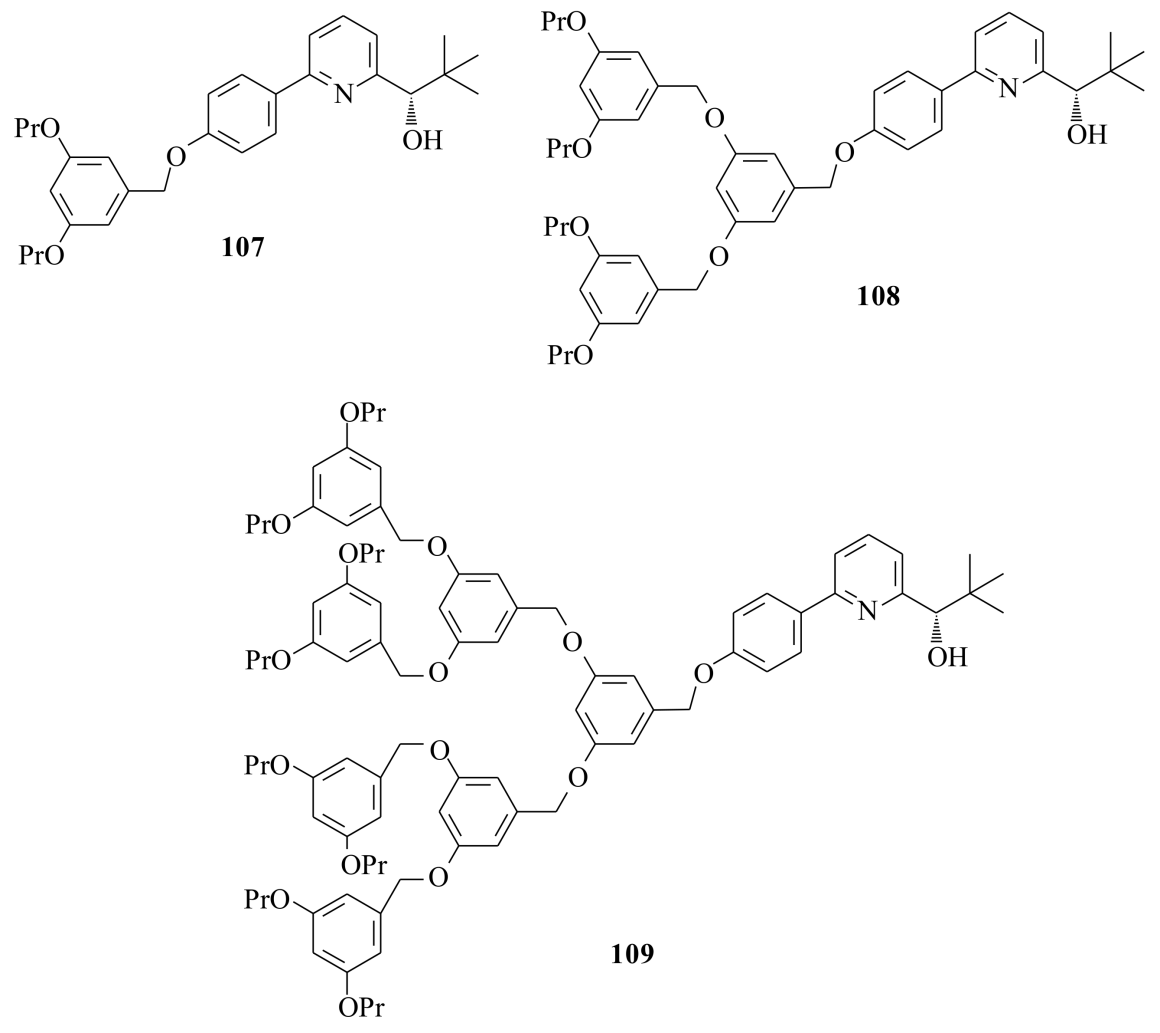
Dendrimeric pyridinyl alcohol compounds.

With the aim of determining the influence of the catalyst periphery on reactivity and enantioselectivity, the dendrimeric hyperbranched chiral catalysts **107** (90%), **108** (74%), and **109** (84%) (Figure 21) were synthesized by using Hawker and Frechet’s [85] convergent approach via the subsequent standard K_2_CO_3_/18-crown-6 mediated ether formations and alcohol to bromide conversions using CBr_4_/PPh_3_ [76].

Compared to the parent alcohol (*S*)-**84**, the enantiocontrol of the dendrimeric hyperbranched homogeneous chiral catalyst (HCC) was slightly (2–3% ee) lower. The asymmetric amplification phenomena showed that the molecular enlargement (at the level of **107**) does not seem to have a significant influence on the aggregation behaviour of the corresponding zinc alkoxide. This result does not agree with Bolm et al. [73,74,82] and Macedo and Moberg’s [15] result at all. Therefore, the need to further investigate the type, size and electronic character of the pyridinyl alcohols substituted at 6-position is crucial in order to come up with a synonymous conclusion. More emphasis should be placed on the mechanism of the reaction.

**Figure 22:**
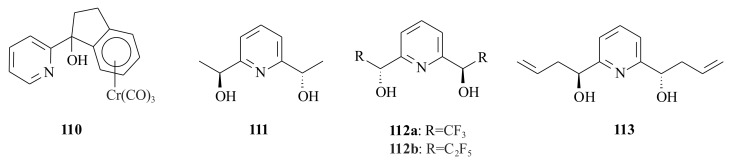
Cr complex and pyridinyl diols used in addition reactions of aldehydes.

Excellent c.y. (99%) with low enantiomeric purity (41%) product was obtained by Genov et al. [86] upon chiral addition of diethylzinc to benzaldehyde, using the pyridinyl alcohol **57b** (in toluene/hexane, 38 h, r.t.). They also used the structurally-related chiral pyridinyl alcoholato ligand **58** that resulted in 0% optical purity and an excellent c.y. (99%) with the same reaction conditions, but in 50 h. According to Genov et al. [86], the reason for the 0% ee was the insolubility of the zincalkoxide complex. The result obtained using pyridinyl alcohol **57b** is similar to that obtained by Chelucci and Soccoloni [81]. Enantioselective addition of diethylzinc to benzaldehyde in the presence of **110** (see Figure 22) resulted in 92% c.y. and 47% ee of the (*R*)-isomer of benzyl alcohol in 90 h [87]. The uncomplexed (amino) alcohol resulted in 89% c.y. with only 9% ee of (*R*)-alcohol in the same reaction condition. The presence of a tricarbonyl chromium unit, coordinated to the phenyl ring of the aryl pyridinyl alcohol catalyst, induced a significant increase in the enantioselectivity of the catalysed addition of diethylzinc to benzaldehyde. The pyridine diols **48**, **111**, **112a,b** and **113** (Figure 22) yielded very good chemical yields and showed good enantioselectivities in the nucleophilic addition of diethylzinc to aliphatic and aromatic aldehydes [88].

Comparable ee’s were obtained for the catalysis in **48** (70% c.y. and 68% ee), **112b** ((75% c.y. and 63% ee) and **112a** (89% c.y. and 56% ee) during addition to benzaldehyde. The same chemical (87%) and ee (68%) yields were obtained during addition of diethylzinc to *n*-pentanal and cyclohexanal. All of them yielded the (*R*)-enantiomer, except **111** and **113**, which of course yielded the (*S*)-isomer plus the least in ee (32% and 38%, respectively). In this specific case, it is not very clear as to why the relatively small groups of CF_3_ (in **112a**) and C_2_F_5_ (in **112b**) in the chiral position of the pyridinyl alcohols did not decrease the yield and ee of the product, as it is in the pyridinyl alcohols **111** and **113**. Comparing the ee obtained by using pyridinyl alcohol **111** with **112a**, we can say that electron-withdrawing electronic effect can somehow increase the ee of the product. The electron-withdrawing effect can also be seen comparing the ee’s of the highly electron-withdrawing substituent C_2_F_5_ in **112b** (63%) with that of the less powerful electron-withdrawing substituent CF_3_ in **112a** (56%).

**Figure 23:**
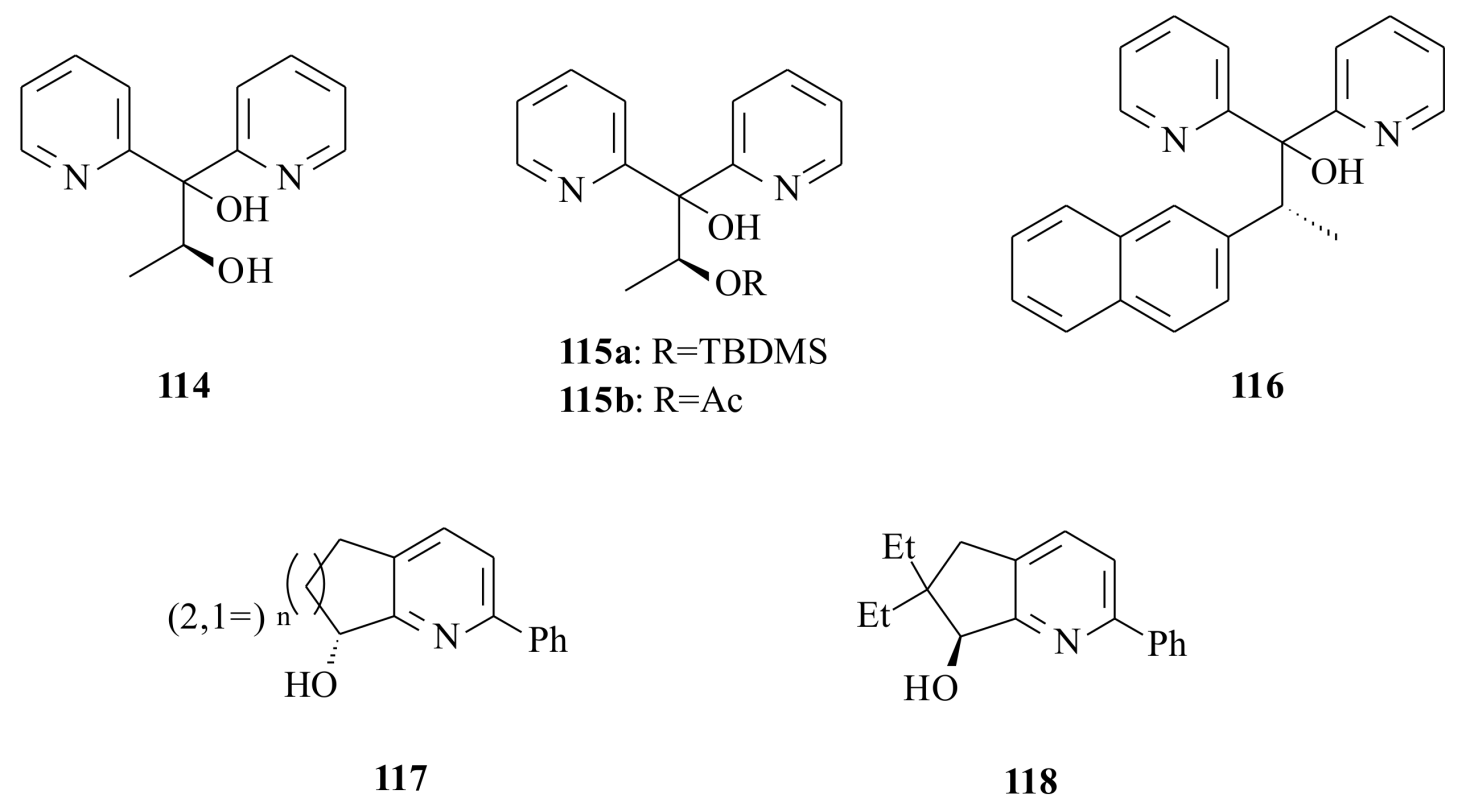
Pyridinyl alcohols used as ligands in the addition of diethylzinc to benzaldehyde.

The chiral oligopyridine derivative ligands **114**, **115a**,**b** and **116** (Figure 23) were prepared by Kotsuki et al. [89], with the aim of catalysing the nucleophilic addition of diethylzinc to benzaldehyde. The highest yield was obtained by the chiral catalyst **115a** (90% c.y. and 70% ee) in 10 h. Addition of the additive Ti(O*t*-Bu)_4_ decreased the yield to 84% c.y. and 51% ee. On the other hand, the chiral catalyst **114** has shown increment both in the chemical as well as ee yield upon addition of the additive Ti(O*t*-Bu)_4_ to the reaction mixture from 64 to 88% c.y. and 2 to 12% ee. The pyridinyl alcohol ligand **115b** yielded only 10% c.y. and 3% ee in 48 h. Ligand **116** yielded 100% c.y., but only 6% ee. All of them yielded the (*S*)-configuration except **114**, which resulted in the (*R*)-isomer upon using the additive Ti(O*t*-Bu)_4_.

**Scheme 10:**
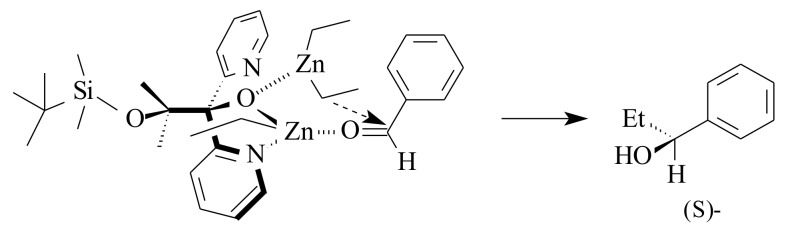
The mechanism of benzaldehyde alkylation into the (*S*)-alcohol by Et_2_Zn.

A plausible mechanistic explanation of alkylation by Kotsuki et al. [89] using **115a** is similar to Bolm et al.’s [82] intermediate **101** (see Scheme 10).

The pyridinyl alcoholate ligands (*S*)-**117** and (*R*)-**118** (Figure 23) were applied to the enantioselective addition of diethylzinc to aromatic aldehydes resulting in good yields (82% and 80%, respectively) and moderate enantioselectivities (90% and 87%, respectively) in the case of benzaldehyde [90]. The chemical yield and enantioselectivity decreased for *p*-chlorobenzaldehyde (62%, 49% and 54%, 48%, respectively), *p*-methoxybenzaldehyde (51%, 71% and 47%, 50%, respectively) and (*E*)-*β*-phenylpropenal (76%, 73% and 9%, 2%, respectively). The ee’s obtained by using (*S*)-**117** and (*R*)-**118** are very close to the ee obtained by the structural analog Collomb and Von Zelewsky’s [84] pyridinyl alcohol **103b**.

**Figure 24:**
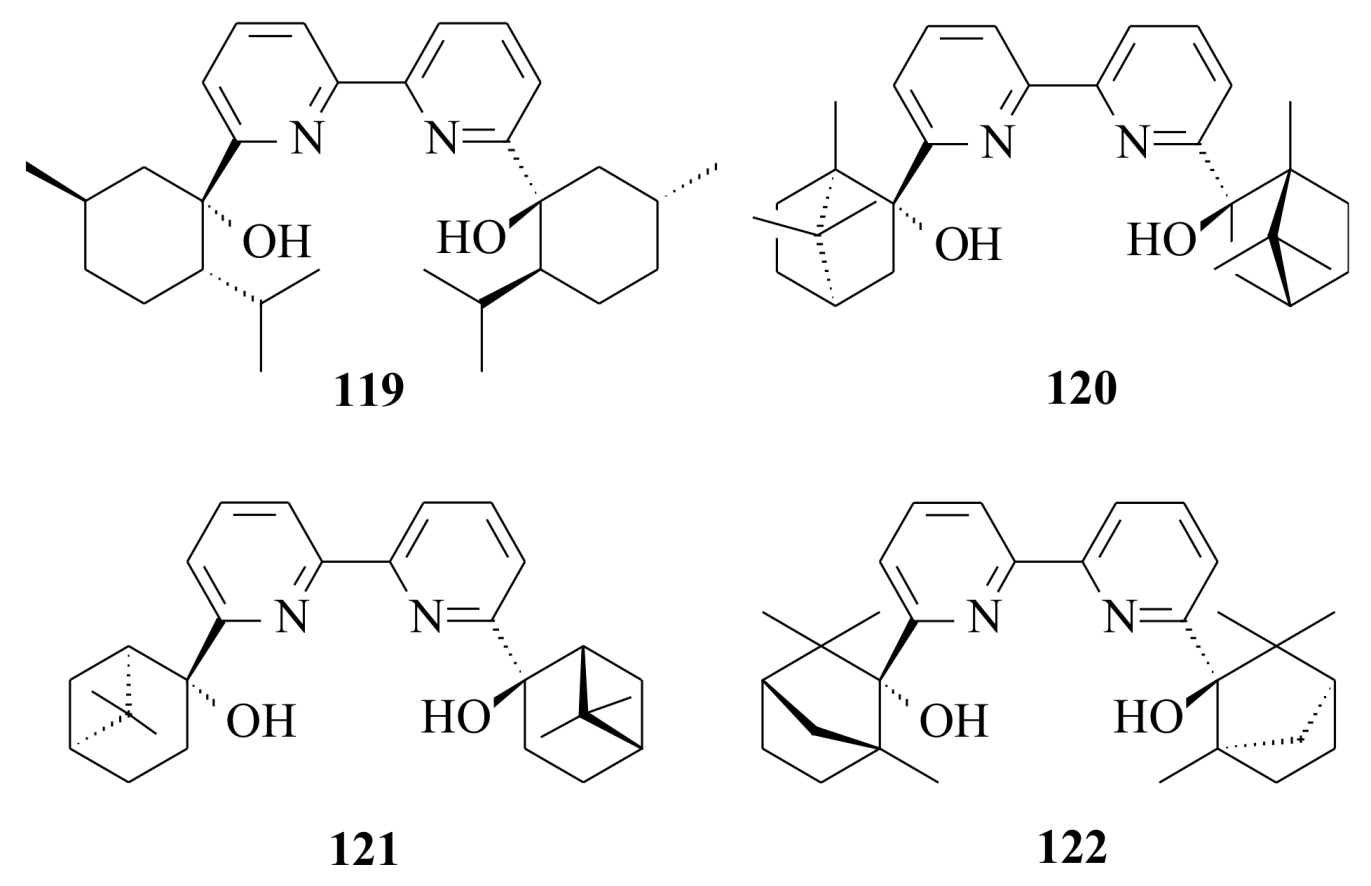
*C_2_*-symmetric chiral bipyridinyl diol ligands.

Enantioselective addition of diethylzinc to benzaldehyde catalysed by *C_2_*-symmetric chiral bipyridinyl diol ligands **119**–**122** (Figure 24) resulted in 30 to 86% c.y. with 23 to 95% ee [91]. The enantioselectivity depended on the type of solvent used. For example, in CH_3_CN, the alcoholate ligand **120** resulted in the least c.y. (30%) and ee (23%), whereas in toluene a very good c.y. (86%) and moderate ee (95%) were obtained. The absolute configuration of the product in all ligands except in **119** was ‘*R*’. Because the alcoholate ligand **120** resulted in a better yield, they used it to catalyse the diethylzinc addition to other aromatic and aliphatic aldehydes, which resulted in good c.y. (52–74%) and low to moderate ee’s (20–80%). The better yield was observed for cyclohexanal (66% c.y. and 80% ee).

**Figure 25:**
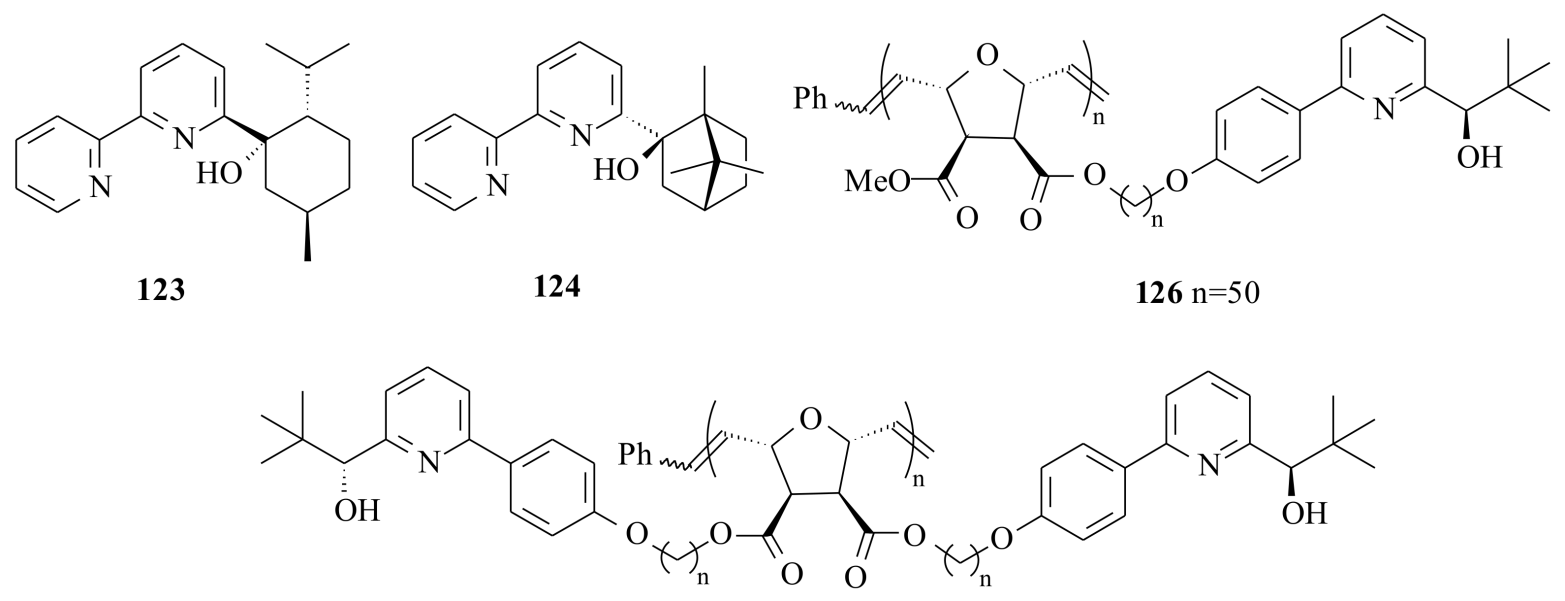
Bipyridyl and polymer-supported pyridyl alcohol ligand precursors.

With the intention to see whether *C_2_*-symmetry of the bipyridinyl diol ligand is helpful in achieving high enantioselectivity in the alkylation of aldehydes, Kwong and Lee [91] synthesized the 6-phenyl and 6-(2-pyridinyl) substituted pyridinyl alcohols **79** and **80**. All the newly-synthesized ligands were active in catalysing the ethylation of benzaldehyde by diethylzinc. Using the alcoholate ligands **79** and **80,** the ee’s obtained were 76% (*S*) and 78% (*R*), respectively, with complete conversion of benzaldehyde in 1.5 h (for both ligands). Due to the high concentration of diethylzinc in the reaction mixture, the remaining *N,O*-part of the ligand coordinated to other zinc atoms during reaction and the two *N,O* units probably function independently. On the other hand, further ethylation with the 6-(2-pyridinyl) substituted **123** and **124** (see Figure 25) gave 21% (*R*) and 65% (*R*) with complete conversion of benzaldehyde in one and three hours, respectively. Similar results were obtained previously with other pyridinyl alcohols and bipyridinyl diols [92]. Comparison of the activity and enantioselectivity of ligands **79**, **119**, **123** and **80**, **120**, **124** indicated that bipyridinyls **119**–**122** function as *N,O*-ligands in diethylzinc addition reactions.

Industrial processes with polymer-supported catalysts are common due to the durability and ease of separation of catalysts. One strategy to prepare these catalysts is the attachment of a previously identified catalytically-active compound to a preformed polymeric support. An alternative approach for the generation of polymeric multivalent chiral catalysts **125** and **126** (Figure 25), which is based on ROMP of strained bicyclic olefins, was reported by Bolm et al. [93] In both **125** and **126**, a chiral hydroxylpyridinyl fragment was the catalytically active subunit.

Enantioselective addition of diethylzinc to benzaldehyde in the presence of **125** and **126** as catalysts resulted in 77 to 88% c.y. with 71 to 73% ee benzyl alcohol. A slight decrease in catalytic activity was observed compared to low molecular weight pyridinyl alcohols (starting material and intermediate) in a similar way to Bolm et al.’s [79] dendritic hyper branched homogeneous chiral catalysts **107**–**109**.

**Figure 26:**
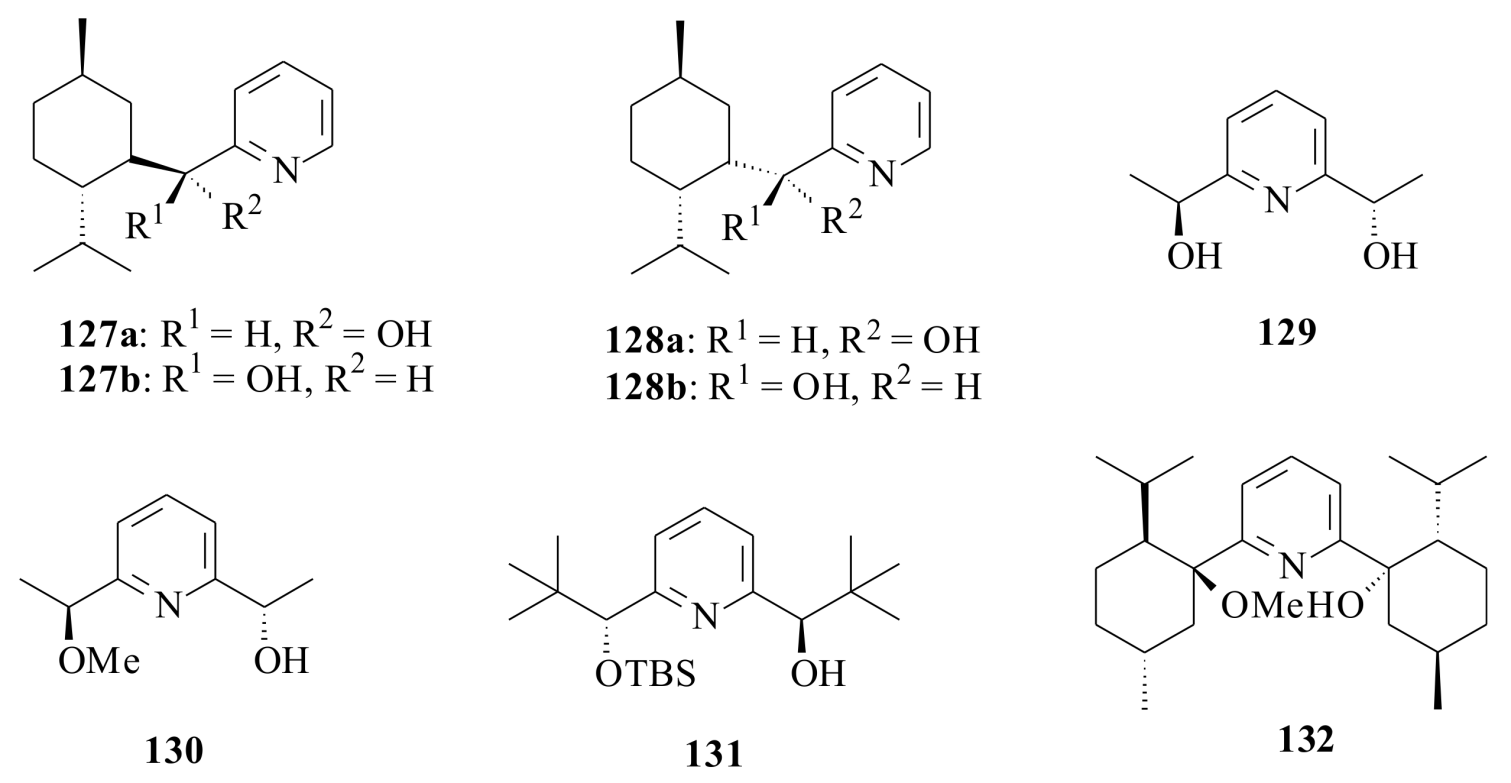
Chiral pyridinyl alcohols (*α*), diols (*α,α*’) and alkoxy-hydroxyls (*α,α*’) used as ligands.

The pyridinyl alcohols (**127a**,**b** and **128a**,**b**) (Figure 26) of Cunningham et al. [94] were tested for their catalytic activity in the nucleophilic addition of diethylzinc to benzaldehyde and resulted in very good chemical yields (80–96%) and poor enantioselectivities (8.5–22.5%). All of them resulted in the (*R*)-configuration of the benzyl alcohol. The highest ee (22.5%) was observed during the catalysis of **128a** and the least (8.5%) in **128b**. Once again, orientation of the bulky group in the pyridinyl alcohol was observed affecting the ee of the product.

The *R*-pyridinyl alcohol resulted in high ee% of the product in a similar way to that of Macedo and Moberg’s [15] pyridinyl alcohols **105a**,**b** (the *R*-isomers) compared to **106a**,**b** (the *S*-isomers) and to Collomb and Von Zelewsky’s [84] isomeric alcohols **103b** (*R*-isomer) and **103a** (*S*-isomer).

The catalytic activities of the tridentate pyridinyl alcohols **88**, **119** and **129** were compared to the bidentate pyridinyl alcohols **57a** and **130**–**132** (Figure 26) by Goanvic et al. [95] in the enantioselective alkylation of benzaldehyde with diethylzinc to see the effect on the second arm of pyridine diols. The yields of the benzyl alcohol follows **88** (77% c.y. and 65% ee), **119** (81% c.y. and 55% ee) and **129** (84% c.y. and 4% ee) for the tridentate alcohols and **57a** (93% c.y. and 21% ee), **130** (83% c.y. and 47% ee), **131** (79% c.y. and 67% ee (*R*)) and **132** (81% c.y. and 35% ee) for the bidentate alcohols. All of them, except **131**, resulted in the (*S*)-enantiomer of the alcohols. The results show that only one half of the *C_2_*-symmetric ligand is part of the actual complex. The second arm seems to only play a screening role helping in the differentiation of the two enantiotopic faces of the aldehyde within the catalytic complex. Therefore, it is worthwhile to conclude that the beneficial role of a tridentate catalyst compared to bidentate ligand is not as important as expected. 

**Figure 27:**
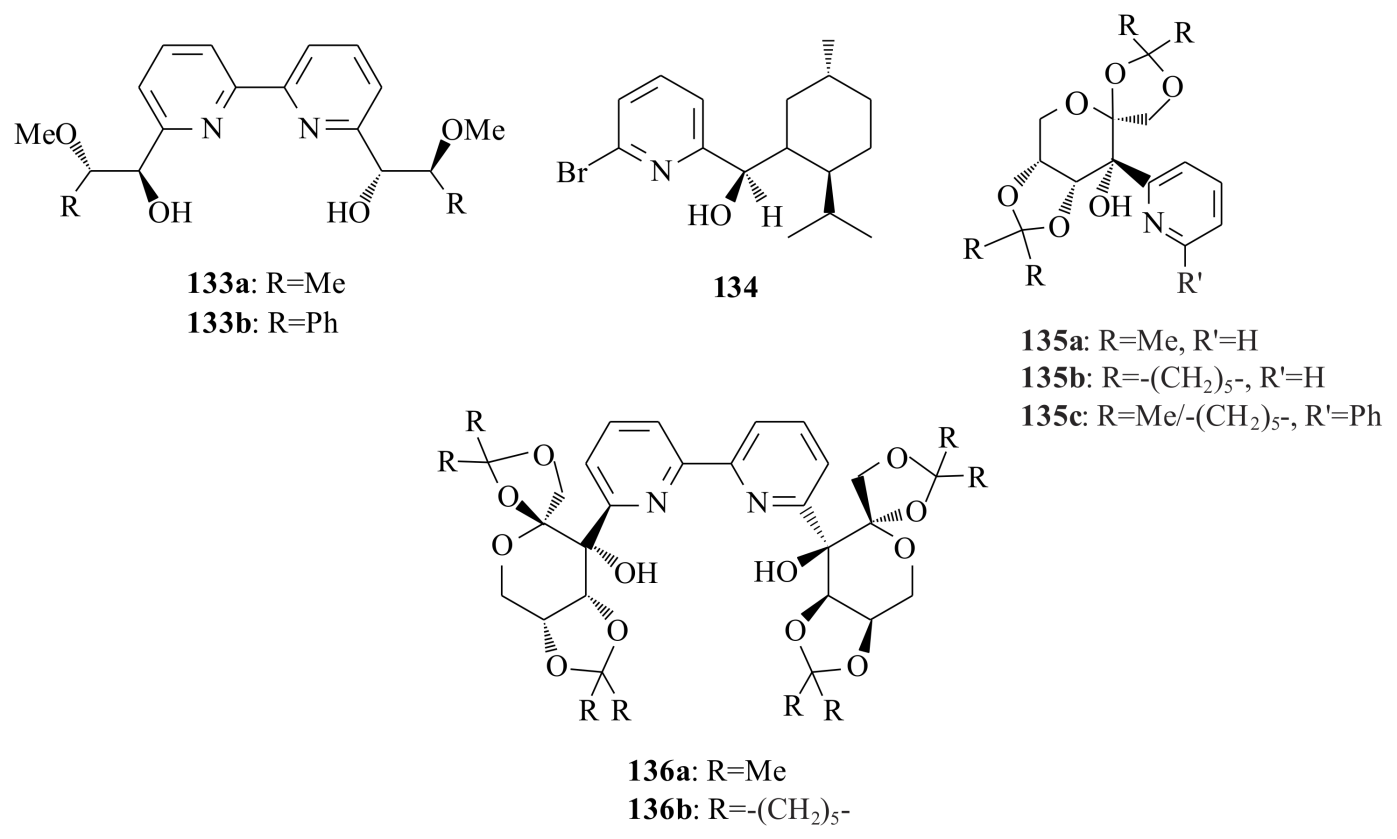
Complex pyridinyl alcohols used in the diethylzinc addition reaction.

**Scheme 11:**
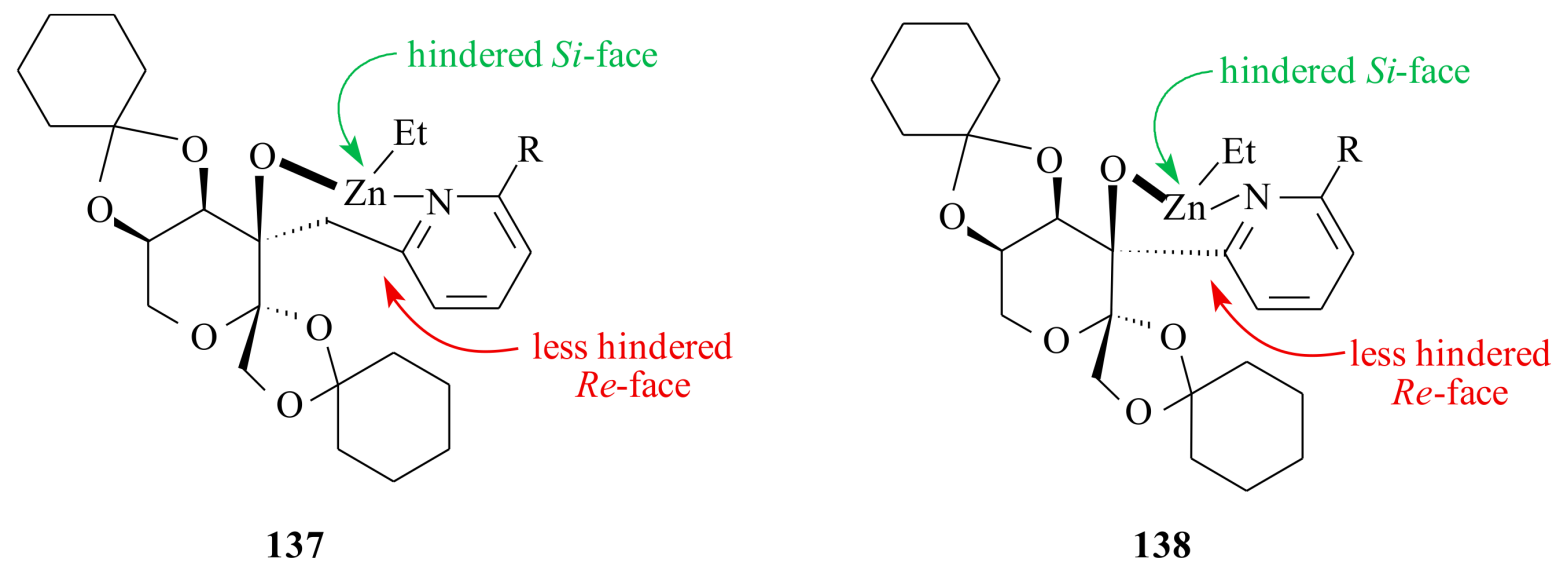
Mechanism of Et_2_Zn addition to benzaldehyde using pyridinyl alcohols **137** and **138**.

The bipyridinyl diols **133a**,**b** and the pyridinyl alcohol **134** were tested for their catalytic activities in the enantioselective diethylzinc addition to benzaldehyde by Rahm et al. [96,97], following their synthesis (Figure 27). The bipyridinyl diols **133a**,**b** resulted in 47% c.y. with 96% ee and 74% c.y. with 83% ee, respectively, of the (*S*)-isomer, whereas **134** yielded the (*R*)-isomer in 94% c.y. with 28% ee. Very similar concluding results (to that of Rahm et al. [96,97]) were obtained by Huang et al. [98] upon using the bidentate pyridinyl alcohols **135a**–**c** and the tridentate pyridine diols **136a**,**b** in the enantioselective addition of diethylzinc to benzaldehyde in toluene at 0 °C (Figure 27). The enantioselectivities of the bidentate pyridinyl alcohols go up to 25%, while that of the tridentate alcohols is only 15%. On the other hand, they attempted to see the structural influence of the pyridinyl alcohol on the catalytic addition reaction upon synthesising pyridinyl alcohols having one methylene group longer backbone chain between the coordinating *N* and *O* atoms. The results in the catalytic addition of diethylzinc to benzaldehyde revealed that the pyridinyl alcohols with longer backbone chain resulted in much higher ee (75–83%) compared to the corresponding pyridinyl alcohols **135a**–**c** (7–25% ee). The possible explanation for the differences in the catalytic activities and enantioselectivities could be made by considering the conformational analysis of the ethylzinc (amino)alkoxide complexes of the pyridinyl alcohols. Ligands with one more CH_2_ group longer backbone possibly form the six-membered ring ethylzinc (amino)alkoxide **137**, while those with short backbone chain form the complex **138** in situ. It is easier for the benzaldehyde to be coordinated to the *Re*-face of **137** than that of the *Re*-face of **138** (see Scheme 11). Moreover, the difference between the two enantiofaces in **138** is less than that of **137**; therefore, both the activity and enantioselectivity of catalysts **138** should be lower.

**Figure 28:**
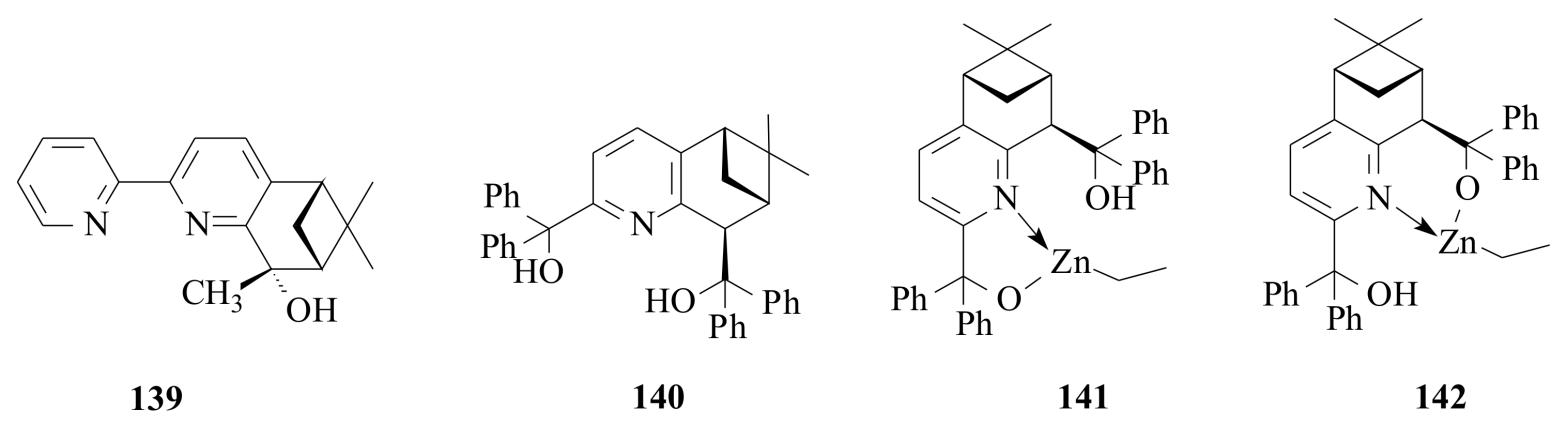
Sterically strained pyridinyl alcohols used in the diethylzinc addition reaction.

Pyridinyl alcohol **139**-catalysed asymmetric alkylation of substituted benzaldehyde using diethylzinc resulted in good chemical yields (31–85%) and moderate enantioselectivities (56–78%) [99] (Figure 28). The *ortho*- and *para*-substituted electron-releasing substituents yielded high enantioselectivities (see Table 9).

This may happen due to the increased π-π interaction between the pyridinyl group of ligand **139** and the aromatic ring of the benzaldehyde. This, in turn, will fix tightly the aromatic ring of benzaldehyde such that the *si*-face of the carbonyl group is preferentially alkylated increasing the enantioselectivity. The reaction was done by using catalytic amount (5 mol%) of the ligand **139** in hexane at room temperature for 5 h. The reaction condition is optimised. Ligand **139**, structurally closely related to the Collomb and Von Zelewsky’s [84] pyridinyl alcohol **103a**, resulted in quite different ee’s of the benzyl alcohols.

The presence of the methyl group in **139** might be the reason for the high ee. However, a further study of the same substituents on the benzaldehyde to that used in **139** and performing the diethylzinc addition reaction using **103a** would clarify the effect of both the pyridine and the methyl substituents in **103a** and **139**, respectively. The pyridine diol **140** was used in the enantioselective addition of diethylzinc to benzaldehyde, but resulted in only 8% ee of the 1-phenyl-1-propanol [100].

Steric energy calculations using the MM2 program indicated that intermediate **141** has lower steric energy (49.322 kcal/mol) than intermediate **142** (54.283 kcal/mol) (Figure 28). This therefore tells us that the reaction centre might have shifted far way to the second alcohol that cannot induce high enantioselectivity, which is in agreement with Huang et al.’s [98] proposal.

Capracotta and Comins [101] tested the catalytic activities of the pyridinyl alcohol **143** in the diethylzinc addition to benzaldehyde; however, the yield obtained was very small (2% ee). They^[^ used 20 mol% of catalyst (**55**) in toluene at 0 °C for 24 h.

The c.y. was not good either (42%). This might be due to the bulky cycloamino substituent at the 3-position of the pyridine ring that possibly hinders the approach of the carbonyl group towards the pyridinyl alcohol-Zn-alkyl complex via the *si*-face easily.

**Figure 29:**
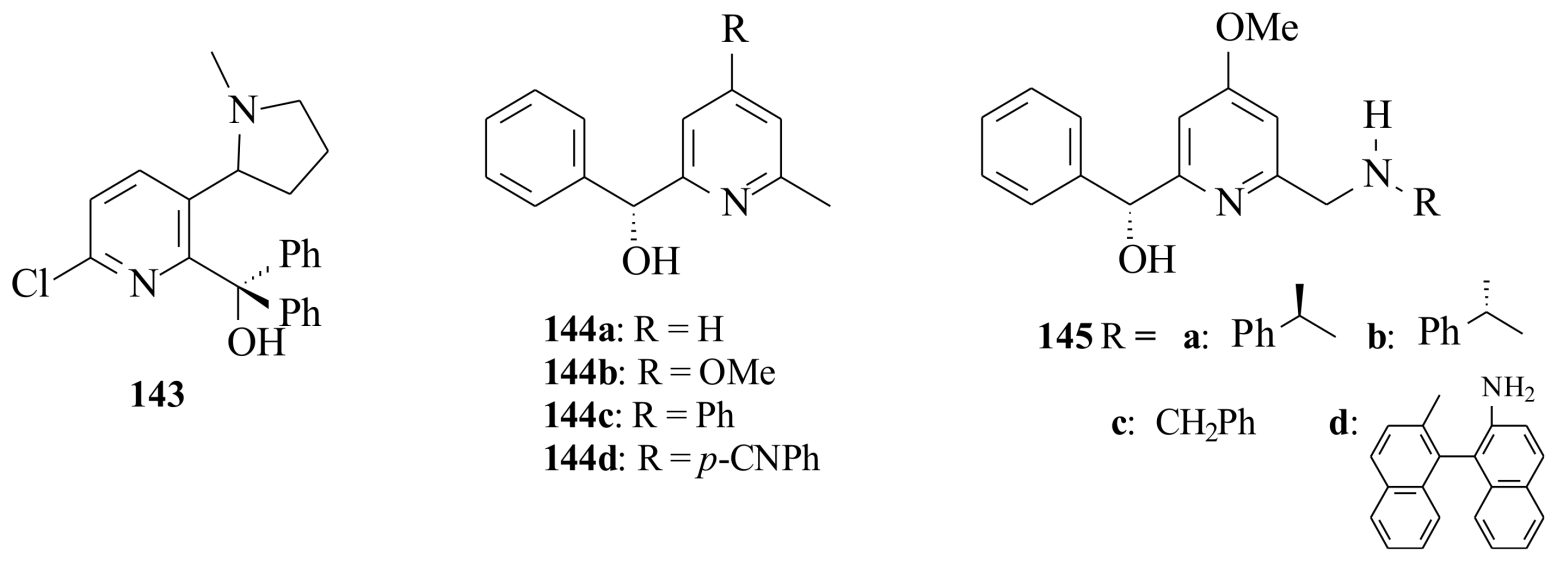
Pyridinyl alcohol compounds substituted on the pyridinyl group.

Eidamshaus and Reissing [102,103] synthesized pyridinyl alcohols **144a**–**d** and **145a**–**d** (Figure 29) via reductive amination of the corresponding pyridinyl aldehyde. The pyridinyl alcohols **144a**–**d** were used (12 mol%) in the enantioselective addition of diethylzinc to benzaldehyde and gave very good yields (68–93%) with moderate enantioselectivity (69–74%) of the (*R*)-configuration product. The electronic properties of the pyridine ligands did not show a significant influence on the chiral induction. The aminomethyl-substituted pyridine derivatives **145a**–**d** resulted in a relatively high ee (58–88%) and low c.y.’s (48–85%). The highest chemical (87%) and ee (88%) were obtained by ligand **145a** and the least ee (58%) by ligand **145d**. The reactivity differences of **145a**, **145b** and **145c** cannot be explained by the formerly presented models. In order to explain this fact, Eidamshaus and Reissing [102,103] proposed the new transition states **146** and **147**. They followed the 5/6-membered ring postulate formed by the pyridine ring chelating an alkyl zinc moiety that acts as Lewis acid and coordinates the reacting aldehyde.

**Scheme 12:**
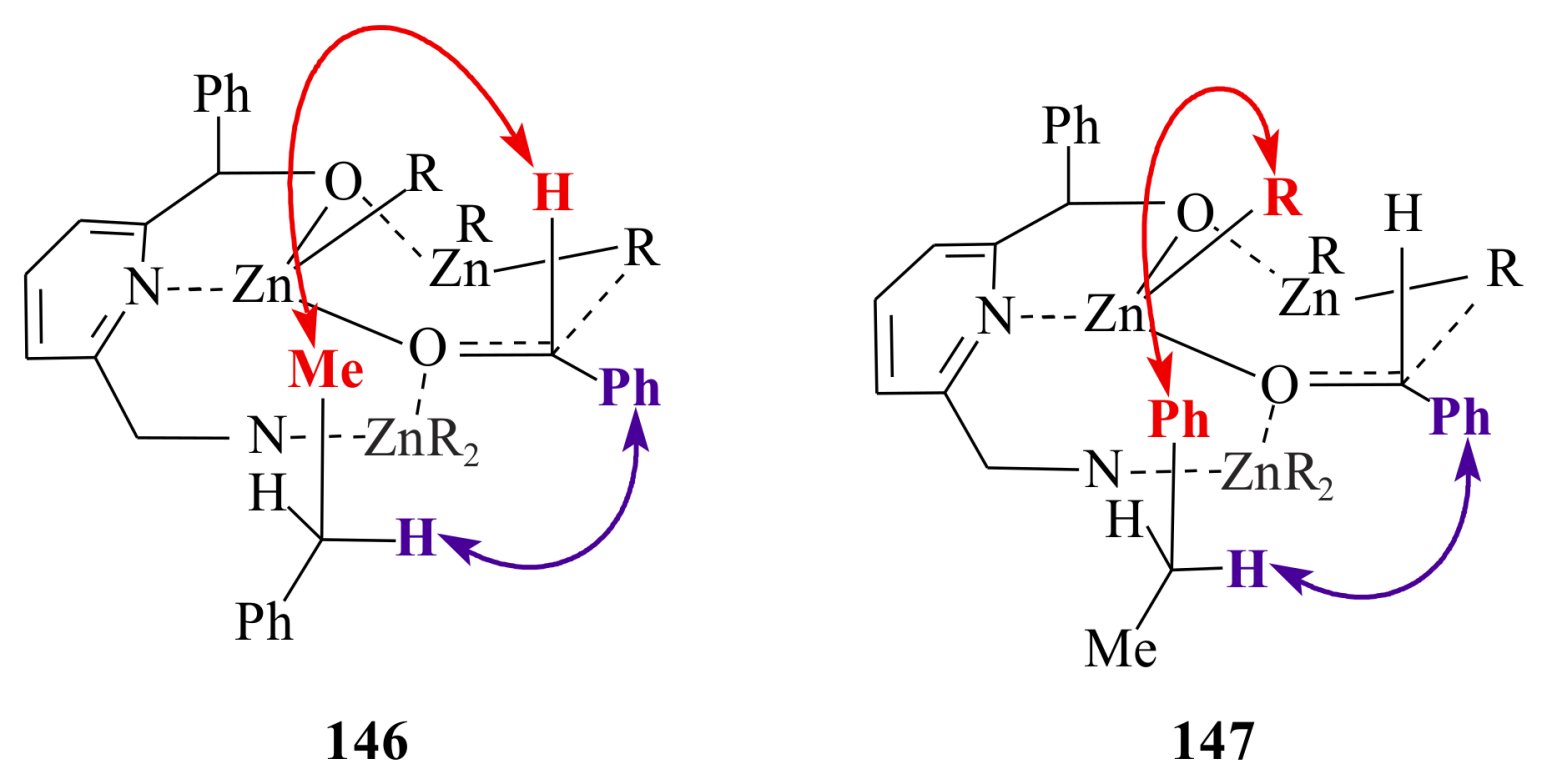
Pyridine ring chelation and *re*-face attack of benzaldehyde by Et_2_Zn.

This coordination explains the preferential *re*-face attack; however, it was not possible to explain the influence of the C-6 aminomethyl side chain of the pyridinyl alcohol ligands **145a**–**d** (see Scheme 12). It was suggested that a third molecule of diethylzinc is involved being coordinated between the amino group and the carbonyl oxygen so that the aminomethyl side chain should be close to the carbonyl centre and leads to the preferred transition state **146**. In the transition state **146**, the bulky phenyl group of the aminomethyl group points away, whereas the methyl group enhances the asymmetric induction as it points in the same direction as the C-2 phenyl group. In transition state **147**, that is the transition state when ligand **145b** is used, the phenyl group of the aminomethyl side chain probably points up generating a sterically more crowded transition state. A decreased activation of the carbonyl group and lower enantioselectivity is due to the large distance to the aminomethyl side chain.

### 2.7. Allylic Oxidation

Reactions at allylic positions are of very great synthetic importance for the organic chemist as they provide a chiral allylic ester that can easily undergo further functionalisation. Allylic oxidations are catalysed by copper(I)-complexes. The addition of pyridinyl alcohols as chiral auxiliary ligand enhanced the enantioselectivity of copper(I)-catalysts.

**Scheme 13:**
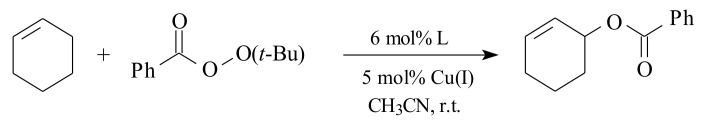
Copper(I)-catalysed allylic oxidation of cyclohexene.

Chiral copper(I)-bipyridinyl complexes were prepared and used as catalysts in the enantioselective allylic oxidation of cyclic alkenes using *tert*-butylperbenzoate [104]. The pyridinyl alcoholato ligands **79**–**81** and **119**–**124** were synthesized by Kwong and Lee [91]. All of the ligands were used in the allylic oxidation of cyclohexene as a preliminary work in 6 mol% ligand and 5 mol% catalyst in CH_3_CN at room temperature (see Scheme 13). The Cu(I)-catalysts used in the preliminary test reaction include: [Cu(CH_3_CN)_4_]PF_6_, [Cu(CH_3_ CN)_4_]ClO_4_, Cu(OTf), CuCl and CuBr. The ligands converted cyclohexene with 38 to 80% c.y. and 8 to 65% ee.

**Scheme 14:**
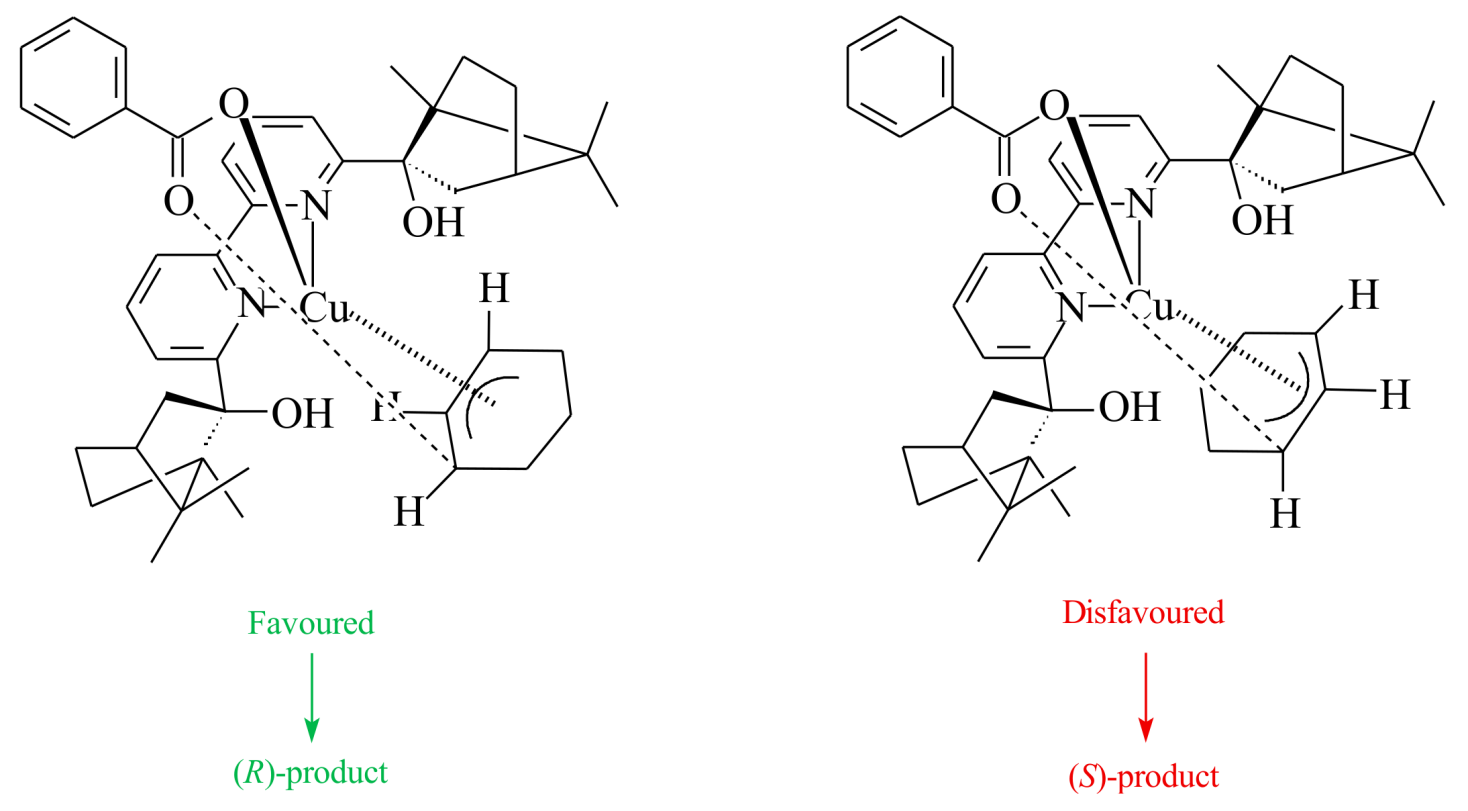
Model transition states for the copper complex of **120** in the allylic oxidation of cyclohexene.

However, ligands **123, 124** in [Cu(CH_3_CN)_4_]PF_6_ catalyst and ligand **120** in CuCl and CuBr catalysts yielded 0% ee. Ligands **119**–**122** resulted in the (*R*)-isomer, while ligands **81** and **124** in the (*S*)-isomer of the product. Ligands **120** and **124** were found to be the best ligands as they yielded 58% ee (63% c.y.) and 65% ee (43% c.y.) with conversions 95% and 65% in Cu(OTf) and [Cu(CH_3_CN)_4_]PF_6_ catalysts, respectively. These ligands were also used in the allylic oxidation of cyclopentene, cycloheptene and cyclooctene [91]. In cyclopentene, ligand **120** yielded 59% c.y. and 61% ee of the (*R*)-product with 81% conversion using CuBr catalyst, while **124** resulted in 83% c.y. and 56% ee of the (*S*)-product with 78% conversion using [Cu(CH_3_CN)_4_]PF_6_ catalyst. In the allylic oxidation of cycloheptene and cyclooctene, the copper salt [Cu(CH_3_CN)_4_]PF_6_ was used and the reaction stayed for four days at room temperature. Ligand **120** resulted in the highest ee’s, 61% and 70% of the (*R*)-product in cycloheptene and cyclooctene, respectively, while ligand **124** yielded 21% and 12% ee’s of the (*S*)-isomer product, respectively. The effect of the copper salt is dependent on the substrate and the specific ligand. Ligands **81** and **120**–**124** do not function as *N,O*-bidentate ligands in allylic oxidation reaction, but rather function as *N,N*-bidentate ligands [91]. In order to explain the results obtained, similar models to that of *N,N*-bidentate [105] and *N,N,N*-tridentate [106] were proposed using ligands **120** and **124** with cyclohexene (see Scheme 14).

**Scheme 15:**
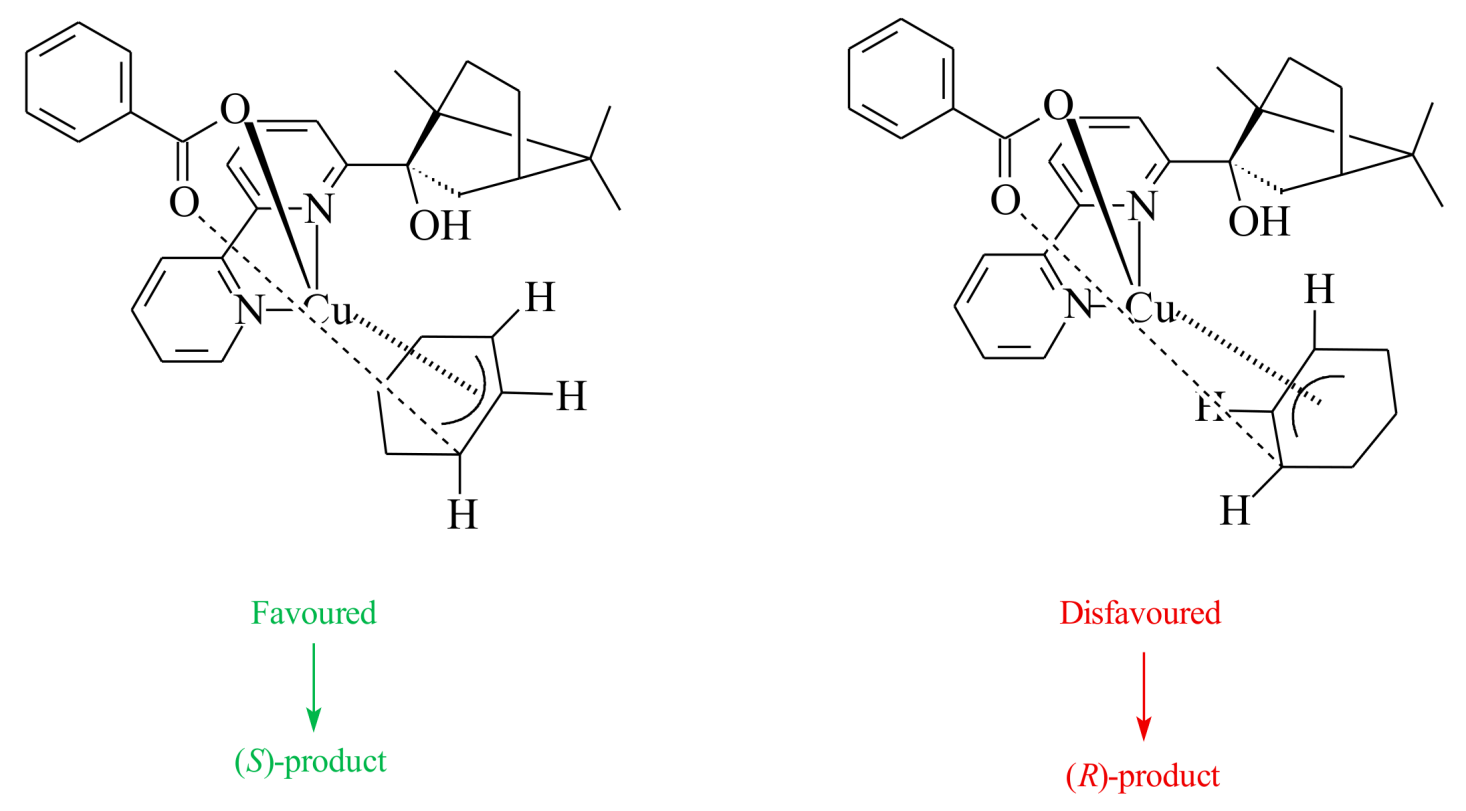
Model transition states for the copper complex of **124** in the allylic oxidation of cyclohexene.

In the model for **120** (Scheme 14), the favoured transition states are shown with the cyclohexenyl and benzoate groups positioned so as to minimise the interaction with the bulky pendant group of the pyridine ligand. The removal of the bulky pendant group from one side of the ligand in the transition state for the model of **120** leads to the change in the coordination of the cyclohexyl group. In order to further minimise interaction with the pendant group of the ligand, the opposite prochiral face then coordinates to the metal centre in the model for **124** (see Scheme 15).

Lee et al. [69] prepared ligands **57b**, **81** and **127a** and tested their catalytic application in the allylic oxidation of cyclohexene with *tert*-butylperoxy benzoate using Cu(OTf) and/or Cu(OTf)_2_ catalysts. The copper complexes [Cu(L)_2_] of the ligands were found to be inactive in copper-catalysed allylic oxidations of cyclohexene. The reaction of the complexes with triflic acid made them active even though no enantioselectivity was obtained for the isolated complexes. The in situ generated complexes using Cu(OTf) or Cu(OTf)_2,_ however, resulted in 56 to 80% c.y. and 11 to 38% ee, all (*R*)-isomers of product except for Cu(L) (where L = **127a**), which resulted in the (*S*)-isomer. Complexes synthesized from Cu(OTf) showed better rates and enantioselectivities than those from Cu(OTf)_2_. Ligand **81** was found to be the better yielding ligand resulting in 38% ee with 87% conversion after 81 h.

Even though promising results in both chemical yield and enantioselectivity are obtained, there are very few literature reports in this area. Compared to the very huge number and structurally diversified pyridinyl alcoholato ligands, very little work has been done. Therefore, this could be a potential area that needs further investigation in order to obtain a better chiral copper catalyst that catalyses allylic oxidation reactions resulting in a better yield and enantioselectivity.

### 2.8. Ring-Opening Reactions of Meso-Epoxides

Lewis acid catalysed reactions are of great interest because of their unique reactivities, selectivities and mild reaction conditions. A large number of Lewis acid catalysts that are used in the synthesis of natural and synthetic compounds have been dealt with in the past decades. The traditionally used Lewis acids, such as AlCl_3_, SnCl_4_, TiCl_4_, and BF_3,_ were needed in more than the stoichiometric amounts, are moisture sensitive, and cannot be recovered and reused after the reactions are completed [107]. The catalytic enantioselective ring-opening of *meso*-epoxides is an efficient means to convert readily available, inexpensive bulk chemicals into chiral, 1,2-difunctionalised fine chemicals in enantiomerically enriched form [108]. In the epoxide ring-opening reactions, the catalyst behaves as a Lewis acid, directing the incoming nucleophile into the enantiotopic faces of the epoxide in an S_N_2 fashion. To date, the epoxide ring-opening reactions that led to 1,2-azido alcohols, 1,2-halohydrines, 1,2-cyano alcohols, 1,2-diol monoesters and monoethers and 1,2-mercapto alcohols resulted in good to excellent enantioselectivities.

**Scheme 16:**
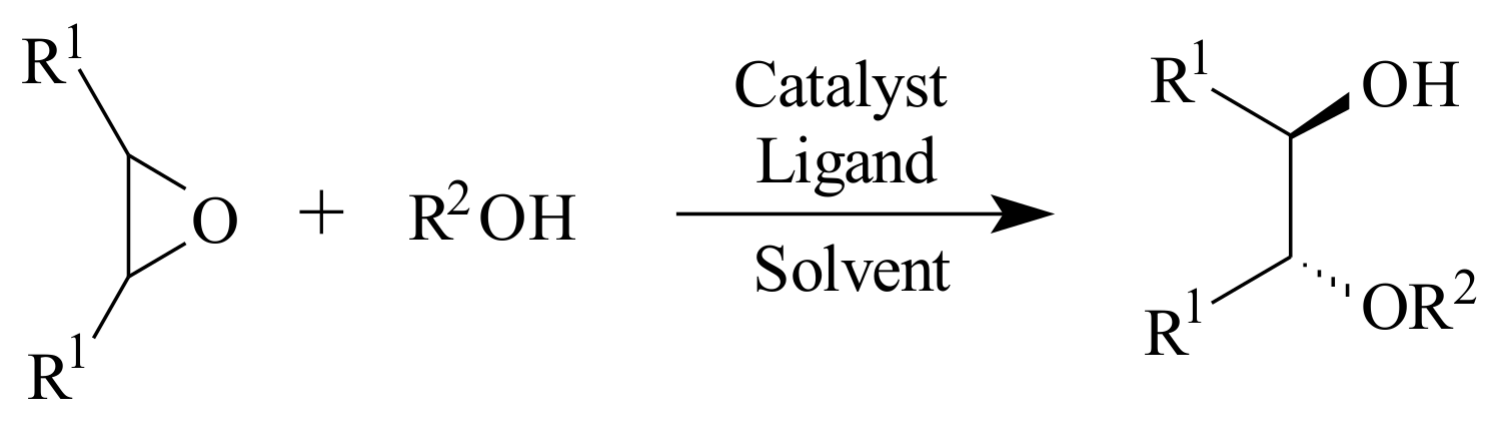
A generalised ring-opening reaction of *meso*-epoxides by alcohols.

Chiral *β*-(amino)alcohols are part of many biologically active compounds and chiral auxiliaries used in the enantioselective reactions. Catalytic enantioselective synthesis of these chiral compounds relies on the asymmetric ring opening of *meso*-epoxides [109]. The major problem in the nucleophilic addition of amines to epoxides catalysed by Lewis acids is the compatibility problems between the Lewis basic amine and the Lewis acid catalyst, which tend to coordinate to one another irreversibly. Crotti et al. [107,110,111] have brought a solution to this problem, in that lanthanide triflates, such as Yb(OTf)_3,_ are effective catalysts for the aminolysis of 1,2-epoxides, because they are able to exert their exceptional Lewis acidity even in the presence of the amine [112]. A generalised ring-opening reaction of a *meso*-epoxide using alcohol as a nucleophile is shown in Scheme 16. The nucleophile could also be an amine, a thiol or an indole.

In the catalysis for epoxidation of (1*R*)-(+)-*α*-pinene using the molybdenum (VI) catalyst **48** with *t*-BuOOH, Valente et al. [55] came across the ring-opening activity of *α*-pinene oxide (which is the product by itself) in campholenic aldehyde (8.6%) and epoxy campholenic aldehyde (50.2%). Similarly, using (1*S*)-(−)-*α*-pinene, the catalyst yielded 9.8% campholenic aldehyde and 58.1% epoxy campholenic aldehyde. The inadvertently obtained reaction products led them to test the activity of catalyst **64** on the ring opening reaction of *α*-pinene oxide, which resulted in 20% campholenic aldehyde and 39% epoxy campholenic aldehyde with 81% conversion of the *α*-pinene oxide.

Schneider et al. [108] established the first catalytic enantioselective addition of aliphatic alcohols to *meso*-epoxides, employing a novel scandium-bipyridinyl-complex with excellent ee and good c.y.’s. The α,α’-bipyridinyl diol **148** was used together with Sc(OTf)_3_ in 10 mol% in the suitable solvent (for their reaction) CH_2_Cl_2_ at room temperature. They used the *meso*-epoxides: *ci*s-stilbene, *cis*-1,2-naphthylethylene, *cis*-3,3′-dimethylstilbene, cyclohexene and *cis*-2-butene in both alcoholysis and aminolysis reactions. For the alcoholysis reaction; methanol, ethanol, *n*-butanol, 3-propenol and *p*-methoxybenzyl alcohol and for the aminolysis; aniline, *N*-methyl aniline, *p*-anisidine and *n*-butoxyamine were used. The alcoholysis yielded 75–93% c.y. with 49 to 98% ee, while the aminolysis resulted in 76 to 96% c.y. with 54 to 97% ee. Generally, the ee’s of aromatic epoxides were higher than the aliphatic epoxides in both alcoholysis and aminolysis reactions. Decreasing the catalyst load to 5 mol% decreased the ee from 93 to 90% and the c.y. from 95 to 89% for *cis*-stilbene in the aminolysis of the epoxide. *N*-methyl aniline showed the highest ee (97%). Conversion of the bipyridinyl diol to its bis-*O*-methyl ether led to a complete loss of enantioselectivity both in the alcoholysis and aminolysis of the *meso*-epoxides. It is therefore essential to have free hydroxyl groups for the formation of a highly enantioselective scandium-bipyridinyl-complex.

**Figure 30:**
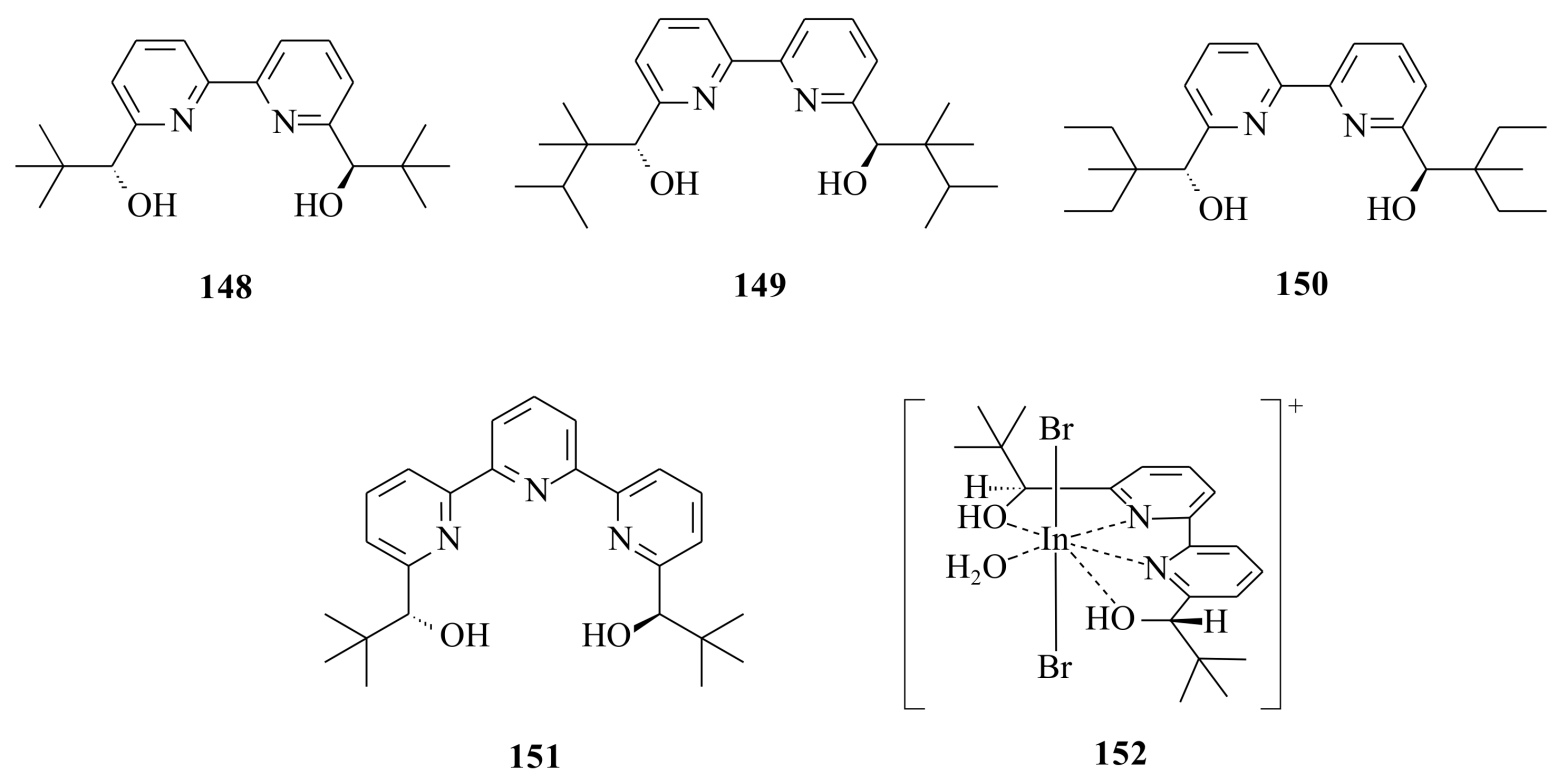
Pyridinyl alcohols with a di- and tripyridine backbone.

With the focus on important intermediates and building blocks in organic synthesis [113], Azoulay et al. [109] used the bipyridinyl diol ligand **148** (Figure 30) in the catalytic asymmetric ring opening of *meso*-epoxides in Sc(DS)_3_ using the environmentally friendly solvent water for the first time. The *meso*-epoxides of *cis*-stilbene, *cis*-4,4′-dimethylstilbene, *cis*-1,6-diphenyl-3-hexene, *cis*-5-decene, and *cis*-1,2-dinaphthylethylene were used for the ring-opening aminolysis with aniline. Good c.y.’s (61–89%) with moderate to good ee’s (60–96%) with the highest (89%) c.y. being for *cis*-stilbene and *cis*-5-decene (in aniline) and the highest ee (96%) for *cis*-stilbene in *N*-methylaniline were obtained. Aminolysis of *cis*-stilbene was also performed with *O*-anisidine, *α*-naphthylamine and 1-amino-4-bromohaphthalene. The catalyst Sc(DS)_3_ was used in 1 mol% and the ligand in 1.2 mol% during the aminolysis reaction. They also demonstrated that using 1 mol% catalyst and 1.2 mol% ligand **148** in the aminolysis of *cis*-stilbene, Sc(DS)_3_ yielded better (89% c.y. with 91% ee) in water than Sc(OTf) in CH_2_Cl_2,_ which resulted in 85% c.y. and 74% ee of the *β*-(amino)alcohol.

Optimisation of the aminolysis of *meso*-epoxides with respect to metal, ligand, solvent, catalyst loading, epoxide and amine component was performed by Mai and Schneider [112]. For the optimisation of the metal, the triflates [M(OTf)_3_] of the lanthanide series metals Sc, Y, Ce, Sm and Yb were used in 10 mol% with 12 mol% of the bipyridinyl diol **148** in CH_2_Cl_2_ at room temperature for *cis*-stilbene and cyclohexene oxides. The best results were obtained for Sc(OTf)_3_ in both epoxides. For ligand optimisation, the bipyridinyl diol **148** was modified having the intention to increase torsion angles between the pyridine rings by substituting methyl groups in position 2 of the pyridine ring (**148**), increasing the steric bulk of the *t*-Bu group at the chiral centres (**149** and **150**) and also a tripyridine backbone (**151**) (Figure 30). All of them were tested for their catalytic activity with Sc(OTf)_3_ 10 mol%, 12 mol% of each of the pyridinyl alcohols in CH_2_Cl_2_ at room temperature in the aminolysis of *cis*-stilbene and cyclohexene oxides. However, none of them yielded as excellent results as ligand **148**. The solvent optimisation involved CH_2_Cl_2_, ClCH_2_CH_2_Cl and CHCl_3_, where CH_2_Cl_2_ was found to be the best solvent in terms of yield. Dimethylformamide (DMF), CH_3_CN and THF coordinate to the Lewis acid and shut down the catalytic activity, while toluene and diethyl ether coordinate weakly to give high product yields with no enantioselectivity [114]

The optimisation of catalyst loading for the reaction with *cis*-stilbene oxide was done at catalyst loadings of 10, 5, 2 and 1 mol% and the product ee’s were 93, 90, 80 and 71% respectively. The chemical yield did not show a significant change. Therefore, the better catalyst load is 10 mol%. Several epoxides from both aromatic and aliphatic functionalities were tested in the optimised reaction condition and better results were obtained for aromatic *meso*-epoxides. Sterically hindered anilines such as *N*-methyl aniline and anilines with electron-withdrawing substituents yielded better ee’s compared to the electron-donating and non-sterically hindered anilines in *cis*-stilbene.

A combined Lewis acid-Brønsted acid acidity of the indium(III)-bipyridinyl diol complex was proposed by Nandakumar et al. [115] The indium-bipyridinyl-catalysed enantioselective ring-opening of *meso*-epoxides of *ci*s-stilbene, *cis*-1,2-naphthylethylene, *cis*-3,3′-dimethylstilbene, *cis*-4,4′-dimethylstilbene and *cis*-4,4′-dichloro stilbene in benzylthiol, phenylthiol, 4-methyl phenylthiol, ethylthiol, *n*-butylthiol and 1-methyl butylthiol resulted in 1,2-mercapto alcohols in good yields (67–91%) and moderate to good enantioselectivities (85–96%). The optimised reaction condition utilised 10 mol% of InBr_3_, 11 mol % of the bipyridinyl diol **148** in CH_2_Cl_2_ at room temperature. The X-ray crystallography revealed that, in the [InBr_2_-**148**·H_2_O]^+^ complex, the hydroxyl hydrogens remain intact and the bipyridinyl is four coordinated to the indium metal centre. The hydroxy protons in the pyridine ring make a hydrogen bond to the thiol, leading the metal centre to the thiol for activation (see **152**).

Scandium-bipyridinyl-catalysed enantioselective alcoholysis of *meso*-epoxides was optimised with respect to metal, ligand, solvent, catalyst loading, alcohol, and epoxide components [114]. *cis*-stilbene and cyclohexene oxides with the bipyridinyl diol **148** are used in the optimisation process. The triflates of Sc, Y, La, Ce, Nd, Sm, Tb, Tm, Yb, Cu and Zn were tested for optimisation of the metal in the alcoholysis of *cis*-stilbene in *p*-methoxybenzyl alcohol (PMBOH) and cyclohexene in allyl alcohol and best yields were obtained for Sc(OTf)_3_ (82% c.y. and 97% ee) as in the case of the aminolysis. The bipyridinyl diol ligands **148**–**151** were tested for *cis*-stilbene oxide ring-opening with allyl alcohol; however, no significant variation in both selectivity and chemical yield was observed, except for **151**, which did not give rise to an enantioselective catalyst. 1,2-Dichloroethane was found to be the second choice of solvent after CH_2_Cl_2_. Optimisation of catalyst loading was performed in 15, 10, 5, 2 and 1 mol% of Sc(OTf)_3,_ which yielded 98%, 97%, 94%, 90% and 73% within 0.5, 0.5, 5, 8, and 9 days, respectively. Consequently, the appropriate catalyst load is 10 mol%; however, it can be reduced to 5 mol% as there is no significant variation in the yield except prolonged reaction time. Various solvents, including methanol, ethanol, PMBOH, allyl alcohol, *n*-butanol, *iso*-propanol and 3-propargyl alcohol were used in the optimisation with *cis*-stilbene and PMBOH yielded better (82% c.y. and 97% ee) 1,2-diolmonoether. The aromatic epoxides undergo alcoholysis with PMBOH in a better yield than aliphatic epoxides. The *α*-branched allylic epoxide resulted in the highest ee in the aliphatic epoxide category.

The X-ray crystallography of Y(OTf)_3_-**148** complex shows the bipyridinyl being slightly twisted along its own axis (19.1°) and coordinated in a tetracoordinate fashion with two triflate groups and two water molecules bound to the metal centre. The ESI-MS spectra of the Sc(OTf)_3_-**148** complex in CH_2_Cl_2_/CH_3_CN solution confirmed the presence of [M-H]^+^ species, which could be taken as proof for the presence of the hydroxy group in the complex. Using a catalytic amount (1 mol%) of Sc(DS)_3_ and the chiral bipyridinyl diol ligand (*S*,*S*)-**148** (1.2 mol%) resulted in ring-opening desymmetrisation of *meso*-epoxides with aromatic amines and indole derivatives in water as sole solvent [116]. The reaction proceeded much faster in water than in dichloromethane. Aromatic epoxides *cis*-stilbene, *cis*-4,4′-dimethylstilbene, *cis*-1,2-dinaphthylethylene oxides resulted in better c.y. (75–89%) and ee (86–96%) compared to the aliphatic epoxides *cis*-1,6-diphenyl-3-hexene, and *cis*-5-decene oxides (61–89% c.y. and 60–71% ee). The aromatic amines used in the aminolysis include aniline, *o*-anisidine, *α*-naphthylamine and 1-amino-4-bromo naphthalene.

It was also reported that Sc(DS)_3_-(*S*,*S*)-**148** in water resulted in better c.y., ee’s and reactivity than Sc(OTf)_3_-(*S*,*S*)-**148** in dichloromethane. Ring-opening desymmetrisation of *meso*-epoxides *cis*-stilbene, *cis*-4,4′-dimethylstilbene, *cis*-4,4′-dibromostilbene oxides with indole, 5-methoxyindole, 5-methylindole, 2-methylindole and 5-bromo indole in the presence of 5 mol% Sc(DS)_3_ and 6 mol% ligand (*S*,*S*)-**148** resulted in good yields (56–62%) and moderate to good ee’s (86–93%). Similarly, the reaction is much faster in water than in dichloromethane.

Aminolysis of aromatic and aliphatic *meso*-epoxides was also efficiently catalysed by 10 mol% In(OTf)_3_-**148** catalyst in dichloromethane at room temperature to result in good yields and moderate to high enantiomeric excess (up to 98%) of 1,2-(amino)alcohols [117]. Electron-deficient and sterically hindered anilines gave rise to high ee’s (up to 98%), thereby maintaining the c.y. at a good level. The aliphatic epoxides were ring-opened with low enantioselectivities (31–36%) and high chemical yields (82–91%) just as in the scandium-bipyridinyl catalysed process [108,112,114]. Analysis of the crystal structure of InBr_3_-bipyridinyl catalyst (as it was not possible to get crystals of In(OTf)_3_-**148** complex) revealed a pentagonal bipyramidal coordination geometry around the metal centre with the hydroxyl protons intact to the complex that closely resembles the related Sc-bipyridinyl complex. This is believed to be the reason for both complexes resulting in the same absolute configuration products.

In the previous sections, we have presented the Sc(OTf)_3_ and In(OTf)_3_-catalysed process for the addition of alcohols, amines and thiols to *meso*-epoxides in the presence of the bipyridinyl diol **148** and its (*S*,*S*)-isomer, which resulted in 1,2-diol monoethers, 1,2-(amino)alcohols and 1,2-(mercapto)alcohols. Tschöp et al. [118] further extended this protocol to the addition of phenyl selenol to aromatic *meso*-epoxides giving rise to 1,2-seleno alcohols in good yield and up to 94% ee, which complements the Yang et al.’s [119] process. Stilbene oxide reacted with phenyl selenol in the presence of 10 mol% Sc(OTf)_3_ and 10 mol% of ligand **148** in CH_2_Cl_2_ at room temperature for 12 h to yield 60% 1,2-selenoalcohol with 93% ee and 20% deselenated alcohol. As the deselenated side product was believed to be produced via a radical process removing oxygen from the reaction mixture and undergoing the reaction in the dark increased the 1,2-selenoalcohol yield to 77% after eight hours with no deselenated product. Reactions with and without light and oxygen were done with *meso*-epoxides *cis*-4,4′-fluorostilbene, *cis*-4,4′-chlorostilbene, *cis*-4,4′-bromostilbene and *cis*-3,3′-meth ylstilbene. The results obtained showed clearly that the deselenated product increased in the presence of oxygen and light in all cases. Based on this result, a deselenation reaction of *cis*-4,4′-difluorostilbene and PhSeH via direct *meso*-epoxide ring-opening in the presence of Sc(OTf)_3_ and bipyridinyl diol **148** in CH_2_Cl_2_ at room temperature in 24 h resulted in 83% of the deselenated product. The same reaction resulted in 25% yield without catalyst and 81% only in the presence of the bipyridinyl diol ligand **148**. Therefore, it is more likely that the deselenation reaction would be assisted by the bipyridinyl diol ligand in the abstraction of the selenol proton that is assumed to be the first step in the radical deselenation process. Cyclohexene oxide, however, did not show a deselenated product even in the presence of both oxygen and light.

The square pyramidal Cu(DS)_2_-(*S*,*S*)-**148** complex and the pentagonal bipyramidal structure Sc(DS)_3_-(*S*,*S*)-**148** complex resulted in opposite yet nearly similar enantioselectivities in asymmetric ring-opening reactions of *meso*-epoxides; *cis*-stilbene oxide, *cis*-4,4′-dimethyl stilbene oxide, *cis*-4,4′-dibromostilbene oxide, *cis*-1,2-dinaphthylethylene oxide and with indoles 5-methoxyindole, 5-methylindole, 5-bromoindole, 2-methylindole and indole [120]. 10 mol% of catalyst and 12 mol% of ligand were used in water as sole solvent at room temperature for 22 h to result in 48 to 82% c.y. and 85 to 96% ee of the (*S*,*S*)-product by Cu(DS)_2_ and 46 to 80% c.y. and 84 to 95% ee of the (*R*,*R*)-product by Sc(DS)_3_. Using organic solvents such as CH_2_Cl_2_, THF, toluene etc. and catalyst Cu(OTf)_2_ or Sc(OTf)_3_-(*S*,*S*)-**148** resulted in much lower yields of the product. This is therefore one way of obtaining the two enantiomers of the alcohol by using the appropriate metal with the bipyridinyl diol ligand (*S*,*S*)-**148** complex.

The ring opening reactions of *meso*-epoxides with aniline and indole derivatives in the presence of Zn(II) and Cu(II) surfactant-type catalysts resulted in the corresponding products in moderate to high ee’s and good to excellent c.y.’s were reported by Kokubo et al. [121] It is well known that most catalytic organic reactions take place in organic solvents as a result of the insolubility of most organic compounds in water. However, surfactant-type catalysts were becoming solutions to such problems. Alkyl epoxides and stilbene derivative oxides were made to react with aniline derivatives in the presence of 5 mol% of Cu(DS)_2_ and/or Zn(DS)_2_ catalysts and 6 mol% bipyridinyl diol ligand (*S*,*S*)-**148** in water at room temperature to yield product in moderate to good (44–95%) selectivity and good to excellent chemical yield (43–100%), respectively. The same reaction was made in CH_2_Cl_2_ using Zn(DS)_2_ that resulted in 70 to 90% ee with 10 to 92% c.y. Cu(DS)_2_ was not as effective in CH_2_Cl_2_. The Zn(DS)_2_ and Cu(DS)_2_ with (*S*,*S*)-**148** in water were highly reactive and with higher enantioselectivity yields than the Zn(OTf)_2_ and Cu(OTf)_2_ with (*S*,*S*)-**148** in CH_2_Cl_2_.

**Figure 31:**
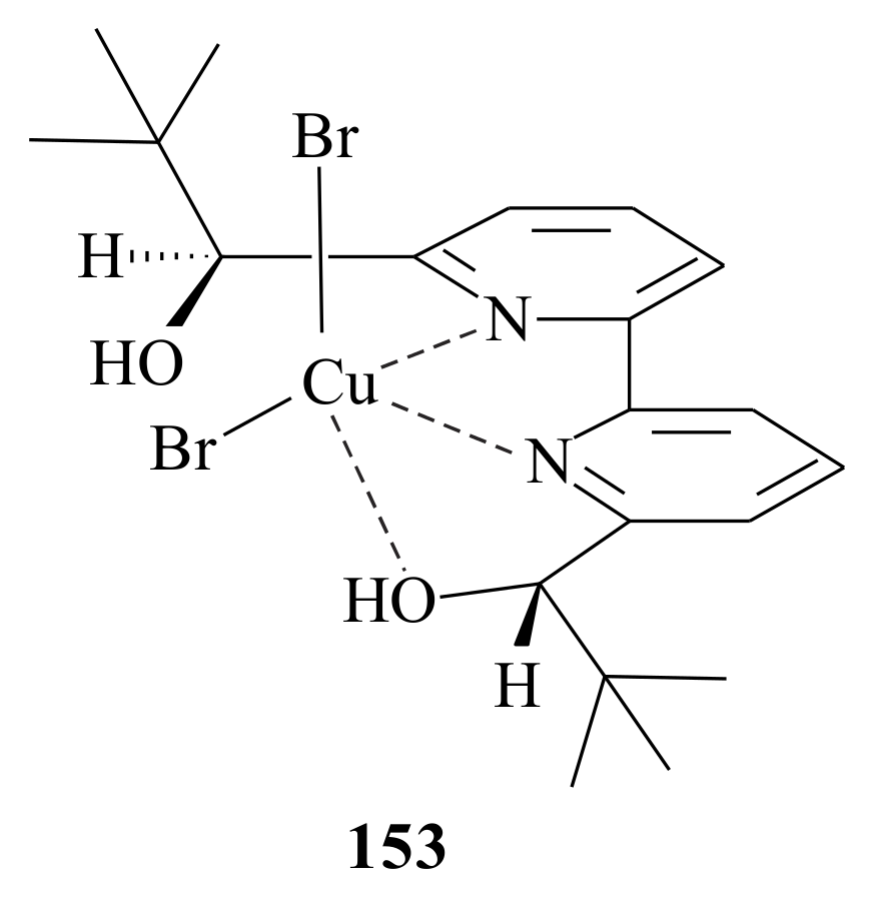
Tridentate coordination of an α,α’-bipyridinyl diol with Cu.

The indole derivatives resulted in good to excellent ee’s in water using Sc(SD)_3_ and Cu(SD)_2_ catalysts and (*S*,*S*)-**148**. In CH_2_Cl_2,_ only Cu(OTf)_2_ resulted in product in moderate yield and high selectivity. The rate in CH_2_Cl_2_ and water was nearly the same; however, the selectivity was higher in water than in CH_2_Cl_2_. In all cases, Sc(III) resulted in the opposite enantiomer than Cu(II) or Zn(II) catalysts. The X-ray structure of [CuBr_2_·(*S*,*S*)-**148**] shows that the bipyridine coordinated with only one of the hydroxyl groups and the two nitrogen atoms with the copper metal. The second hydroxyl group therefore is most likely used to coordinate to the substrate as it is shown in complex **153** (Figure 31).

Careful examination of the aminolysis or alcoholysis of *meso*-epoxides by the transition metal complexes supported by the bipyridinyl diol **148**, its isomer (*S*,*S*)-**148**, and the modified versions of **148** reveals the use of limited or a few bipyridinyl diols only. Even though the yield obtained by the Mo(VI) complex **64** is low, it indicated the fact that pyridinyl alcohols other than the bipyridinyl diol **148** or structurally-related diols could be used in the catalysis of the reaction. In addition to this, complex **153** revealed that the second alcohol function on the bipyridinyl diol does not coordinate to the metal, and therefore there seems to be a high possibility to use pyridinyl mono alcohols with varied structures having different steric and electronic effects to get the best ee and c.y.’s in this homogeneously catalysed reaction.

### 2.9. Aldol Reaction

Carbon-carbon forming reactions are very useful in organic synthesis. An asymmetric aldol reaction is one of the most useful carbon-carbon bond-forming reactions to afford optically active β-hydroxy carbonyl compounds. Recently, several asymmetric aldol reactions have been developed to pursue efficiency in obtaining these chiral compounds [122]. In this section, we shall discuss the asymmetric aldol reactions that involve the pyridinyl alcohols or their derivatives.

The major problem with catalytic enantioselective aldol reactions of silicon enolates in aqueous media is the competitive ligand exchange between a chiral ligand and water, which promotes achiral free Lewis acid-catalysed pathways [122]. However, the water-tolerance nature of the lanthanide trifluoromethanesulfonates (Ln(OTf)_3_) made them useful Lewis acid catalysts in aqueous media [123]. In addition, the rare earth triflates are strong Lewis acids because of the hard character of their metal cations and the strong electron-withdrawing effect of the trifluoromethanesulfonyl group [124]. The advantage of these reactions compared to the conventional Lewis acid-mediated reactions in dry organic solvents is the ease to remove water from the solvents, substrates and catalysts [125]. As water is used as a ‘solvent’ in the metabolic process of living organisms, studies on organic reactions in aqueous media may lead to a real understanding of life and nature; particularly, chiral catalysts, which work well in aqueous media may become models of enzymes [124].

**Scheme 17:**
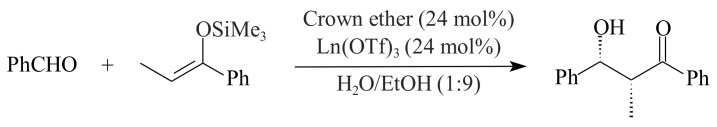
A generalised aldol reaction catalysed by the lanthanide triflates.

The first demonstration of this kind of reaction was made by Kobayashi in Yb(OTf)_3_-catalysed aldol reactions of silyl enol ethers with formaldehyde in aqueous THF [126]. A generalised aldol reaction is shown in Scheme 17.

**Figure 32:**
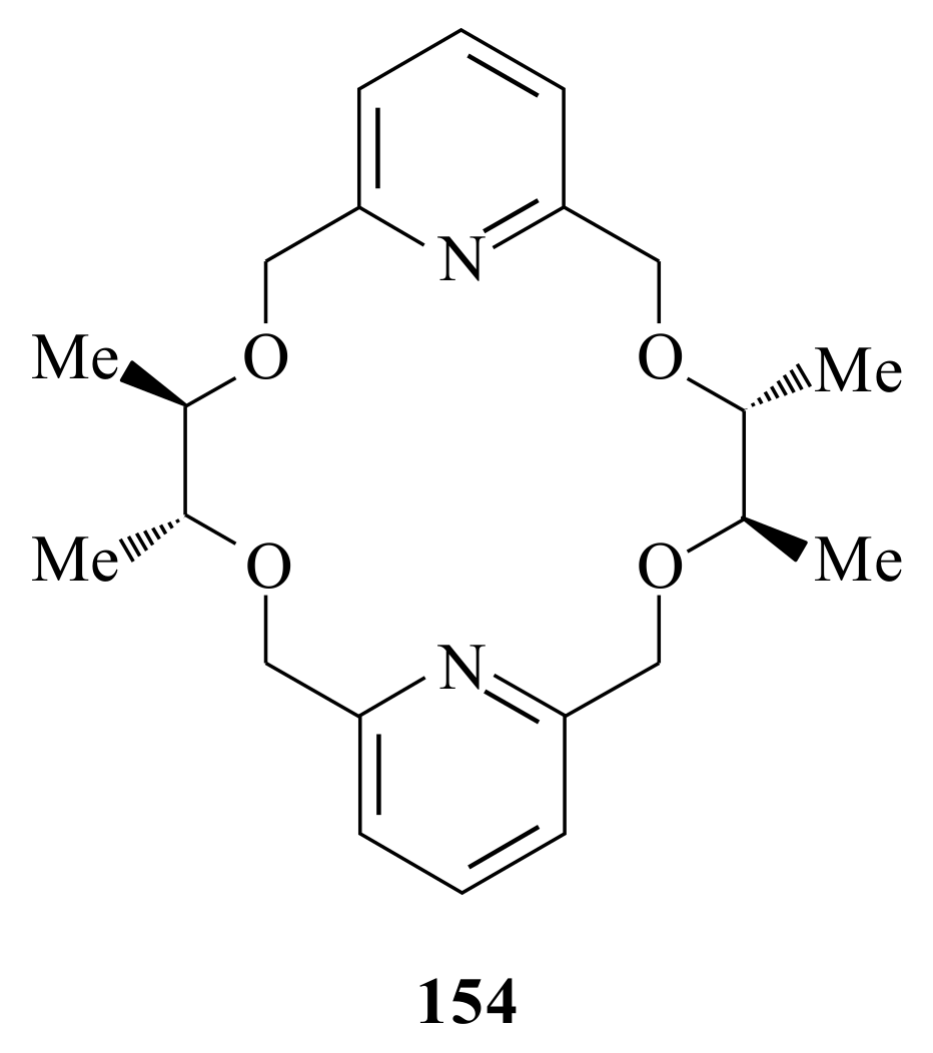
Pyridino-crown-ether synthesized from 2,6-pyridine dimethanol.

The use of pyridino-crown-ether **154** (Figure 32), synthesized from 2,6-pyridine dimethanol, in the catalytic asymmetric aldol reactions catalysed by lanthanide triflates in aqueous media was reported by Kobayashi et al. [125] An adduct of aldol with 85% yield in 82% (*syn*/*anti*) and 78% ee was obtained by reacting benzaldehyde with silylenol ether by using the catalyst 10 mol% Pr(OTf)_3_ and 12 mol% **154** in H_2_O/EtOH (1/9, v/v) at 0 °C in 18 h.

The same reaction in pure EtOH resulted in 51% yield with 70% de and 23% ee. In DCM (dichloromethane), the yield decreased by as much as 3% with 28% de and 22% ee. Using the reaction condition in Scheme 17, the triflates of the lanthanide series metals Sc, Yb, Ho, Y, Dy, Tb, Gd, Eu, Sm, Nd, Pr, Ce and La were used for catalysis. The yield obtained is correlated to the diameter of the cation. The large cation diameter metals such as La, Ce, Pr and Nd resulted in high enantio- and diastereo-selectivity, while the small diameter cations Sc and Yb did not show enantioselectivity at all. Maximum enantio- and diastereo-selectivity was obtained for Ce (86% de and 82% ee). The reason for the difference in the selectivity is the sufficient coordination ability of those cations with large diameters with the crown ether in the proposed mol% ratio of metal to crown ether.

Manabe et al. [122] achieved chiral Lewis acid catalysed enantioselective hydroxymethylation of silicon enolates for the first time. The reaction of 10 equivalents of formaldehyde and the silicon enolate (see Scheme 17) in the presence of 20 mol % Pr(OTf)_3_ catalyst and 24 mol% of the crown ether ligand **154** in H_2_O/THF (THF as co-solvent) at 30 °C in 18 h yielded 91% c.y. with 48% ee of the hydroxymethyl. Using the same reaction condition, a series of *mono*-, *di*- and *tri*-substituted aromatic and aliphatic silicon enolates were tested and moderate to very good yields (40–92%) with poor to moderate ee’s (14–54%) were obtained. Using ethanol as co-solvent showed the best enantioselectivity, although a slight decrease in the chemical yield is observed.

Detailed study and further improvement of the asymmetric aldol reactions in aqueous media are described by Hamada et al. [124]. They further investigated the effects of the metal cation diameter, the better solvent for the reaction, effect of electron-withdrawing and electron-donating substituents on the *para*-position of the pyridine ring in the crown ether and structure of complex. In addition to this, the design of the crown ether and a model transition state for the complex Pr^3+^-crown ether were proposed. They began their study with the effect of metals on the catalysis of silyl enol ethers and came across with similar results as before, i.e., large diameter cations La, Ce, Pr, and Nd resulted in the aldol adduct with high diastereo- and enantioselectivities, while the small diameter cation metals Dy, Y, Ho, Yb and Sc gave lower selectivities. Their investigation revealed that the large diameter cations strongly bind to the crown ether, while the small diameter cations bind weakly.

**Scheme 18:**
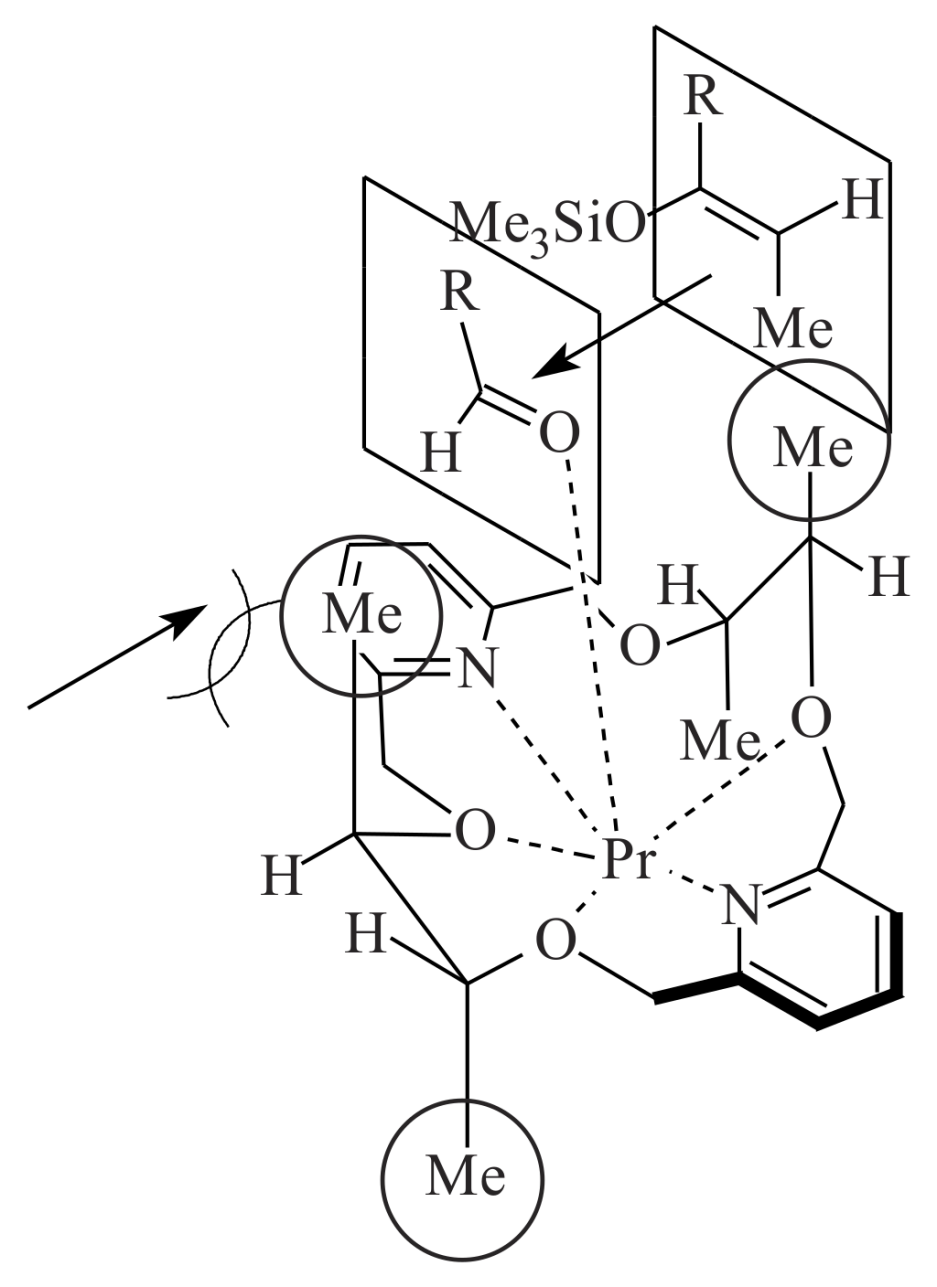
A model transition state for the aldol reaction of silyl enol ether in Pr^3+^-**154** complex.

The effect of the electron-donating (MeO) and electron-withdrawing substituents (Br) like in the para-position of the crown ether was revealed by decreasing the reactivity without affecting the selectivity of the catalyst significantly. The same level of diastereo- and enantioselectivities for the large diameter cations La, Ce and Pr was observed in the electron-withdrawing substituted crown ethers. This is because the electron-withdrawing bromine will decrease the binding ability of the crown ether and a couple of cations will be available in the reaction solution. This therefore leads to an achiral catalysis and therefore low selectivity. In the case of electron-donating substituent, the high electron density on the nitrogen of the crown ether will increase the binding ability of the metal cations and therefore a decrease in the Lewis acidity of the crown ether-metal complex [124]. Regardless of the ratios between 1/3–1/27, H_2_O/EtOH resulted in good selectivities, even though the increase in the amount of water decreased the selectivity slightly. In relation to this, they examined the effect of water and came up with the most probable suggestion that water aids in creating an effective chiral environment for the Pr^3+^-**154** complex. They also investigated whether the structure of the crown ether-rare earth complexes affects the selectivity; however, they found that almost all complexes have similar structure and the structure of the complex does not affect the selectivity. They studied the structure by considering the crystal of the complex [Pr(NO_3_)_2_-**154**]_3_ [Pr(NO_3_)_6_] as it was not possible to get the crystal of Pr(OTf)_3_. The selectivity differences depended on the binding ability and catalytic activity of complexes. Therefore, in order to attain high selectivity in the asymmetric aldol reaction, the design of a chiral crown ether, which complexes strongly with RE(OTf)_3_ and does not decrease the Lewis acidity of RE(OTf)_3_, is essential (RE = rare earth). The model transition state for the aldol reaction is shown in Scheme 18 [124].

A series of aldol reactions of aromatic and aliphatic *α*,*β-*unsaturated aldehydes with silyl enol ethers were performed by Hamada et al. [124], which resulted in good to high yields and diastereo- and enantioselectivities. In the aldol reactions using the silyl enol ether derived from a thioester, the addition of 2,6-di-*tert*-butylpyridine effectively enhanced the yields of aldol adducts in aqueous media.

**Figure 33:**
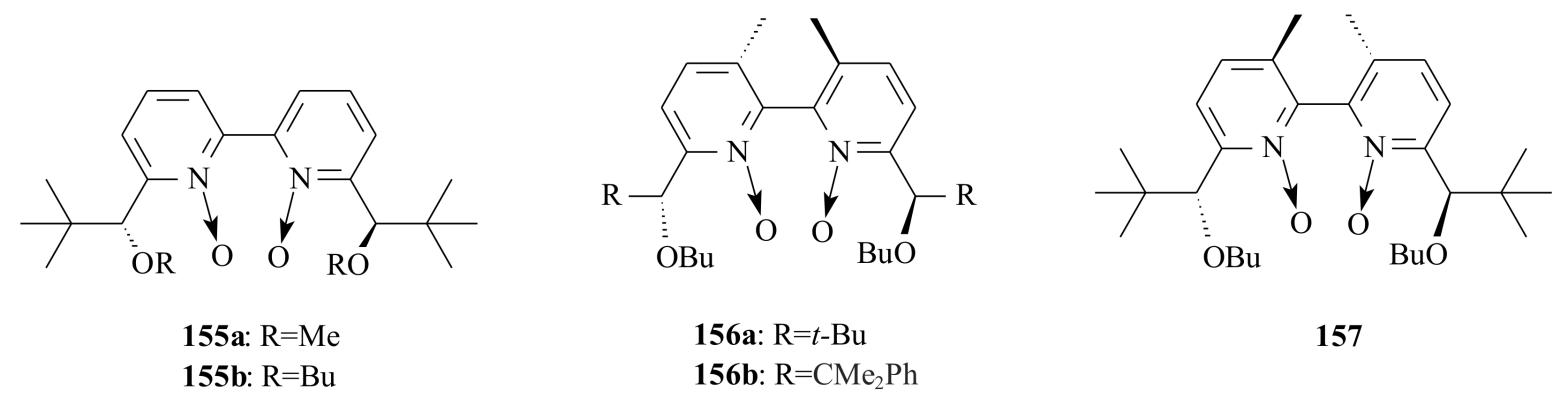
Chiral pyridinyl *N*-oxide ethers as catalysts in enantioselective aldol additions.

The use of chiral pyridinyl *N*-oxide ethers **155**–**157** (Figure 33) as catalysts in the enantioselective aldol additions to ketones was reported by Denmark and Fan [127]. The very reactive nature of trichlorosilyl enolate of methyl acetate with aldehydes resulted in the less enantioselective product.

The strong chelation of the trichlorosilyl fragment renders the enol addition essentially irreversible. In spite of all these challenging features of the silyl enolate, Denmark and Fan [127] made the aldol addition of trichlorosilyl enolate of methyl acetate with acetophenone using the chiral pyridinyl *N*-oxide ethers as catalysts, which they believe will function as Lewis basic promoters by coordinating and ionising the chloride ions. The reaction took place at room temperature using 10 mol% of the catalysts (**155**–**157**) in CH_2_Cl_2,_ followed by treatment with aqueous NaHCO_3_. The result of the reaction is presented in Table 10. The yields of the reaction clearly reveal the fact that the sense of asymmetric induction is dominated by the configuration of the chiral axis and that the 6,6′-stereocenters play a secondary role. This is seen clearly by the negligible influence of the bulky phenyl substituted catalyst **156b**. A study of a broad substrate generality on the catalytic activity was made on aromatic, heteroaromatic, olefinic, acetylenic, aliphatic, branched and linear ketones.

The aromatic ketones gave the highest selectivities (80–86% ee), while the olefinic ketones were the poorest performers, even less than the aliphatic ketones (20–41% ee). Therefore, the bipyridinyl *N*-oxide ethers function as catalysts in the enantioselective aldol additions of hindered and enolisable ketones, as well as those with multiple reactive sites.

Due to the moderate yield and enantioselectivity of the previous catalytic asymmetric hydroxy methylation of silicon enolates [122] in aqueous solution, Ishikawa et al. [128] further continued their investigation and came up with a new catalyst that resulted in a better yield and selectivity. Optimisation of the reaction condition was made by the reaction of the commercially available formaldehyde and the trimethylsilyl enolate of phenyl propanone. The better result (80% c.y. and 90% ee) was obtained by using five equivalents of formaldehyde in 0.36 M concentration, 10 mol% of the Sc(OTf)_3_ catalyst and 12 mol% of the bipyridinyl diol (*S*,*S*)-**148** in H_2_O/DME (1,2-dimethoxyethane) in 1:9 ratio at −20 °C in 24 h. Using 5 mol% of the catalyst reduced the yield to 67% c.y. and 86% ee. Substrate generalisation was made by reacting formaldehyde (using the optimised reaction condition) with aliphatic and aromatic silyl enolates, some of them possessing ethyl or siloxy groups at *α*-position. The substrates resulted in poor to excellent yields (24–90%), with moderate to good enantioselectivities (60–94% ee). In some cases, addition of 2,6-di-*tert*-butylpyridine slightly improved the yield. Formaldehyde reacted to the enolates that possess *α*-substituent from the same face regardless of the substituents at *α*-potion. The X-ray crystal structure of the ScBr_3_-(*S*,*S*)-**148** revealed a pentagonal bipyramidal structure in which the hydroxy groups of the bipyridinyl diol coordinate to Sc^3+^ in a tetradentate manner, which is in agreement with Nakamura et al.’s [115] [InBr_2_-**148**·H_2_O]^+^ crystal structure **152**. This structure is perhaps a key for the high enantioselectivity.

Kobayashi et al. [129] added the water-unstable Bi(OTf)_3_ in a “water compatible Lewis acid” category upon using the basic bipyridinyl diol ligand (*S*,*S*)-**148**. Bi(OTf)_3_ became an active and enantioselective catalyst in the asymmetric hydroxymethylation of silicone enolates with an aqueous solution of formaldehyde. During optimisation of the reaction conditions, by reacting aqueous formaldehyde with trimethyl silyl enolate of phenyl propanone, the highest yield (92%) and enantioselectivity (93%) were obtained upon using 10 mol% of Bi(OTf)_3_ and 30 mol% of (*S*,*S*)-**148** in H_2_O/DME (1/9) at 0 °C in 4 h. Decreasing the catalyst loading to 1 mol%, the ligand to 3 mol% and undergoing the reaction for 12 h at 0 °C, increased the yield to 93%, while decreasing the enantioselectivity to 91%. Further reduction of the catalyst and ligand load to 0.5 mol% and 1.5 mol% resulted in 76% c.y. and 90% ee, respectively. The substrate generalisation reactions were performed using 1 mol% of Bi(OTf)_3_ and 3 mol% of (*S*,*S*)-**148** in H_2_O/DME (1/9) at 0 °C . Substrate generalisation was made for the silyl enolates of aliphatic and aromatic compounds possessing *α*-substituted ethyl and siloxy groups that resulted in good to excellent yields (59–93%) and moderate to good enantioselectivities (77–95% ee). The addition of 6,6′-*tert*-butyl bipyridine reduced the rate of decomposition of some silicon enolates. The highly water-unstable Bi(OTf)_3_ catalyst was stabilised by the presence of the basic ligand. The pentagonal bipyramidal structure with the tetradentate ligand occupying four of the equatorial sites of BiBr_3_-(*S*,*S*)-**148** was found to be similar to the single crystal X-ray structure of the complexes Sc(III)Br_3_ and [InBr_2_-**148**·H_2_O]^+^ (**152**). It is quite interesting to see Sc and Bi having different ionic diameters forming similar complexes with (*S*,*S*)-**148**.

The relentless effort of Kobayashi et al. [130] made them undergo catalytic asymmetric hydroxy methylation of silicone enolates using formaldehyde in water as sole solvent. The use of 10 mol% of Sc(DS)_3_ and 12 mol% of the bipyridinyl diol (*S*,*S*)-**148** in Triton X-705, at room temperature, resulted in good to high yield (81–81%) and high enantioselectivity (>90% ee) in most cases.

The use of 2 or 1 mol% Sc(DS)_3_ led to the same range in yield and enantioselectivity to that of 10 mol% Sc(DS)_3_. Aliphatic and substituted aromatic silicon enolates with *α*-ethyl, -methyl, -*tert*-butyl and -*iso*-propyl substituted silicon enolates were involved in the substrate generalisation test. The relatively highly sensitive ketone-derived silicon enolates in water (compared to silyl enol ethers) reacted smoothly under the reaction conditions to afford the desired hydroxymethylated adducts in good yield and high enantioselectivity. Kobayashi et al. [130] applied this method in the synthesis of the artificial odorant (*S*)-2-(hydroxymethyl)-2,5-dimethylindane.

### 2.10. Allylic Alkylation

The significant contribution made by Kobayashi et al. [130] in this area is so valuable that it has shown the advantages of using bipyridinyl diols in the aldol reactions. However, the pyridinyl alcohol pool is so vast that there are still more alternative pyridinyl diols with varying substituents on the pyridine ring with respect to both steric and electronic factors. It is therefore worthwhile to consider more pyridinyl alcohols and further investigate the catalytic activity to come up in the aldol reaction with excellent c.y. and ee.

**Scheme 19:**
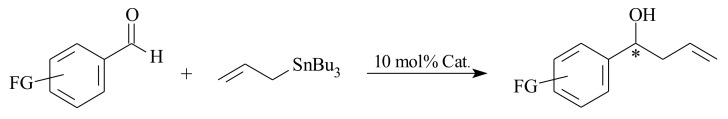
Catalytic asymmetric allylic alkylation of aldehydes.

The allylic alkylation reaction is a particularly useful catalytic process that enables the synthetic organic chemist to form carbon-carbon bonds, which is the backbone of organic synthesis. A significant number of catalysed allylic alkylations that involve the use of a variety of *P*,*P-*, *P*,*N-*, and *N*,*N*-based ligands, with *C_1_* as well as *C_2_* symmetry, have proven to induce high selectivity in the process [131,132]. In this section, we discuss the *N,O*-based pyridinyl alcoholato ligands in the catalytic enantioselective allylic alkylation reactions. A general equation for the catalytic asymmetric allylation of aldehydes is presented in Scheme 19.

Palladium-catalysed allylic alkylation using pyridinooxazolines (compounds with **158** as backbone) as ligands was reported by Bremberg et al. [131] Racemic 1,3-diphenyl-2-propenyl acetate reacted with dimethyl malonate through the addition of 2 mol% of [(η^3^-C_3_H_5_)PdCl]_2_ catalyst, 6 mol% of pyridinooxazolines **105b**, **106b** and **158** in the presence of *N,O*-*bis*(trimethylsilyl)acetamide (BSA) with a catalytic amount of KOAc and resulted in 90% ee (with **105b**), 95% ee (with **106b**) and 88% ee of the (*R*)-isomer (with **158**) [97]. The reason for having similar selectivities is probably that in the preferred conformation of the ligand, the *t*-Bu group is remote from the allyl group and therefore only has a minor influence on its position.

**Figure 34:**
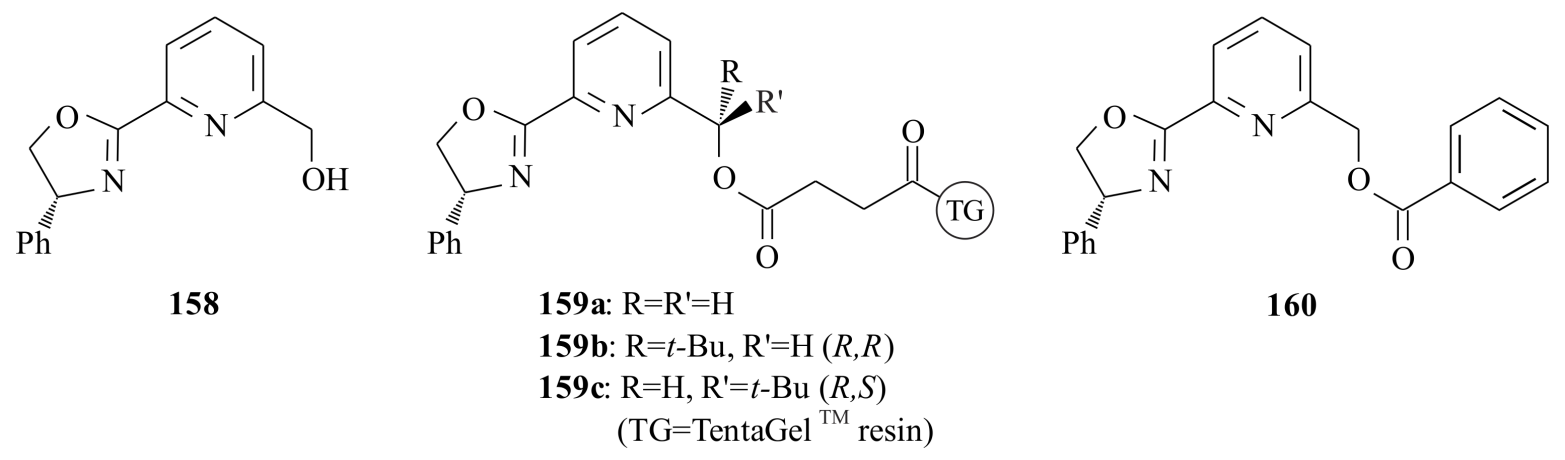
Unsupported and polymer-supported chiral pyridinooxazoline ligands.

The following year, Hallman et al. [133] prepared polymer-supported chiral pyridinooxazolines **159a**–**c**, using ligands **158**, **105b** and **106b** respectively, and assessed these in the palladium catalysed substitution of *rac*-1,3-diphenyl-2-propenyl acetate (Figure 34). Reagents bound to these new types of resins can be used in solvents with a wider range of polarity and are more easily analysed by spectroscopic methods in the solvent-swollen state. Using the same reaction conditions and catalyst as before, and the polymer-supported pyridinooxazolines **159a**–**c** ligands, they [133] obtained the (*R*)-configuration product in 60–100% c.y. with 80% ee in **159a**, 4% c.y. with 66% ee in **159b** and 3% c.y. with 61% ee in **159c**. For comparison purposes **160** was also investigated, 76–98% c.y. with 77% ee was obtained. In contrast to previous investigations [131,134,135], only minor differences in the enantioselectivity exhibited by diastereomeric ligands were observed in the present study. However, the low yields of products obtained using ligands **159b** and **159c** do not allow definite conclusions regarding the selectivity, which has previously been shown to depend on the conformational preferences of the ligands.

With regard to the mechanism of the Pd-catalysed enantioselective allylic alkylation, two steps are very important in order to induce enantiodiscrimination: (i) the activation of the allylic substrate by the Pd^0^-ligand complex with formation of the Pd^0^-olefin intermediate; and (ii) the attack of the nucleophile on the Pd-(η^3^-allyl) cation [132].

**Figure 35:**
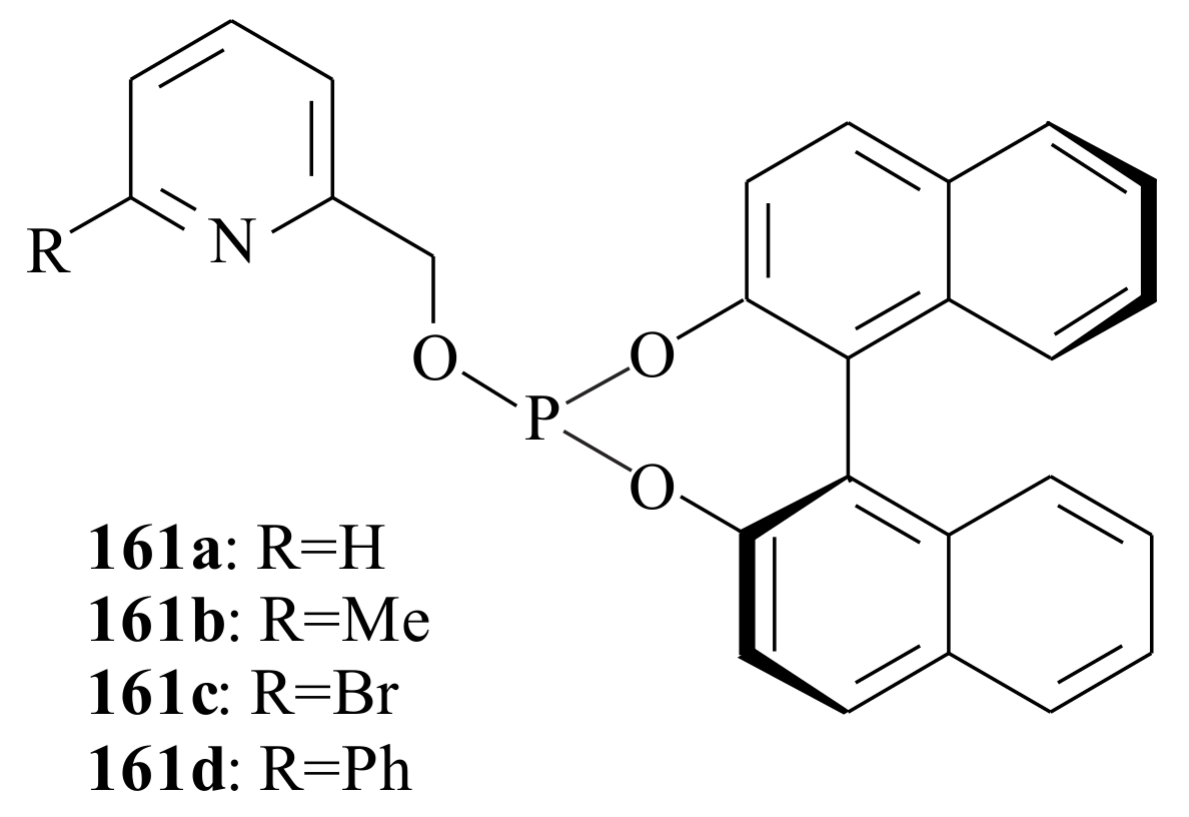
Pyridine-phosphinite ligands.

With the aim of gaining an insight into the possible correlation between the intermediate palladium and allyl isomer containing the chiral bidentate ligand and the enantiodiscrimination of the nucleophilic attack, Arena et al. [132] prepared pyridine-phosphinite ligand **161a** (from pyridinyl alcohols) and tested in the palladium(II)-catalysed enantioselective allylic alkylation of 1,3-diphenyl-1,2-propenyl acetate in dimethyl malonate (Figure 35). 1,3-Diphenyl-1,2-propenyl acetate was added to a dichloromethane solution of [Pd(η^3^-C_3_H_5_)**161a**]CF_3_SO_3_ and then added dimethyl malonate, *N,O*-bis(trimethylsilyl)acetamide and a catalytic amount of KOAc to obtain 95% of the racemic product. The lack of enantiomeric excess using **161a** was believed to be a consequence of the presence of the relatively small pyridine instead of the sterically bulky PPh_2_ that did not sterically hinder the allylic intermediates. Based on the NMR spectroscopy, it was possible to see the low steric demand of the pyridine moiety leading to the fast exchange of all the four possible configurational isomers of the Pd-allyl complexes present in solution.

Therefore, the number and the relative concentrations of the Pd-allyl conformers present in solution as intermediates in the allylic alkylation are factors determining the enantioselectivity of the process.

In light of their earlier results, Arena et al. [136] have increased the steric hindrance of the pyridine-phosphite ligands by introducing different substituents on the 6-position in the pyridine moiety (**161b**–**d**) (Figure 35). Furthermore, with the aim of obtaining a greater conformational rigidity, starting from 2-aminopyridine, they have also synthesized chiral *P,N*-ligands, which form a five-membered chelate ring with the metal centre. The synthesized ligands **161b**–**d** were tested in the enantioselective Pd-catalysed allylic substitution of 1,3-diphenylprop-2-enyl acetate with dimethyl malonate according to Trost and Murphy’s procedure [137]. The results showed that the phenyl group in **161d** greatly accelerated the reaction and yielded 95% in four hours, but the enantioselectivity remained low (7%). The ligands **161b** and **161c** yielded 91% and 94% respectively with low enantioselectivities 7% ee. Therefore, it is possible to conclude that enantioselectivity of the ligands **161b**–**d** poorly depended on the size of substituent in position-6 of the pyridine ring. The most likely reason would be that the Me, Br and Ph groups at position-6 on the pyridine ring are not sufficient to exert a steric control on the Pd-catalysed allylic alkylation of 1,3-diphenylprop-2-enyl acetate. The NMR spectra of the allylic intermediates [Pd(PhCHCHCHPh){(*S*)-**161b**-(*S*)-**161d**}]^+^ proved that all four possible isomeric forms are present in the same concentration ratio and undergo fast exchange processes [136]. This is the most likely reason for the modest enantioselectivity observed. A chelate ring size became apparent as ligands **161b**–**d** form a six-membered chelate ring, showing less asymmetric induction than those with five-membered chelate rings.

**Figure 36:**
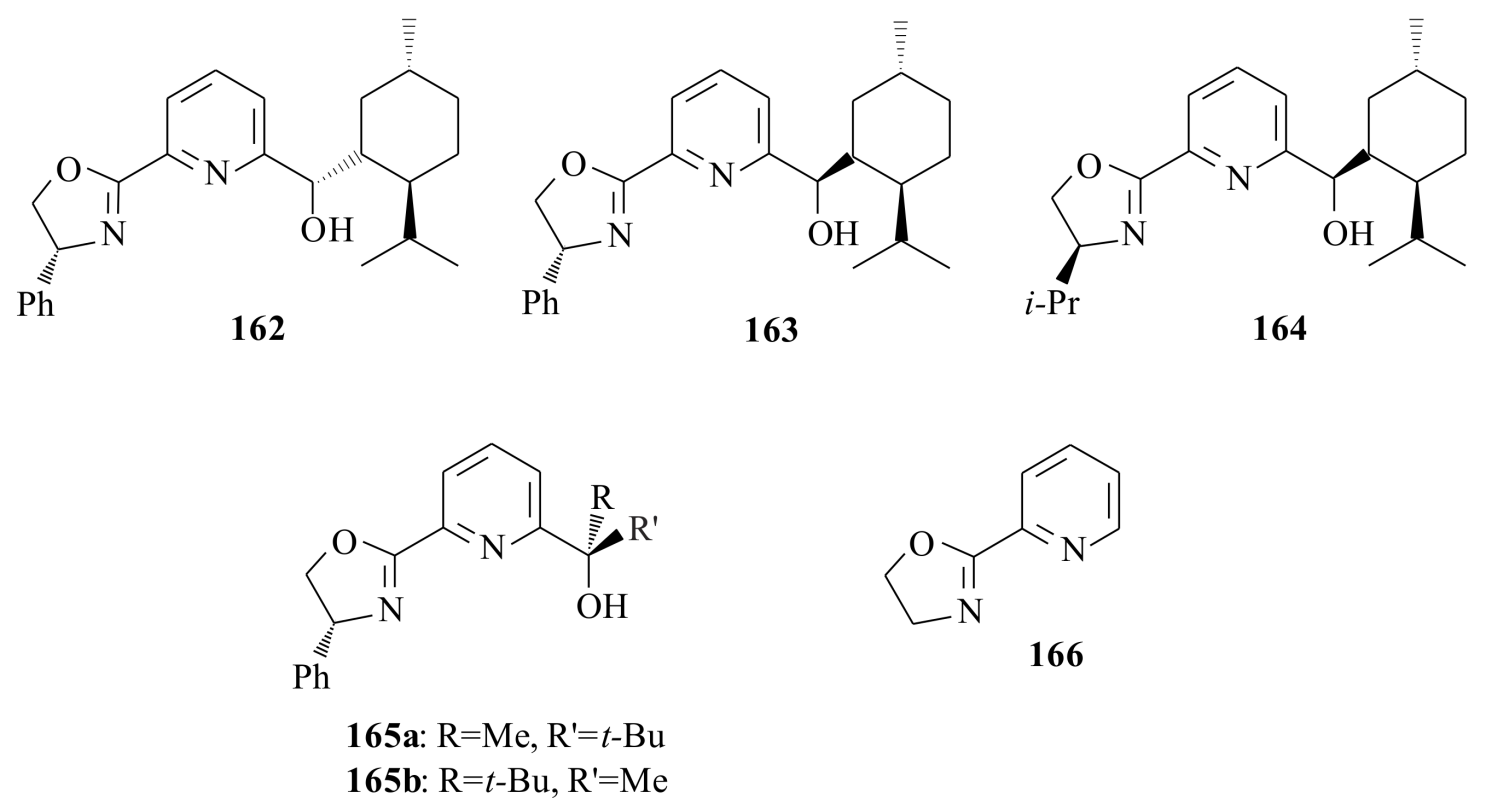
Chiral pyridinooxazoline ligands.

Nordstrom et al. [134] investigated the usefulness of their previously prepared 2-(1-hydroxyalkyl)-6-(4,5-dihydro-2-oxazolyl)pyridines **105**, **106**, **162**–**166** in the palladium catalysed allylation of *rac*-1,3-diphenyl-2-propenyl acetate (Figure 36). This type of ligand has the advantage that large structural variations are possible in that the substituents in the oxazoline ring and in the alcohol part of the molecule as well as the relative configuration of the two stereogenic centres can be varied; in addition, the alcohol group can be alkylated using different electrophiles. A catalytic amount of KOAc, 2.5 mol% [(η^3^-C_3_H_5_)PdCl]_2_, 5 mol% ligand, (MeOCO)_2_CH_2_ and BSA were added to a CH_2_Cl_2_ solution of *rac*-1,3-diphenyl-2-propenyl acetate at room temperature and made to react for four days.

The yields and enantioselectivities of the substitution product in relation to each ligand are provided in Table 11. Even though it took four days to be completed, the reaction proceeded smoothly with most of the ligands, except with **165**, to provide high yields of the product. Ligands with (*S*)-configuration at stereogenic centre in oxazoline ring resulted in the preferred formation of the (*S*)-isomer of the product, while those with (*R*)-configuration resulted in the (*R*)-isomer of the product. Ligands with the same absolute configuration at the two stereogenic centres became even more efficient when changing the *tert*-butyl group to a neomenthyl (comparing ligands **105b** and **106b**, Table 11), while those with the opposite configuration at the two centres became less efficient (comparing ligands **105a** and **106a**, Table 11).

**Scheme 20:**
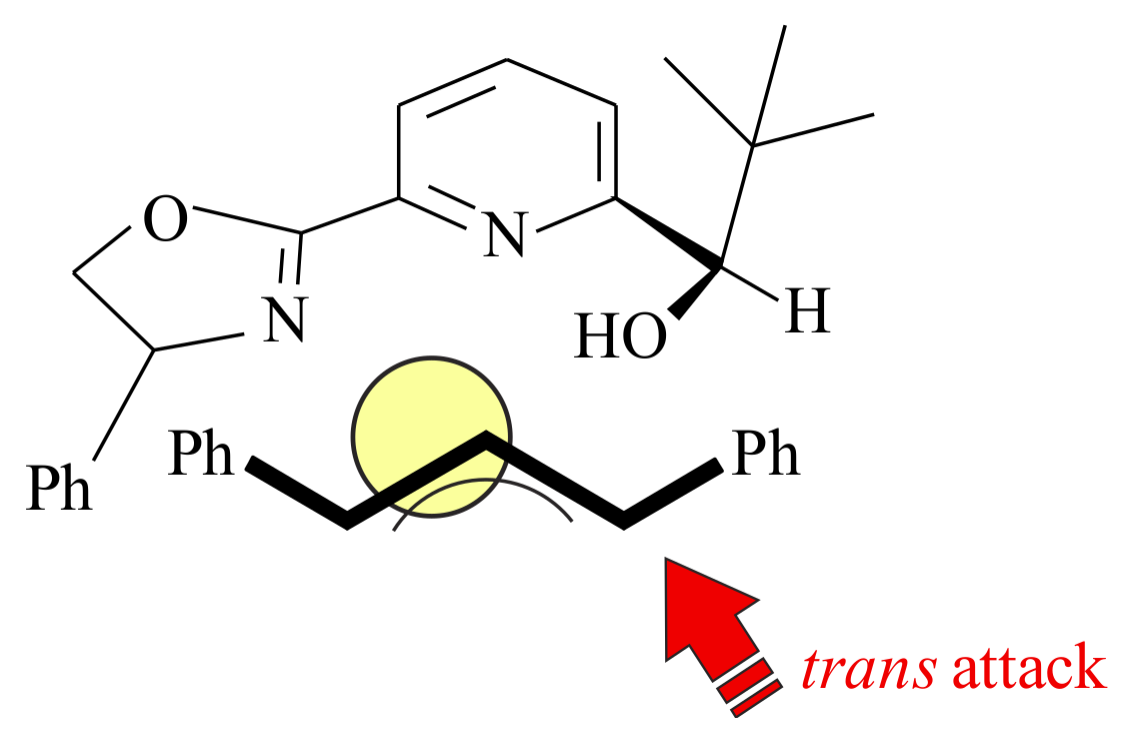
Nucleophilic allylic attack in Pd-catalysed allylic alkylation in the presence of pyridinooxazoline ligands.

According to the conformation of the complex deduced from a Nuclear Overhauser Experiment (NOE), a nucleophilic attack took place *trans* to the oxazoline ring, as shown in Scheme 20 [134].

**Figure 37:**
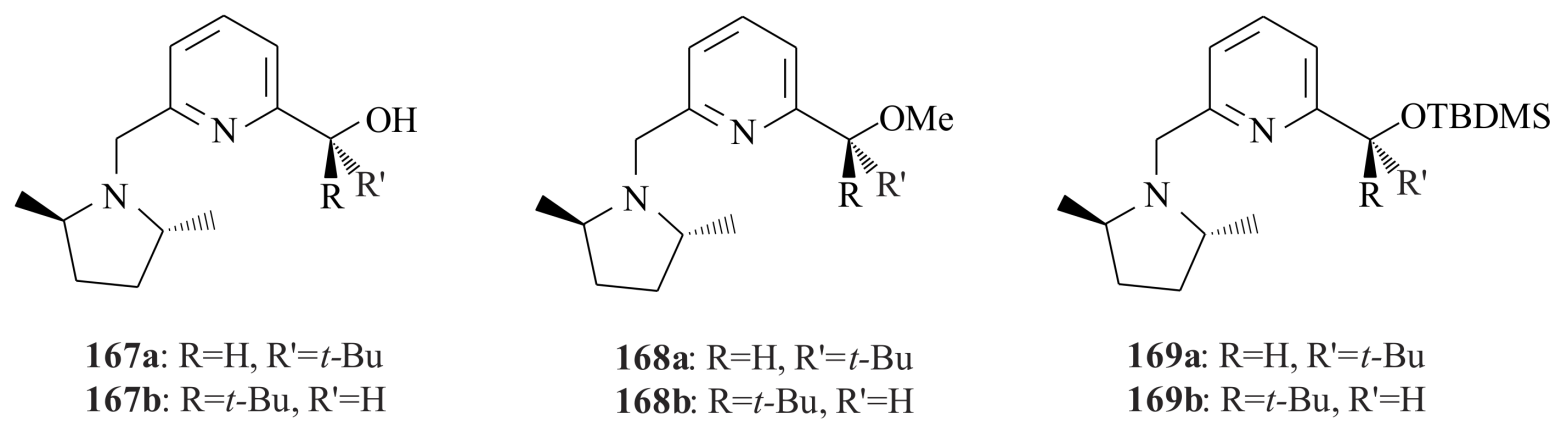
Pyrrolidinopyridines with alkoxy substituents at the 6-position of the pyridine ring.

In order to examine whether the stereochemical control could be extended to other ligand systems, Stranne and Moberg [138] investigated the substitution of *rac*-1,3-diphenylpropenyl acetate with malonate employing 2-pyrrolidinopyridines carrying hydroxyalkyl (**167a**,**b**), methoxyalkyl (**168a**,**b**) and siloxyalkyl (**169a**,**b**) substituents in the 6-position of the pyridine ring (Figure 37). Having this aim, they performed allylic substitution of *rac*-1,3-diphenyl-2-propenyl acetate in CH_2_Cl_2_ at room temperature in the presence of a (π-allyl)palladium-ligand complex generated in situ from 2 mol% of *bis*[(π-allyl)palladium chloride] and 6 mol% of the appropriate ligand. The nucleophile was generated from dimethyl malonate in the presence of *N,O*-*bis*(trimethylsilyl)acetamide (BSA) and a catalytic amount of KOAc. The reactions were run for four days to ensure complete conversion for the less reactive systems. The results of the reaction are presented in Table 12.

The absolute configuration of the product depended on the absolute configuration at the benzylic centre rather than on the configuration of the pyrrolidine ring. Ligands with (*R*)-configuration in the benzylic centre resulted in the (*S*)-product and vice versa and also ligands with (*S*)-configuration at the benzylic centre yielded high ee, which is in complete agreement with Nordstrom et al.’s [134] result. However, the dichotomy previously observed with analogous oxazoline ligands [139] was not observed in this case. Those diastereomers of the ligand assumed to have the bulky groups on the same side of the coordination plane in the intermediate π-allyl palladium complex resulted in high enantioselectivity (80–84% ee), whereas for those assumed to adopt *pseudo*-*C*_2_ conformation, the enantioselectivity is dependent on the nature of the substituent.

In metal complexes containing ligands with two different donor atoms, nucleophilic attack usually takes place *trans* to the better π-acceptor (see Scheme 20). In ligands with donor atoms having similar properties, steric congestion directs attack of the nucleophile [138].

**Figure 38:**
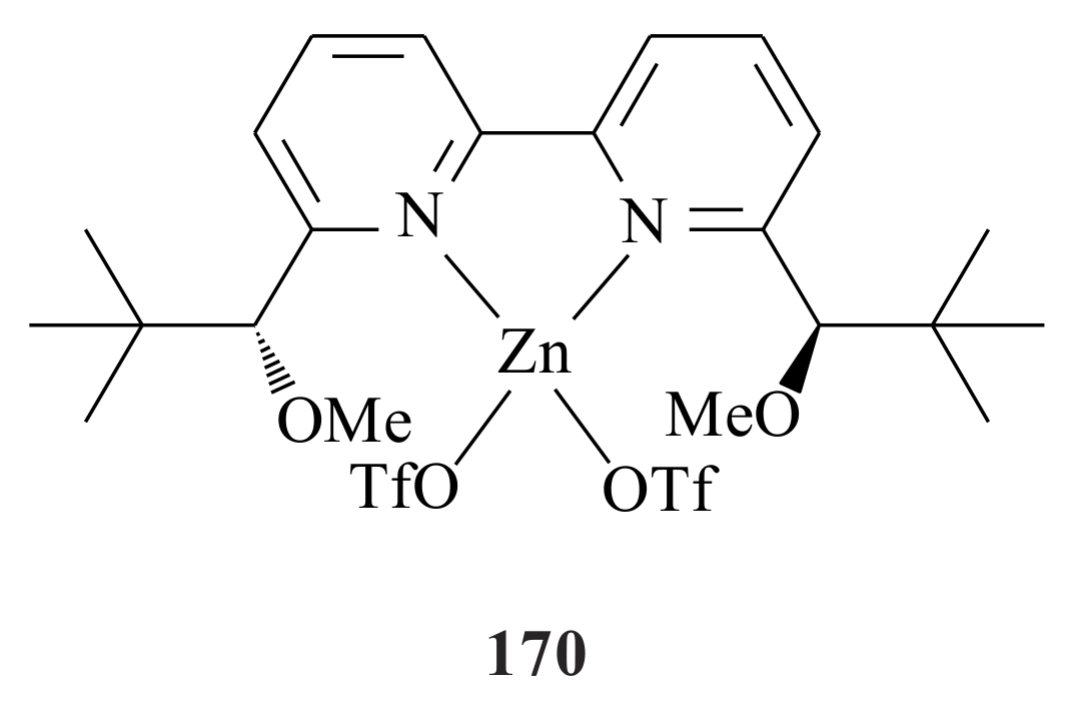
Chiral Zn-bipyridinyl complex.

In order to expand the scope of bipyridinyl ligands, Kwong et al. [140] investigated the synthesis, spectroscopic characterisation and X-ray crystal structure of Zn(L)(OTf)_2_ (L is the methyl ether of **148**). The application of the zinc-complex **170** (Figure 38) as catalyst for allyl addition reactions of aldehydes was also tested. Using 10 mol% of the Zn(L)(OTf)_2_ in the allylation reactions of aromatic, substituted aromatic, aliphatic and heterocyclic aldehydes and allyltributyltin at room temperature in CH_2_Cl_2_ resulted in excellent chemical yield (82–98%) and poor to moderate enantioselectivity (36–59% ee). From the yields of the chemical reaction, it is possible to see that enantioselectivities are independent of the electronic properties of the substituents (electron-withdrawing and electron-donating) in the substituted aromatic aldehydes. However, straight chain and heterocyclic aldehydes showed better enantioselectivities than aromatic aldehydes. It was believed that the chiral Zn-bipyridinyl complex, **170,** functions as Lewis acid in this allylation reaction. Generally, chiral methyl ether of ligand **148** is more efficient than the chiral *bis*(oxazoline) ligand. The X-ray crystal structure of the chloride complex of Zn helped them propose the most likely structure of the Zn(L)(OTf)_2_ complex (**170**). In addition to the enantioselective addition of diethylzinc to benzaldehyde, Eidamshaus and Reissing [103] used the *N-*oxide of pyridinyl alcohol ligand **144b** (10 mol%) in the allylation of benzaldehyde using three equivalents of EtN(*i*-Pr)_2_ and allylsilyltrichloride in CH_3_CN at room temperature that yielded 81% with 24% ee. They also used the TBS (tributylsilyl) protected alcohol of the *N*-oxide **144b** in the same reaction and obtained 12% c.y. with 47% ee. The use of **144b** in the alkynylation of acetylbenzene in the presence of 1M (in hexane) of diethylzinc at room temperature and benzaldehyde resulted in 66% c.y. with 24% ee of the β-substituted acetylbenzene alcohol. Once again, the ligand **144b** (0.4 equiv.) was used in the preparation of β-hydroxy-substituted nitroalkanes (Henry reaction) from benzaldehyde in the presence of CH_3_NO_2_ (10 equiv.), Cu(OTf)_2_ (0.3 equiv.) and one equivalent of EtN(*i*-Pr)_2_ in *i*-PrOH at −50 °C in 18 h. The β-hydroxy-substituted nitroalkane was obtained in good yield (81%) and moderate enantioselectivity (70% ee).

### 2.11. Asymmetric Hydrosilation and Kinetic Resolution of Rac-Benzyl Alcohols

**Figure 39:**
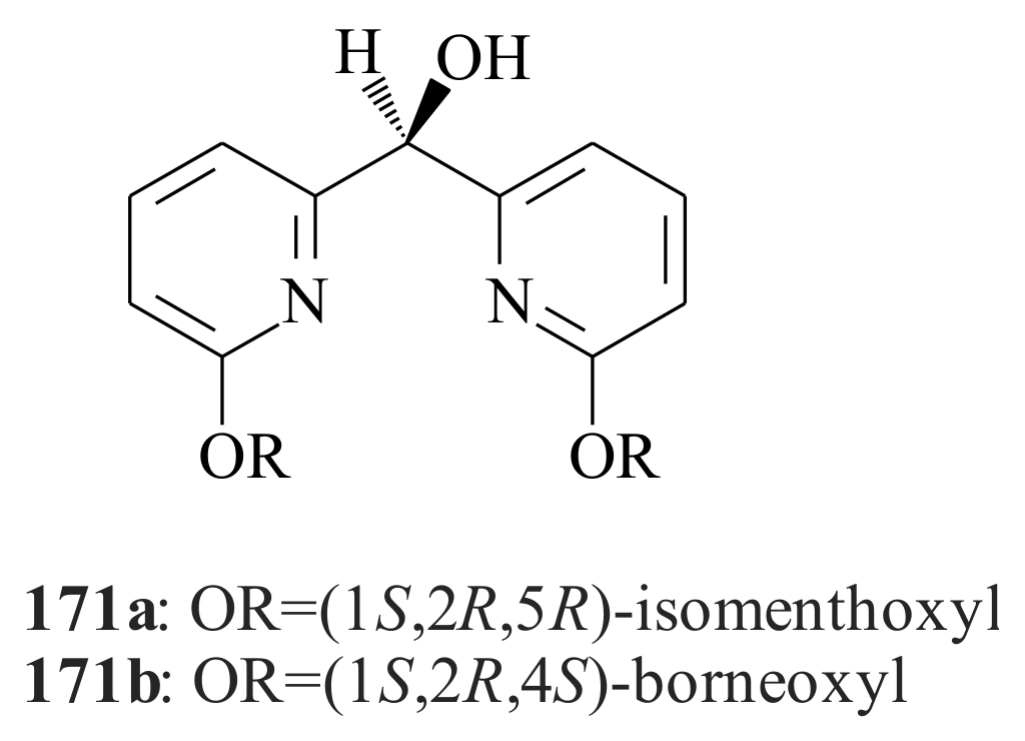
α-Bipyridinyl alcohols with alkoxy groups at the α’-positions of the pyridine ring.

The pyridinyl alcohols **77** and **171a**,**b** (Figure 39) synthesized by Wright et al. [67] were used as ligands in the rhodium-catalysed [(COD)RhCl]_2_ (COD-1,5-cyclooctadiene) asymmetric hydrosilation of acetophenone. The asymmetric catalytic hydrosilation reaction took place between acetophenone and Ph_2_SiH_2_ in the presence of [(COD)RhCl]_2_ catalyst and ligands **171ba**,**b** and/or **77** and acid hydrolysis at −15 °C for 48 h. In this catalytic hydrosilation reaction, ligands **171a**,**b** resulted in greater enantioselectivities (1.0% and 9.0% ee(*S*), respectively) than ligand **77,** which yielded only 0.7% ee of the (*R*)-isomer. This suggested the need for chiral alkoxy groups on both pyridinyl groups on the ligand. The chiral alkoxy group on the pyridine ring has such an effect on the rotational energy barrier about the oxygen-benzylic carbon bond and strongly suggested that the alkoxy moiety is influencing the coordination sphere of the metal.

The use of pyridinyl alcohols or bipyridinyl diols in the area of catalytic asymmetric hydrosilylation reaction is in its infancy as this is the only report we have from literature. Even though the yield and ee’s obtained by using the ligands **171a**,**b** are very low, further investigation of the rhodium catalyst by using different pyridinyl alcohols and bipyridinyl diols would possibly result in a better yield and ee. The work of Wright et al. [67], however, would be a good beginning for further investigation.

**Scheme 21:**
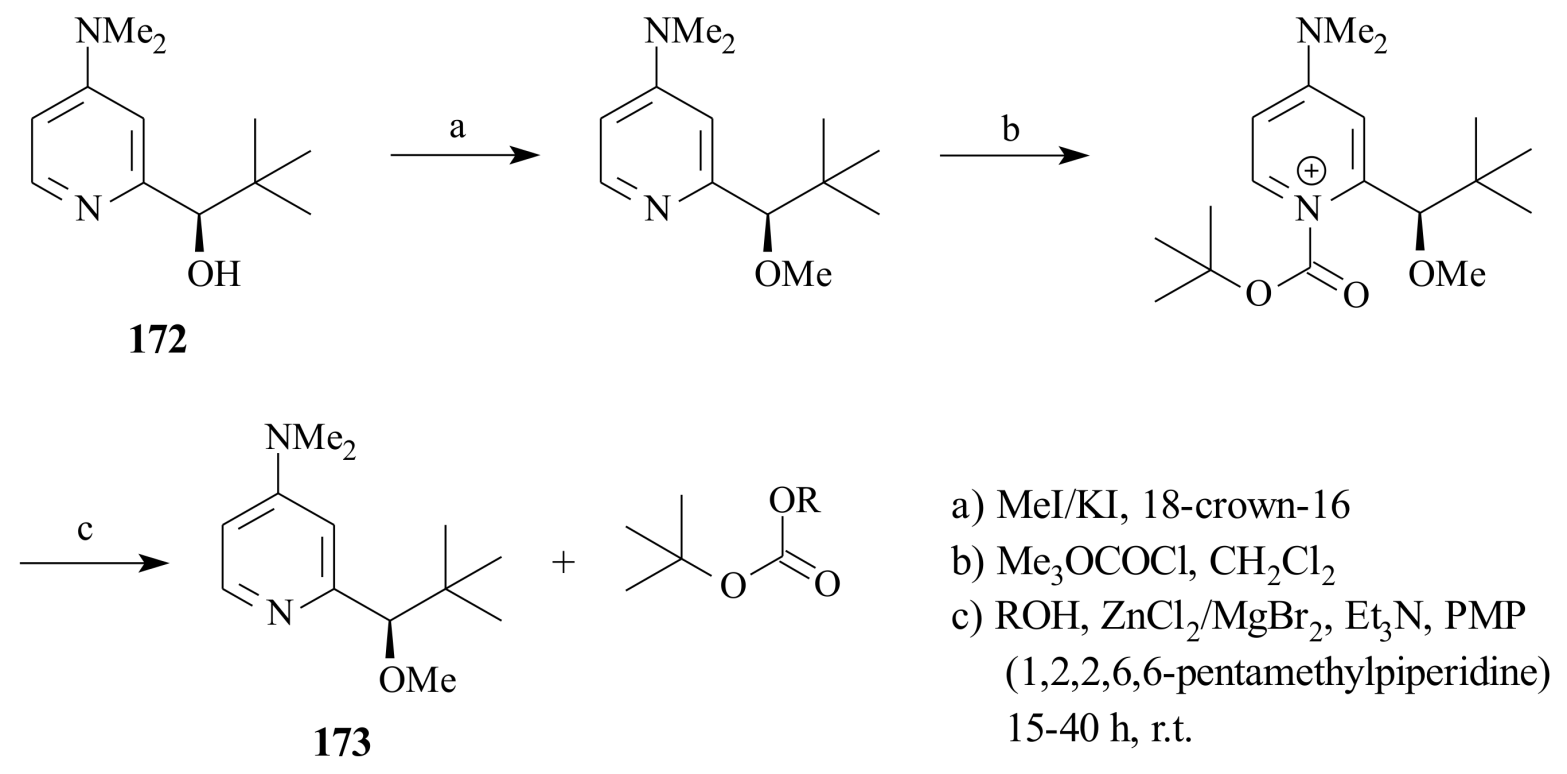
Catalytic role of DMAP alcohol in chiral acyl transfer reaction.

Chiral acyl transfer to a racemic mixture of aromatic secondary alcohols and one *α,β-*unsaturated aliphatic secondary alcohol by *p*-(dimethylamino)pyridine (DMAP) nucleus **172** in the presence of a Lewis acid (e.g., ZnCl_2_ or MgBr_2_) resulted in 76 to 94% ee of the ester (**173**) of the chiral benzylic alcohols with 10 to 54% conversion (see Scheme 21) [141]. Although kinetic resolution of certain chiral alcohols or their ester derivatives is usually carried out by the lipase/esterase family of acyl transfer catalysts, the result obtained by Vedejs and Chen [141] approached some of the lipase methods in enantioselectivity.

The recovery of **173** without change in ee after five repeated acylation cycles made this compound a catalyst. However, this is the only literature we have found in the area of chemical kinetic resolution of racemic alcohols using pyridinyl alcohols. Therefore, further investigation is necessary to get the better kinetically resolving pyridinyl alcohol.

### 2.12. Aqueous Disproportionation of H_2_O_2_

**Figure 40:**
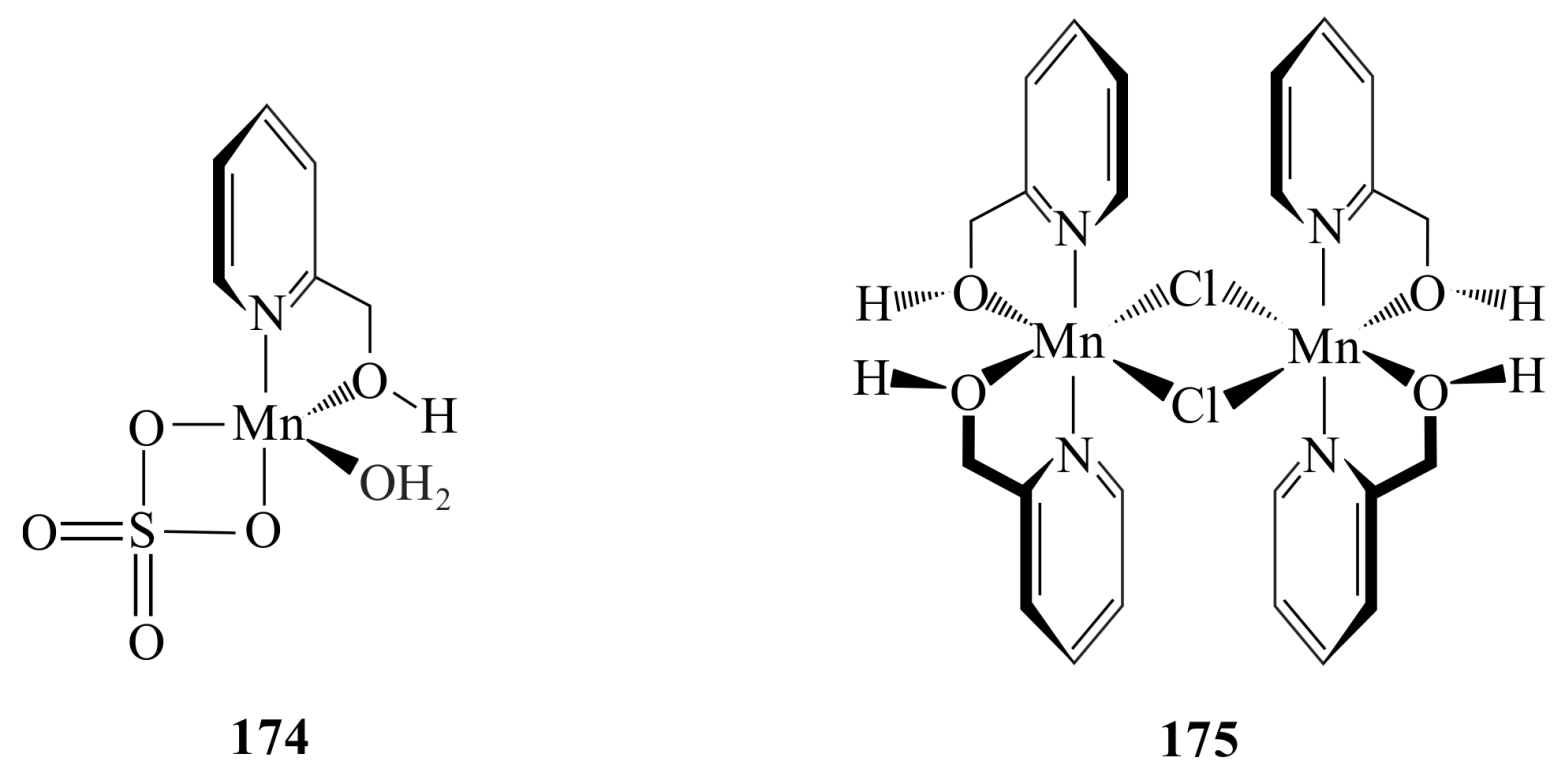
Pyridinyl methanolato complexes of Mn.

Single site pyridin-2-yl-methanol ligand containing complex of manganese(II) complex **174** was synthesized by Zienkiewicz et al. [142] (see Figure 40).The complex exhibited catalase-like activity in neutral pH when tested in the aqueous disproportionation of hydrogen peroxide into water and oxygen. The complex is synthesized in such a way that a water solution of MnSO_4_·H_2_O (2.5 mmol) was added to a solution of pyridin-2-yl-methanol (5 mmol) in 20 mL of pure isopropanol and stirred at room temperature for 5 h to yield 69% of complex. According to Zienkiewicz et al. [142] oxidation of the manganese complex is due to the loss of a bridge oxygen proton which is then followed by peroxide coordination and proton subtraction by the sulphate group mediated by water. A second complex molecule that affects the overall catalytic performance is required for molecular oxygen liberation and catalyst regeneration, due to the reaction rates are diffusion limited.

With the intention to get highly active and structurally different Mn(II) complexes with homologous pyridinyl alcohols having different counter ions and compare their catalytic activity, Zienkiewicz et al. [143] synthesized the two centro-symmetrically related Mn(II) centred complex **175** that is bridged by two chloride ions (see Figure 40).

The Mn(II) ion is surrounded by two chelating pyridin-2-yl-methanol molecules bonded through N and O atoms. The other two chloride ions act as counter ions. The complex is synthesized by dissolving MnCl_2_·4H_2_O (0.2774 g, 1.4 mmol) in 25 mL of analytically pure *i-*PrOH and adding into a 25 mL solution of pyridin-2-yl-methanol (0.4589 g, 4.2 mmol) in *i-*PrOH. The molar ratio of metal to alcohol is 1:3. The mixture is stirred for 5 h and then left to yield 28% of crystallized complex at room temperature. Although this complex follows the same mechanism as the single site Mn(II) complex **174**, it is found to be more active in disproportionation reaction of H_2_O_2_ to harmless H_2_O and O_2_.

### 2.13. Arylation of Aromatic Compounds

**Scheme 22:**
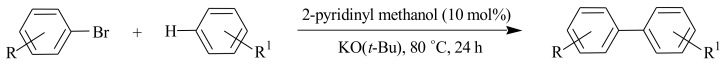
Transition metal free direct arylation of unactivated arenes.

A general C-H metal-free direct arylation of unactivated benzene (Scheme 22) with a wide range of aryl/heteroaryl bromides in the presence of pyridin-2-yl-methanol and potassium *tert*-butoxide resulted in 55–88% coupled products under mild (80 °C) reaction conditions [144]. The use of simple and inexpensive pyridinyl alcohol catalyst tackles the challenging C-H arylation under transition-metal-free conditions significantly contributing to green chemistry. Apart from benzene, other unactivated arenes are also examined and the yield obtained is very good (48–78%).

### 2.14. Carbonylative Hydroesterification of Alkenes/Alkynes

Armanino et al. [145] used Ru_3_(CO)_12_ and Bu_4_NI together with pyridin-2-yl-methylformate in carbonylative hydroesterification reaction of alkenes and alkynes without external CO atmosphere. The catalyst, however, did not show ligand effects and hence poorly defined catalytic species. Li et al. [146] reported a similar carbonylative hydroesterification of alkenes in the presence of Ru_3_(L) catalyst (where L = NHC and phosphine) and KO^t^Bu where the ligands facilitate access to ligand-ligand selective hydroesterification resulting in isomeric (linear versus branched) esters.

**Scheme 23:**
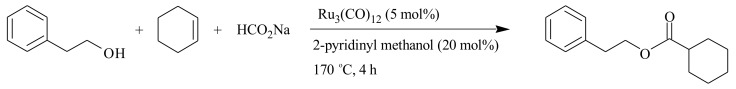
Carbonylative hydroesterification of cyclohexene.

Kim et al. [147], on the other hand, reported a transition-metal-catalyzed carbonylative hydroesterification reaction of alkenes with sodium formate in the presence of pyridin-2-yl-methanol (Scheme 23). The pyridinyl alcohol served as a chelation assistant and the sodium formate as the carbon monoxide source. Other than obtaining a better yield (up to 97%) they also proposed a mechanism for the reaction.

### 2.15. Ethylene Oligomerization

**Scheme 24:**
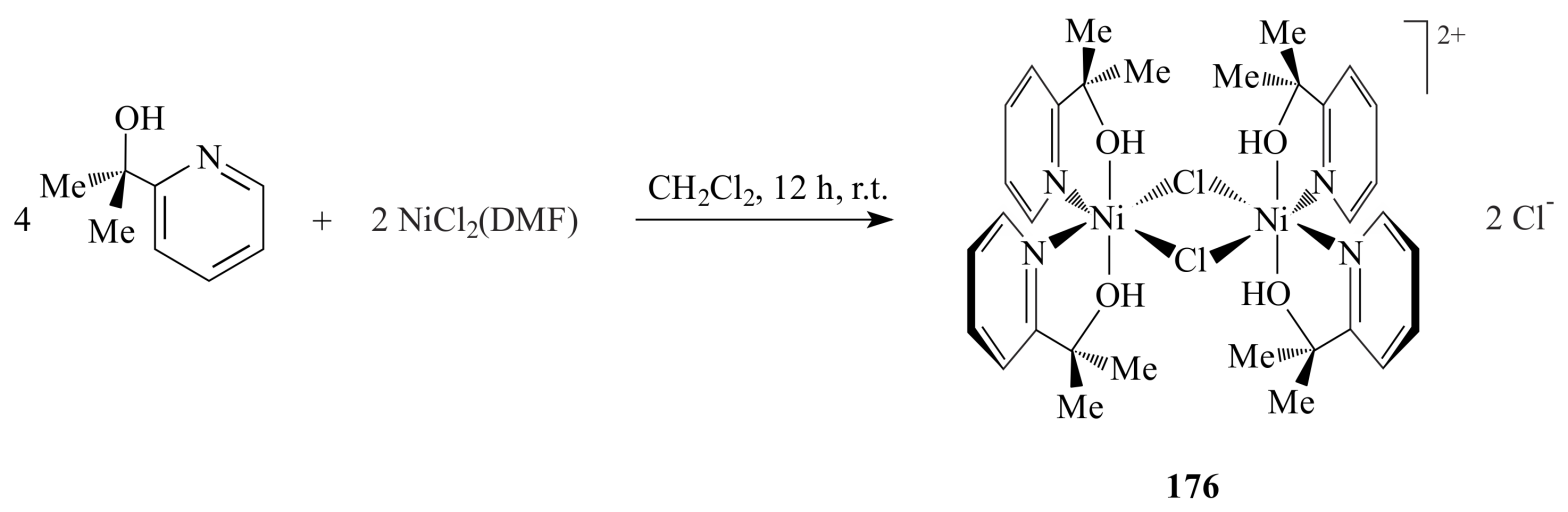
Synthesis of complex **176**.

Oligomerization of ethylene in the presence of 200 equivalents of MAO and the precatalyst **176** led to the formation of butenes (87%) with a selectivity for 1-butene within the C_4_ fraction of 58% [148]. The synthesis of the precatalyst **176** is performed as shown in Scheme 24.

The activity and turnover frequency (up to 20,400 mol of C_2_H_4_ (mol Ni.h)^−1^ of **176** increased upon increasing the amount of MAO to 800 equivalents. In the presence of 6 equivalents of EtAlCl_2_ the activity was considerably increased to 97 1000 mol of C_2_H_4_/(mol of Ni·h) with a selectivity for C_4_ olefins of 64% whereas the selectivity for 1-butene with in the C_4_ fraction decreased to 9%. This low selectivity was attributed to the reversible *β*-H elimination after ethylene insertion and reinsertion of the olefin with the opposite regiochemistry to give 2-butene after chain transfer or to isomerization of 1-butene by reuptake mechanism. It was also anticipated that the precatalyst is not retaining its dinuclear nature in the course of catalysis owing to the presence of the Lewis acid co-catalyst that will tend to open the chloride bridges. However the dinuclear structure provides an interesting “reservoir” of active species. In these systems a less basic nitrogen moiety leads to increased *α*-olefin selectivity and to higher turnover frequencies.

**Scheme 25:**
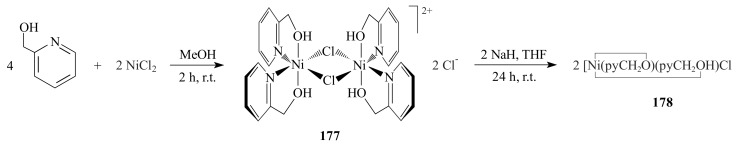
Synthesis of complexes **177** and **178**.

Complexes **177** and **178** were synthesized as shown in Scheme 25 and tested in ethylene oligomerization with EtAlCl_2_ (Al/Ni ratios of 2, 4 or 6) or MAO (50, 100 or 200 equivalents) as cocatalysts under 10 bar of ethylene [149]. The oligomerization results obtained are mostly dimers and trimers. Complex **177** in the presence of 6 equivalents of EtAlCl_2_ found to be the most active system with a turnover frequency up to 187 500 C_2_H_4_ (mol Ni.h)^−1^. The same complex with 200 equivalents of MAO was also found to be active, with a turnover frequency up to 104 300 C_2_H_4_ (mol Ni.h)^−1^ under 30 bar of ethylene. Complexes **177** and **178** in MAO showed higher activities than complex **176**.

## 3. Conclusions

The application of pyridinyl alcohols in the areas of catalysis, coordination chemistry, and organic synthesis is tremendous. Of the multifaceted applications of the pyridinyl alcohols, the lions share is taken by the preparation of their transition metal complexes for the homogeneous and chemical asymmetric catalysis that included; metathesis, olefin polymerization, olefin epoxidation, cyclopropanation, addition of organozinc compounds to chalcones, enantioselective addition of organozinc compounds to aldehydes, allylic oxidation, ring-opening reactions of *meso*-epoxides, aldol reactions, allylic alkylation, asymmetric hydrosilation, kinetic resolution of *rac*-benzyl alcohols, aquous disproportionation of H_2_O_2_, arylation of aromatic compounds, carbonylative hydro-esterification of alkenes/alkynes, and ethylene oligomerization. The transition metal complexes with pyridinyl alcoholato ligands in most cases and the pyridinyl alcohols alone were used as catalysts in the homogeneous and chemical asymmetric reactions. Although a number of investigations were undertaken into the enantioselective addition of Et_2_Zn to aliphatic and aromatic aldehydes compared to others, there seems no definite conclusion as to why the c.y. and ee variations occur upon using pyridinyl alcohols having similar structures. In addition to this, the effect of substituents at 6-position of the pyridinyl alcohols with regard to enantioselectivity and chemical yields in the diethylzinc addition reactions is still controversial. The effort to come up with good c.y.’s and ee’s by Herrmann et al. [28] and Lobmaier et al. [60] in the epoxidation of terminal olefins by molecular oxygen was remarkable; however, the yields and enantioselectivities obtained need further improvement. The ability to obtain the two isomers of the alcohol in the ring-opening reactions of *meso*-epoxides by using bipyridinyl diols and Cu(DS)_2_ and Sc(DS)_3_ catalysts is very significant. The result obtained by Vedejs and Chen [141] in the chemical kinetic resolution of *rac-*benzyl alcohols by pyridinyl alcohols with DMAP core as the sole catalyst approached some of the lipase methods in enantioselectivity. This is an encouraging result; however, no sufficient investigation has been done in this regard. The use of simple and inexpensive pyridin-2-yl methanol catalyst by Wu and co-workers [144] enabled them to tackle the challenging C-H arylation under transition-metal-free conditions significantly contributing to green chemistry. The contribution of Zienkiewicz et al. [142] in synthesizing Mn(II)-complex that exhibited catalase-like activity in neutral pH when tested in the aqueous disproportionation of hydrogen peroxide into water and oxygen is remarkable.

The multidentate character of the pyridinyl alcohols and/or bipyridinyl diols enabled them complex with the varied number and type of transition metals and therefore catalysed a large number of homogeneous and catalytic asymmetric reactions. On the other hand, in the metathesis and in most of the catalytic enantioselective homogeneous reactions, a few pyridinyl alcohols and/or bipyridinyl diols were used in the preparation of chiral catalysts that led to limited investigation. In addition to these, the electronic effect of substituents on the pyridine ring of the pyridinyl alcohol or the bipyridinyl diols was not given due attention or was less investigated. It is therefore worthwhile to consider pyridinyl alcohols and/or bipyridinyl diols with electron-donating and electron-withdrawing substituents on the pyridine ring(s) and further investigate the c.y.’s as well as ee’s of the homogeneous and asymmetric chemical reactions.

## Figures and Tables

**Table 1:** Yields in the synthesis of pyridine alkoxide substituted precatalysts **20a**–**f** [35].

Complex	Ligand	Product	Ligand(L)	X	X’	R	Yield (%)
**18a**	**19a**	**20a**	PCy_3_	Ph	H	Me	76
**18b**	**4**	**20b**	SIMes	Ph	H	Me	86
**18c**	**19a**	**20c**	SIMes	Ph	H	Ph	53
**18d**	**19a**	**20d**	SIMes	indenylidene		Me	75
**18e**	**19a**	**20e**	SIMes	indenylidene		Me	82
**18f**	**19a**	**20f**	SIMes	SPh	H	Me	94

**Table 2:** Asymmetric epoxidation of trans-methylstyrene with *t-*BuOOH/CHP and **66**–**68**.

Catalyst	*t-*BuOOH, 50 °C		*t*-BuOOH, 70 °C		CHP, 50 °C		CHP, 70 °C	
	Conv. ^1^	ee ^1^	Conv. ^1^	ee ^1^	Conv. ^1^	ee ^1^	Conv. ^1^	ee ^1^
**66**	**38**	**0**	**65**	**0**	**29**	**0**	**58**	**0**
**67**	**29**	**8 *R,R***	**52**	**7 *R,R***	**20**	**17 *R,R***	**47**	**23 *R,R***
**68**	**32**	**7 *S,S***	**56**	**5 *S,S***	**23**	**8 *S,S***	**52**	**6 *S,S***

^1^ Both conv. and ee are in %.

**Table 3:** Effect of nickel and ligand concentration (in acetone, −30 °C, 18 h) [74].

Entry	Ligand (mol%)	Ni(acac)_2_ (mol%)	Product Yield (%)	Product ee (%)
**1**	**88(30)**	**1**	**55**	**72**
**2**	**88(20)**	**1**	**75**	**72**
**3**	**88(10)**	**1**	**82**	**54**
**4**	**88(5)**	**1**	**74**	**20**
**5**	**88(10)**	**2**	**66**	**48**
**6**	**88(6)**	**2**	**73**	**18**
**7**	**88(15)**	**5**	**58**	**58**
**8**	**88(5)**	**5**	**69**	**18**
**9**	**84 ^1^(30)**	**1**	**62**	**86**
**10**	**84 ^1^(20)**	**1**	**79**	**82**
**11**	**84 ^1^(10)**	**1**	**81**	**53**

^1^ 92% ee of ligand was used.

**Table 4:** Variation of ligand structure (in acetonitrile at −30°C, ca. 18 h) [74].

Entry	Ligand, ee (%)	Ligand/Ni Ratio ^1^	Product Yield (%)	Product ee (%)
**1**	**88, >98**	**20**	**75**	**72**
**2**	**84, 92**	**19**	**79**	**82**
**3**	**85a, 90**	**22**	**84**	**86**
**4**	**85b, 96**	**20**	**64**	**82**
**5**	**89 ^2^, 92**	**20**	**75**	**60**
**6**	**85c, 70**	**22**	**72**	**2**
**7**	**90, 68**	**22**	**79**	**2**

^1^ Use of 1 mol% of Ni(acac)_2_. ^2^ Contained small amount of the corresponding chloride.

**Table 5:** Asymmetric addition of ZnEt_2_ to aldehydes [81].

Ligand	Aldehyde	Conv. (%) ^1^	Alcohol ee (%)	Conf.
**57a**	**Benzaldehyde**	**93**	**21**	***S***
**95**	**Benzaldehyde**	**93**	**38**	***R***
**57b**	**Benzaldehyde**	**93**	**44**	***R***
**96**	**Benzaldehyde**	**100**	**82**	***R***
**57a**	**3-Phenylpropanal**	**89**	**26**	***S***
**95**	**3-Phenylpropanal**	**81**	**20**	***R***
**57b**	**3-Phenylpropanal**	**87**	**38**	***R***
**96**	**3-Phenylpropanal**	**87**	**63**	***R***
**57a**	**3-Phenylpropynal**	**91**	**9**	***S***
**95**	**3-Phenylpropynal**	**95**	**1**	***R***
**57b**	**3-Phenylpropynal**	**92**	**21**	***R***
**96**	**3-Phenylpropynal**	**90**	**43**	***R***

^1^ GLC conversion.

**Table 6:** Enantioselective addition of diethylzinc to aldehydes using 5 mol% of **48** [82].

Aldehyde	Solvent	Temp (°C)	Time (h)	c.y. (%)	ee (%)
**PhCHO**	**Toluene**	**0**	**3**	**96**	**83**
***p*-ClPhCHO**	**Toluene**	**0**	**3**	**93**	**65**
***p*-CH_3_OPhCHO**	**Toluene**	**0**	**9.5**	**90**	**96**
**n-C_6_H_13_CHO**	**Toluene**	**0**	**3**	**54**	**75**
**H_2_C=CH(CH_2_)_2_CHO**	**Hexane**	**0**	**3**	**70**	**83**
**E-PhCH=CHCHO**	**Toluene**	**0**	**3**	**28**	**76**
**PhC=CCHO**	**Toluene**	**0**	**3**	**25**	**88**

**Table 7:** Catalyst variation in the alkylation of benzaldehyde with ZnEt_2_ [82].

Catalyst	ee (%)	mol (%)	Time (h)	c.y. (%)	ee (%)	Conf.
**48**	**>98**	**5**	**3**	**83**	**86**	***R***
**84**	**90**	**8**	**3**	**73**	**88**	***R***
**85b**	**96**	**5**	**3**	**51**	**78**	***R***
**98**	**98**	**5**	**3**	**77**	**88**	***S***
**80**	**90**	**11**	**3**	**80**	**78**	***R***
**97**	**92**	**5**	**6**	**58**	**74**	***S***
**89**	**90**	**10**	**6**	**65**	**81**	***R***
**96**	**90**	**11**	**6**	**61**	**74**	***R***
**90**	**70**	**10**	**6**	**64**	**5**	***R***
**99**	**84**	**10**	**6**	**24**	**15**	***R***

**Table 8:** Asymmetric addition of diethylzinc to aldehydes catalysed by chiral pyridinyl alcohols [15].

Ligand	Aldehyde	c.y. (%)	ee (%)	Conf.
**105a**	**Benzaldehyde**	**85**	**81**	***S***
**105a**	***p*-Chlorobenzaldehyde**	**40**	**76**	***S***
**106a**	**Benzaldehyde**	**68**	**40**	***R***
**105b**	**Benzaldehyde**	**95**	**88**	***R***
**105b**	***p*-Chlorobenzaldehyde**	**83**	**81**	***R***
**106b**	**Benzaldehyde**	**84**	**71**	***S***
**104**	**Benzaldehyde**	**24**	**26**	***R***

**Table 9:** Asymmetric alkylation of substituted benzaldehydes using Et_2_Zn in the presence of ligand **139**.

Substituent	c.y. (%)	ee (%)	Conf.
**H**	**83**	**75**	***S***
***o*-OMe**	**85**	**56**	***S***
***p*-OMe**	**66**	**58**	***S***
***m*-Cl**	**36**	**62**	***S***
***p*-Cl**	**42**	**63**	***S***
***o*-Me**	**37**	**78**	***S***
***p*-Me**	**31**	**65**	***S***

**Table 10:** Enantioselective aldol addition of trichlorosilyl enolate of methyl acetate to acetophenone.

Catalyst	Yield (%)	er	Conf.
**155a**	**92**	**77.5/22.5**	**S**
**155b**	**94**	**82.0/18.0**	**S**
**156a**	**90**	**87.0/13.0**	**S**
**156a**	**94** ^1^	**92.0/8.0**	**S**
**157**	**89**	**71.5/28.5**	**R**
**156b**	**90**	**90.0/10.0**	**S**

^1^ Reaction at −20 °C for 12 h.

**Table 11:** Enantioselective allylation of *rac*-1,3-diphenyl-2-propenyl acetate in dimethyl malonate using ligands **105**, **106**, **162**–**166**.

Ligand	Yield (%)	ee (%)
**105a**	**96**	**91 (*S*)**
**105b**	**67**	**15 (*R*)**
**106a**	**79**	**78 (*S*)**
**106b**	**81**	**95 (*R*)**
**162**	**n.d.**	**39 (*R*)**
**163**	**83**	**>99 (*R*)**
**164**	**91**	**45 (*S*)**
**165a**	**0**	
**165b**	**0**	
**166**	**97**	**50 (*R*)**

**Table 12:** Enantioselective allylation of rac-1,3-diphenylpropenyl acetate with malonate employing 2-pyrrolidinopyridines **167**–**169**.

Ligand	Yield (%)	ee (%)	Conf.
**167a**	**13**	**27**	**S**
**167b**	**Quant.**	**84**	**R**
**168a**	**Quant.**	**74**	**S**
**168b**	**Quant.**	**83**	**R**
**169a**	**4**	**8**	**S**
**169b**	**85**	**80**	**R**

## Abbreviations

ADMET	acyclic diene metathesis polymerization
BD	1,3-butadiene
BMP	2,6-*bis*(1*S*,2*S*,5*R*)-(−)-menthoxypyridinyl
BSA	*bis*(trimethylsilyl) acetamide
Cat.	catalyst
CHP	cumylhydroperoxide
COD	1,5-cyclooctadiene
Conf.	configuration
Conv.	conversion
CM	cross metathesis
Cp	cyclopentadiene
CPPA	*cis*-1-propenylphosphonic acid
c.y.	chemical yield
DMAP	dimethylamino pyridine
DME	1,2-dimethoxyethane
DMF	dimethylformamide
DS	dodecylsulphate (OSO_3_C_12_H_25_)
EASC	ethylaluminum sesquichloride
EDA	ethyldiazoacetate
ee	enantiomeric excess
equiv.	equivalent
er	enantiomeric ratio
FG	functional group
GLC	gas-liquid chromatography
Gr1	benzylidene-dichloro[*bis*(tricyclohexylphosphine)]ruthenium (Grubbs 1)
Gr2	benzylidene-dichloro(tricyclohexylphosphine)[(1,3-*bis*(2,4,6-trimethylphenyl)-2-imidazolidinylidenene]ruthenium (Grubbs 2)
HCC	homogeneous chiral catalyst
Hex	hexane
Lig	ligand
Ln	lanthanide
MAO	methylalumoxane
n.d.	not determined
NHC	N-heterocyclic carbene
OTf	triflate (trifluoromethanesulfonate)
PCy_3_	tricyclohexylphosphine
PMBOH	*p*-methoxybenzyl alcohol
PMP	1,2,2,6,6-pentamethylpiperidine
py	pyridine
Quant.	quantitative
RCM	ring-closing metathesis
RE	rare earth
ROCM	ring-opening cross metathesis
ROM	ring-opening metathesis
ROMP	ing-opening metathesis polymerization
r.t.	room temperature
SIMes	*N,N*’-*bis*(2,4,6-trimethylphenyl)imidazolidin-2-ylidene
TBDMS	tertiary butyldimethylsilyl group
TBHP	tertiary butylhydroperoxide
TBS	tertiary butyldimethylsilyl
Temp.	temperature
TLC	thin-layer chromatography
Tol.	toluene
tol	tolyl
TON	turnover number
